# Fucosylation of HLA-DRB1 regulates CD4^+^ T cell-mediated anti-melanoma immunity and enhances immunotherapy efficacy

**DOI:** 10.1038/s43018-022-00506-7

**Published:** 2023-01-23

**Authors:** Daniel K. Lester, Chase Burton, Alycia Gardner, Patrick Innamarato, Krithika Kodumudi, Qian Liu, Emma Adhikari, Qianqian Ming, Daniel B. Williamson, Dennie T. Frederick, Tatyana Sharova, Michael G. White, Joseph Markowitz, Biwei Cao, Jonathan Nguyen, Joseph Johnson, Matthew Beatty, Andrea Mockabee-Macias, Matthew Mercurio, Gregory Watson, Pei-Ling Chen, Susan McCarthy, Carlos MoranSegura, Jane Messina, Kerry L. Thomas, Lancia Darville, Victoria Izumi, John M. Koomen, Shari A. Pilon-Thomas, Brian Ruffell, Vincent C. Luca, Robert S. Haltiwanger, Xuefeng Wang, Jennifer A. Wargo, Genevieve M. Boland, Eric K. Lau

**Affiliations:** 1 Department of Tumor Biology, H. Lee Moffitt Cancer Center and Research Institute, Tampa, FL USA; 2 Cancer Biology Ph.D. Program, University of South Florida, Tampa, FL USA; 3 Molecular Medicine Program, H. Lee Moffitt Cancer Center and Research Institute, Tampa, FL USA; 4 Department of Immunology, H. Lee Moffitt Cancer Center and Research Institute, Tampa, FL USA; 5 Immunology Program, H. Lee Moffitt Cancer Center and Research Institute, Tampa, FL USA; 6 Department of Drug Discovery, H. Lee Moffitt Cancer Center and Research Institute, Tampa, FL USA; 7 Complex Carbohydrate Research Center, the University of Georgia, Athens, GA USA; 8 Department of Surgery, Massachusetts General Hospital, Boston, MA USA; 9 Department of Surgical Oncology, MD Anderson Cancer Center, Houston, TX USA; 10 Department of Cutaneous Oncology, H. Lee Moffitt Cancer Center and Research Institute, Tampa, FL USA; 11 Department of Biostatistics and Bioinformatics, H. Lee Moffitt Cancer Center and Research Institute, Tampa, FL USA; 12 Advanced Analytical and Digital Laboratory, H. Lee Moffitt Cancer Center and Research Institute, Tampa, FL USA; 13 Department of Analytic Microscopy, H. Lee Moffitt Cancer Center and Research Institute, Tampa, FL USA; 14 Department of Pathology, H. Lee Moffitt Cancer Center and Research Institute, Tampa, FL USA; 15 Department of Diagnostic Imaging, H. Lee Moffitt Cancer Center and Research Institute, Tampa, FL USA; 16 Proteomics and Metabolomics Core, H. Lee Moffitt Cancer Center and Research Institute, Tampa, FL USA; 17 Department of Molecular Oncology, H. Lee Moffitt Cancer Center and Research Institute, Tampa, FL USA; 18 Department of Genomic Medicine, MD Anderson Cancer Center, Houston, TX USA; 19 Broad Institute of Harvard and Massachusetts Institute of Technology, Massachusetts General Hospital, Boston, MA USA

**Keywords:** Tumour immunology, Melanoma, Cancer, Cancer immunotherapy, Dietary carbohydrates

## Abstract

Immunotherapy efficacy is limited in melanoma, and combinations of immunotherapies with other modalities have yielded limited improvements but also adverse events requiring cessation of treatment. In addition to ineffective patient stratification, efficacy is impaired by paucity of intratumoral immune cells (itICs); thus, effective strategies to safely increase itICs are needed. We report that dietary administration of l-fucose induces fucosylation and cell surface enrichment of the major histocompatibility complex (MHC)-II protein HLA-DRB1 in melanoma cells, triggering CD4^+^ T cell-mediated increases in itICs and anti-tumor immunity, enhancing immune checkpoint blockade responses. Melanoma fucosylation and fucosylated HLA-DRB1 associate with intratumoral T cell abundance and anti-programmed cell death protein 1 (PD1) responder status in patient melanoma specimens, suggesting the potential use of melanoma fucosylation as a strategy for stratifying patients for immunotherapies. Our findings demonstrate that fucosylation is a key mediator of anti-tumor immunity and, importantly, suggest that l-fucose is a powerful agent for safely increasing itICs and immunotherapy efficacy in melanoma.

## Abstract (web summary)

Lau and colleagues show that the dietary addition of the sugar l-fucose enhances immunotherapy response in melanoma models. This is mediated via the fucosylation and cell surface enrichment of HLA-DRB1 and induction of anti-tumor immunity.

## Main

Melanoma is one of the deadliest skin cancers, with an estimated ~99,780 new diagnoses and ~7,650 deaths in 2022 in the United States alone (American Cancer Society Facts and Figures, 2022). Despite reports of striking efficacy, durable immunotherapy responses have been limited to subsets of patients^1,2^. In an attempt to improve responses, clinical trials have tested combinations of immunotherapies with other therapeutic interventions, with limited success^1,3^. Unfortunately, patients often experience substantial adverse events, sometimes resulting in cessation of treatment. Ineffective patient stratification is another ongoing challenge for the effective administration of immunotherapies. Although biomarkers of responsiveness remain under active investigation, one commonality of poor response is insufficient abundance and tumor-suppressive activity of itICs^4^. Therefore, elucidating itIC biology and developing safe and effective strategies to increase tumor-suppressive itICs are crucial for improving the efficacy of immunotherapies and related biomarkers.

Fucosylation, the conjugation of glycoproteins with the sugar l-fucose (l-fuc) at asparagine or serine–threonine residues (*N*- or *O*-linked, respectively) is mediated by 13 fucosyltransferases (FUTs) and impacts protein functions that are crucial for immune and developmental processes^5,6^. Whereas altered fucosylation has been reported in a number of cancers, our understanding of its mechanisms and functional contributions is limited^7,8^. We previously found that global fucosylation decreases during melanoma progression, and increased tumor fucosylation levels correlate with favorable patient-survival outcomes^9^. Furthermore, increasing melanoma fucosylation in a syngeneic mouse model reduced tumor growth and metastasis and significantly increased itICs. How fucosylation regulates anti-tumor immunity, however, was unknown. Here, we report that dietary l-fuc can regulate the biology and interactions between CD4^+^ T and melanoma cells via cell surface stabilization of an MHC-II protein, which robustly induces itICs and anti-melanoma immunity. Tumoral MHC-II protein expression, which is known to trigger CD4^+^ T cell-mediated responses, is associated with immune-mediated tumor suppression and increased responsiveness to immunotherapies^10,11^. Our findings demonstrate the ability of l-fuc to improve the efficacy of immunotherapies by promoting MHC-II–CD4^+^ T cell-mediated responses and identify fucosylation-based biomarkers that may enhance patient stratification.

## Results

### Increased fucosylation blunts melanoma growth and increases itICs

**Fig. 1:**
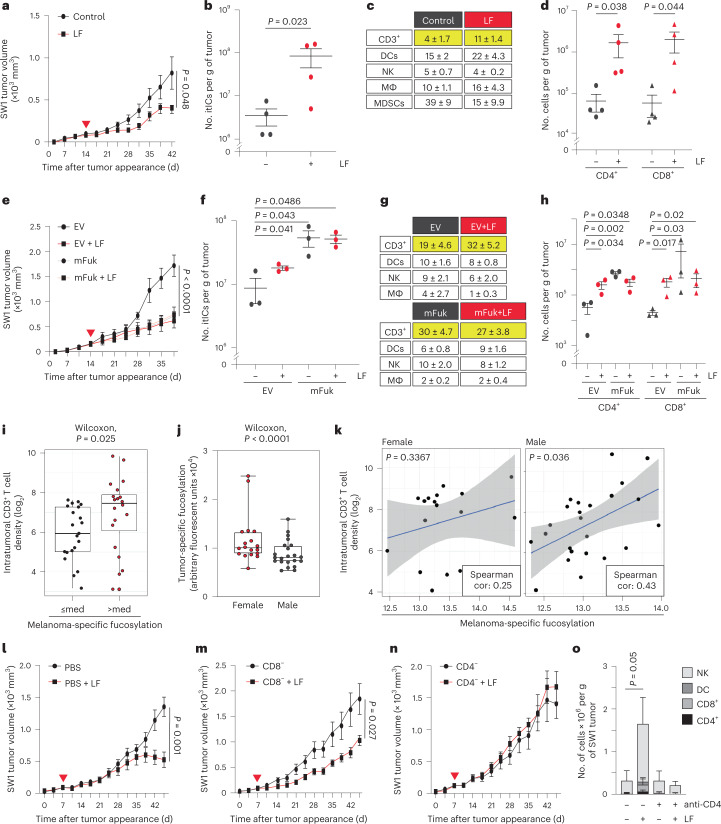
Increasing melanoma fucosylation reduces tumor growth and increases itIC abundance, particularly CD4^+^ and CD8^+^ T cells. Volumetric growth curves, total itIC counts, percent itIC subpopulations (CD3^+^ T cells, DCs, NK cells, macrophages (MΦ) and MDSC-like (MDSC) cells) and intratumoral CD3^+^CD4^+^ (CD4^+^) and CD3^+^CD8^+^ (CD8^+^) T cell counts of SW1 tumors (**a**–**d**, respectively) or of empty vector (EV)- or mFuk-expressing SW1 tumors (**e**–**h**, respectively) in C3H/HeN mice. Red triangle, initiated l-fuc (LF) supplementation. The growth curves show mean ± standard error of the mean (s.e.m.) from groups of mice as follows: *n* = 11 control and *n* = 10 l-fuc-fed mice (**a**), *n* = 4 mice per group (**b**,**d**), *n* = 7 mice per group (**e**), *n* = 3 mice per group (**f**,**h**)(b,d,f,h show mean ± s.e.m.). **i**, Association of melanoma-specific fucosylation and CD3^+^ T cell density (log_2_ scale) in a 40-patient melanoma tissue microarray (med = median fucosylation signal per melanoma cell). **j**, Box plots showing lower melanoma-specific fucosylation in male (*n* = 22) versus female (*n* = 18) patients. The minima and maxima represent the minimum and maximum tumor fucosylation values while the centra represent median values. *P* values shown are two-sided *P* values derived from the Spearman correlation test. **k**, Scatterplots show higher correlation (cor) between melanoma-specific fucosylation and CD3^+^ T cell density (log_2_ scale) in male (Spearman’s *ρ* = 0.43; *P* = 0.036) versus female (Spearman’s *ρ* = 0.25; *P* = 0.3367) patients. The gray bands highlight 95% confidence bands for the prediction line (based on linear regression). Volumetric growth curves for SW1 tumors in PBS (control)-injected (both (control and l-fuc) groups, *n* = 7 mice) (**l**), CD8^+^ T cell- (both groups, *n* = 6 mice) (**m**) or CD4^+^ T cell- (**n**) immunodepleted C3H/HeN mice (both groups, *n* = 7 mice). **o**, Comparison of intratumoral NK, DC, CD8^+^ T and CD4^+^ T cell subpopulations (absolute cell numbers) from tumors (*n* = 4 (control) and *n* = 3 (l-fuc)) (**l**), (for both groups, *n* = 4) (**n**). All error bars represent s.e.m. With the exception of **e**, for which one-way ANOVA was performed, two-sided *t*-tests were performed for all other analyses. Source data

**Extended Data Fig. 1:**
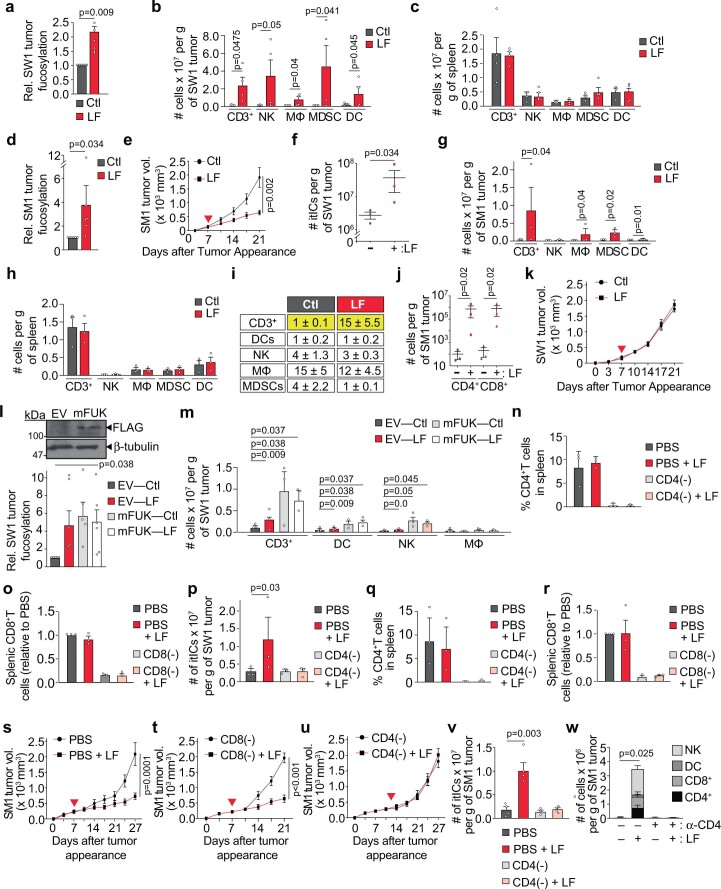
Confirming increased tumor fucosylation and itIC counts, splenic immune cell profiles, and correlations between tumor fucosylation and CD3^+^T cells in female vs. male melanoma patients. Immunofluorescent (IF) staining analysis of SW1 tumor FFPE sections for intratumoral fucosylation **(a)** (n = 2 representative fields from 3 mice per group). Flow cytometric profiling of intratumoral immune cell (itIC) subpopulations (CD3^+^T cells, natural killer cells (NKs), macrophages (MΦ), MDSC-like cells (MDSCs), and dendritic cells (DCs)) in **(b)** SW1 tumors (n = 4 mice per group) and **(c)** spleens (n = 3 mice per group) in control (Ctl)- or L-fuc (LF)-supplemented SW1 tumor-bearing C3H/HeN mice from Fig. 1a. IF staining analysis of SM1 tumor FFPE sections for intratumoral fucosylation **(d)** (n = 2 representative fields from 3 mice per group)**. (e)** Volumetric growth curves (n = 7 and 8 mice for Ctl and LF groups, respectively), **(f)** total itIC counts, **(g)** absolute itIC subpopulations, **(h)** splenic immune cell profiles, **(i)** % itIC subpopulations, **(j)** intratumoral CD4^+^ and CD8^+^T cell counts of SM1 tumors in C57BL6 mice (for **(f-j)**, n = 3 mice per group). **(k)** Volumetric growth curves of SW1 tumors in NSG mice (n = 10 mice per group). **(l)** IB analysis confirming mFUK expression in SW1 cells (*upper*), IF staining analysis of SW1 tumor FFPE sections for intratumoral fucosylation (*lower*) (n = 2 representative fields from 3 mice per group), and **(m)** flow cytometric profiling of indicated immune populations in EV- or mFUK-expressing SW1 tumors from Ctl- or LF-supplemented C3H/HeN mice. **(n)** Flow cytometric profiling of splenic CD4^+^T cells in control (PBS-injected) vs. CD4^+^T cell-depleted (CD4(-)) SW1 tumor-bearing C3H/HeN mice supplemented ± LF. **(o)** IF staining profiling of splenic CD8^+^T cells in control vs. CD8^+^ T cell-depleted (CD8(-)) SW1 tumor-bearing C3H/HeN mice. Flow cytometric profiling of **(p)** total itIC counts in control vs. CD4(-) SW1 tumor-bearing C3H/HeN mice and **(q)** splenic CD4^+^T cells in control vs. CD4(-) SM1 tumor-bearing C57BL6 mice supplemented ± LF. Percentages represent % CD4^+^T of total splenic cells. **(r)** IF profiling of splenic CD8^+^T cells in control vs. CD8(-) SM1 tumor-bearing C57BL6 mice fed ± L-fuc (for **(m-r)**, n = 3 mice per group). Volumetric growth curves for SM1 tumors in **(s)** control (PBS)-injected, **(t)** CD8(-), or **(u)** CD4(-) C57BL6 mice (for **(s-u)**, n = 7 mice per group, except for PBS + LF, which had 8 mice). **(v)** Flow cytometric profiling of total itIC counts in control **(s)** vs. CD4(-) **(u)** mice. **(w)** Comparison of intratumoral NK, DC, CD8^+^T, and CD4^+^T subpopulations from control or CD4(-) (α-CD4) depleted tumors in **(s)** and **(u)** (for **(v and w)**, n = 4 mice per group). *For each mouse model: when tumors reached ~150 mm*^3^*, Ctl- or LF-supplemented water (100* *mM; red triangle* = *initiated supplementation) was provided ad libitum. The tumor growth curves are means* ± *SEM per group. Relative fold-changes in splenic CD8*^*+*^*T cells were determined by (total intrasplenic CD8*^*+*^ *signal area / total intrasplenic DAPI area) as a measure of relative CD8*^*+*^*T cell abundance/spleen*. All error bars represent standard error of the mean. Source data

We initially assessed how l-fuc-induced changes in itICs might contribute to melanoma suppression using a NRAS^G13D^-mutant mouse melanoma (SW1) model^9^. Oral l-fuc administration increased tumor fucosylation (approximately twofold), reduced tumor growth (~50%) and increased total itICs (~10–50-fold) (including CD3^+^ (CD4^+^ and CD8^+^) T, natural killer (NK), macrophage, dendritic cell (DC) and myeloid-derived suppressor (MDSC)-like cell subpopulations, without altering splenic lymphocyte profiles) (Extended Data Fig. 1a, Fig. 1a,b and Extended Data Fig. 1b,c, respectively). Of total itICs, CD4^+^ and CD8^+^ T cells were the most increased subpopulation (approximately doubled) (Fig. 1c,d). Oral l-fuc induced similar changes in tumor fucosylation, growth and itICs (specifically increased CD4^+^ and CD8^+^ T cells) in a BRAF^V600E^-mutant mouse melanoma (SM1) model^12^ (Extended Data Fig. 1d–j, respectively). By contrast, l-fuc did not reduce SW1 tumor growth in immunodeficient mice (Extended Data Fig. 1k), confirming that the presence and activity of itICs are essential for l-fuc-triggered tumor suppression.

We confirmed an essential role for tumor-specific fucosylation by overexpressing murine fucokinase (mFuk) in SW1 melanoma cells to exclusively increase tumor fucosylation. mFuk expression alone suppressed tumor growth and increased total itICs comparably to oral l-fuc administration alone. Again, CD4^+^ and CD8^+^ T cells were the most increased itICs (Extended Data Fig. 1l,m and Fig. 1e–h). These data indicate that melanoma-specific fucosylation is an essential determinant of l-fuc-triggered itIC induction and tumor suppression, regardless of any other physiological host effects that l-fuc may elicit (for example, microbiome or metabolic effects).

Correlations between tumor fucosylation and CD3^+^ T cells in humans were assessed by immunofluorescently analyzing a 40-patient melanoma microarray. Patients with higher-than-median tumor fucosylation levels exhibited significantly increased intratumoral CD3^+^ T cell densities (Fig. 1i). Intriguingly, average melanoma fucosylation levels were lower in male patients (Fig. 1j) but exhibited a stronger association with intratumoral CD3^+^ T cells (Fig. 1k).

These data indicate that melanoma fucosylation substantially shapes the itIC landscape, correlates with increased intratumoral CD3^+^ T cells in mice and humans and can be boosted by oral l-fuc to increase itICs and suppress *BRAF*- and *NRAS*-mutant melanomas.

### l-fuc triggers CD4^±^ T cell-induced itICs and alters CD4^±^ T cell biology

The contribution of CD4^+^ and CD8^+^ T cells to l-fuc-triggered tumor suppression was assessed by immunodepletion in the SW1 model. l-fuc reduced tumor growth by >50% in control and CD8^+^ T cell-depleted mice, whereas this effect was completely abrogated by CD4^+^ T cell depletion (Fig. 1l–n, immunodepletion confirmed by splenic profiling, and Extended Data Fig. 1n,o). Consistent with known roles for CD4^+^ T cells in recruiting and activating tumor-suppressive itICs^13^, CD4^+^ T cell depletion also blocked l-fuc-induced increases in total itICs, including intratumoral NK cells, DCs and CD8^+^ T cells, observed in control mice (Extended Data Fig. 1p and Fig. 1o). Similarly, in the SM1 model, CD4^+^ but not CD8^+^ T cell depletion abrogated l-fuc-triggered tumor suppression and increases in total itICs and itIC subpopulations (immunodepletion confirmed by splenic profiling, Extended Data Fig. 1q–w)).

**Fig. 2:**
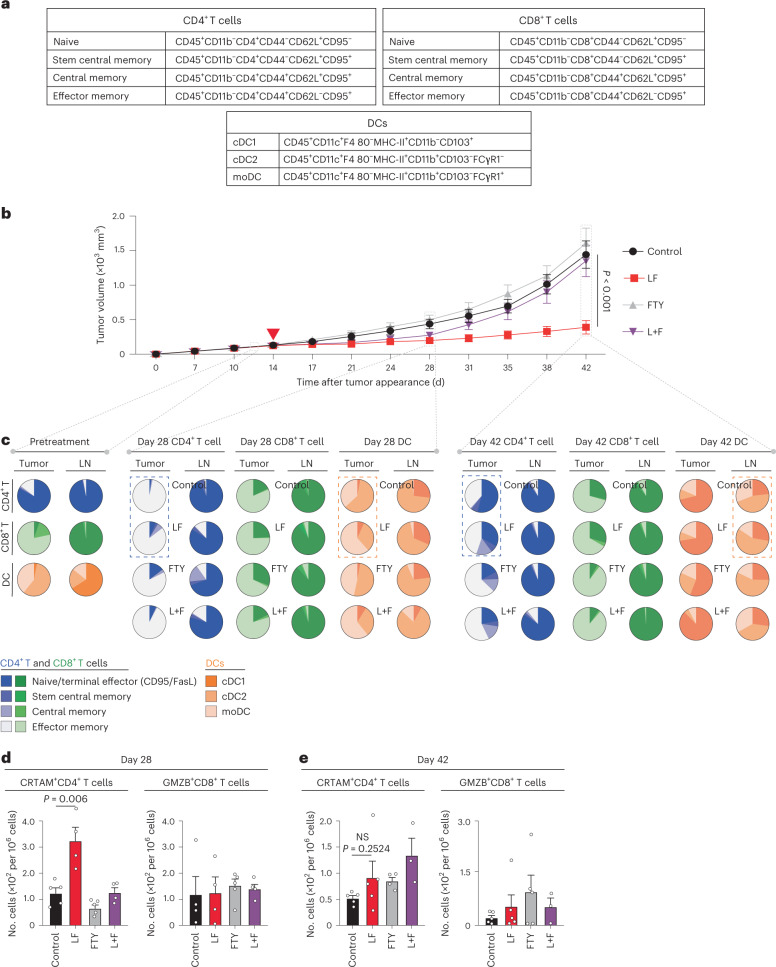
Lymph node egress is necessary for l-fuc-triggered tumor suppression; l-fuc increases intratumoral CD4^+^ T stem and central memory cells. **a**, Immune subpopulation markers use to profile by flow cytometry. **b**, Volumetric growth curves for SW1 tumors in C3H/HeN mice fed without (control, *n* = 3 mice) or with (l-fuc, *n* = 10 mice) l-fuc and treated with FTY720 (control mice were administered FTY720 (FTY) (*n* = 10 mice); l-fuc-supplemented mice were administered FTY720 (L + F) (*n* = 10 mice)). FTY720 was administered at 20 µg per mouse every 2 d starting on day 12, just before the initiation of l-fuc administration. **c**, Pie charts showing ratios of intratumoral or lymph node (LN)-resident CD4^+^ or CD8^+^ T cell subpopulations as well as DC subtypes from mice at days 14, 28 and 42 (each pie chart represents 4–5 mice). Assessment of cytotoxic CD4^+^ T cell populations (CRTAM^+^) and cytotoxic CD8^+^ T cell populations (GrzB^+^) from tumors at day 28 (**d**) (*n* = 5 mice for all groups except l-fuc, where *n* = 4 mice) and day 42 (**e**) (*n* = 5 mice each for control and l-fuc groups, *n* = 4 mice each for FTY and L + F groups). NS, not significant. Corresponding raw flow cytometric data for these charts are shown in Supplementary Table 1. The tumor growth curves and column charts show mean ± s.e.m. per group of mice. Source data

**Extended Data Fig. 2:**
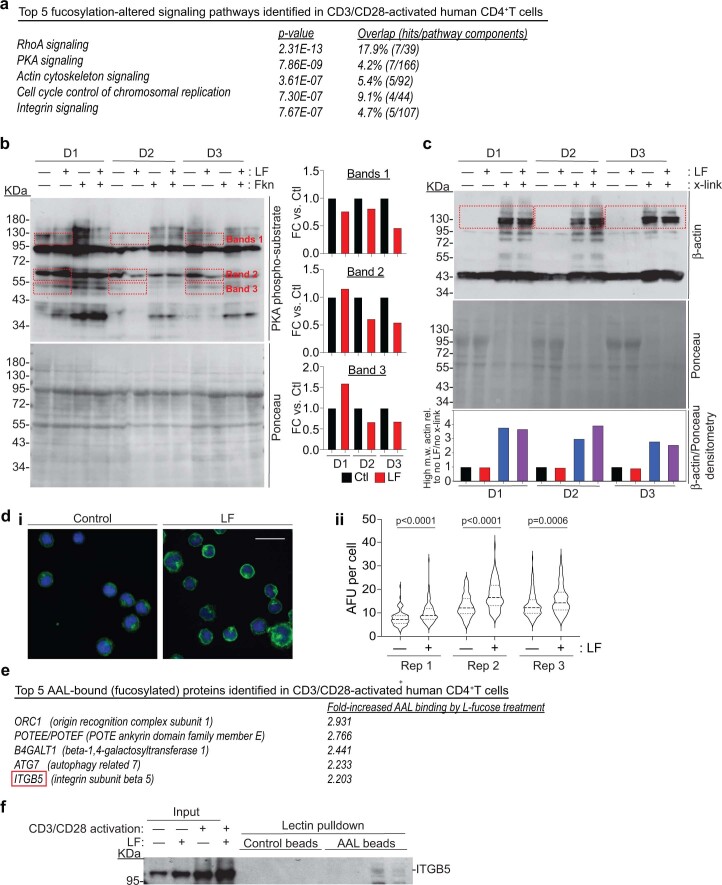
Fucosylation of CD4^+^T cells affects PKA activity and actin polymerization; and the identification of Integrin β5 as a highly fucosylated protein in activated CD4^+^T cells. **(a)** Top 5 pathways in human CD4^+^T cells that are affected by increased fucosylation identified by Ingenuity Pathway Analysis (Qiagen; pathways were identified from phosphoproteomic analyses of CD3/CD28-activated human PBMC-derived CD4^+^T cells treated ± 250 µM L-fucose (LF) isolated from 3 independent healthy human donors (D1, D2, D3)). **(b)** (*left*) Immunoblot of PKA phosphorylated substrates (*top*) and Ponceau staining (*bottom*) of human PBMC-derived, CD3/CD28-activated CD4^+^T cells that were treated ± 250 µM LF (for 72 h) ± 10 µM forskolin (Fkn, a PKA agonist; for 6 h). (*right*) Densitometric quantification of selected bands (red dashed boxes) normalized to Ponceau staining. **(c)** Immunoblot of β-actin (*top*) and Ponceau staining (*middle*) of 3 human donor PBMC-derived, CD3/CD28 activated CD4^+^T cells were treated ± 250 µM LF ± 25 mM DTSP crosslinker (x-link). (*bottom*) Densitometric quantification of high molecular weight β-actin oligomers (red dashed boxes) normalized to Ponceau intensity and then normalized to -LF, -x-link samples. **(d) (i)** Coverslip-grown Jurkat cells treated ± 250 µM LF (for 72 h), fixed, and stained with DAPI (*blue*) and phalloidin-488 (*green*) for actin cytoskeleton. Scale bar: 25 µm. **(ii)** Integrated actin signal densities per cell from 3 biological replicates of Jurkat cells grown/treated as in **(i)** were measured using ImageJ v1.53a (NIH) and plotted as shown using GraphPad Prism v8. **(e)** Top 5 AAL-bound (fucosylated) proteins in human PBMC-derived, CD3/CD28-activated CD4^+^T cells from **(a)** that were identified by Ingenuity Pathway Analysis (Qiagen). **(f)** Of the 5 top hits, we were only able to validate fucosylation of Integrin β5 by LPD analysis of human PBMC-derived, CD3/CD28-activated CD4^+^T cells. Source data

Phosphoproteomic and fucosylated proteomic analyses revealed that l-fuc mechanistically regulates CD4^+^ T cell biology by significantly altering protein kinase A (PKA) and (to a lesser extent) actin signaling, potentially via integrin B5, an upstream regulator of both of these pathways^14^ that we discovered to be one of five proteins most highly bound to *Aleuria aurantia* lectin (AAL) (and likely fucosylated) in human peripheral blood monocyte (PBMC)-derived, CD3–CD28-activated CD4^+^ T cells, as well as Jurkat cells treated with l-fuc (Extended Data Fig. 2a–f). The fact that integrin, PKA and actin signaling have been reported to mediate T cell activation, motility and immune synapse formation^15,16^ suggests that l-fuc promotes T cell trafficking to the tumor, a notion confirmed using an SW1 melanoma C3H mouse model treated with or without FTY720 (an inhibitor of lymph node egress). Inhibition of lymph node egress completely abrogated l-fuc-triggered tumor suppression (Fig. 2a,b). Strikingly, l-fuc-triggered tumor suppression was associated with increases in intratumoral CD4^+^ T central and effector memory subpopulations that were abrogated by FTY720 (Fig. 2a,c (blue dashed boxes) and Supplementary Table 1), consistent with the role that PKA plays in regulating memory phenotype in T cells^17^. Intriguingly, oral l-fuc induced significant, albeit transient, increases in intratumoral monocyte-derived DCs (moDCs) and lymph node conventional DC2 (cDC2) cells, which can promote memory CD4^+^ T cell phenotypes and cross-talk with CD4^+^ T cells to mediate tumor suppression, respectively^18–20^ (Fig. 2a,c (orange dashed boxes) and Supplementary Table 1). Finally, l-fuc also transiently but significantly increased cytotoxic CD4^+^ T cells at the midpoint (day 28) of the experiment (Fig. 2d,e).

These data confirmed that CD4^+^ T cells play a key role in induction of itICs and suppression of melanomas by l-fuc, suggesting that l-fuc triggers key changes in CD4^+^ T cell signaling and biology at the tumor and lymph node levels that are important for tumor suppression. Importantly, the fact that mFuk expression alone in melanoma cells resulted in smaller tumors with increased itICs (Fig. 1e–h) suggests that melanoma-specific fucosylated protein(s) can also promote anti-tumor immunity, although the mechanism was unclear.

### Fucosylated HLA-DRB1 induces itICs and melanoma suppression

**Fig. 3:**
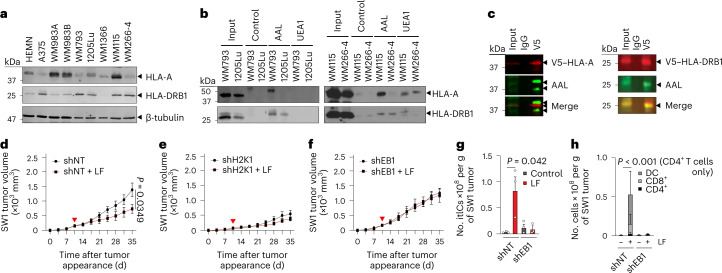
HLA-DRB1 is expressed, fucosylated and required for l-fuc-triggered melanoma suppression and increased itIC abundance. **a**, IB analysis of HLA-A and HLA-DRB1 levels in primary human melanocytes (HEMN) or the indicated human melanoma cell lines. **b**, LPD and IB analysis of patient-matched primary and metastatic cell line pairs WM793 and 1205Lu (left) and WM115 and WM266-4 (right) for HLA-A and HLA-DRB1. **c**, V5-immunoprecipitation and IB analyses of WM793 cells expressing (left) V5-tagged HLA-A or (right*)* V5-tagged HLA-DRB1. Volumetric growth curves for non-targeting control short hairpin RNA (shRNA) (shNT)- (**d**), H2K1-targeting shRNA (shH2K1)- (**e**) or H2EB1-targeting shRNA (shEB1)- (**f**) expressing SW1 tumors in C3H/HeN mice (*n* = 8 mice for each shNT group, *n* = 6 for each shH2K1 group and *n* = 6 and 7 for shEB1 and shEB1 with l-fuc groups, respectively). Flow cytometric comparison of total itIC counts (**g**) or the indicated subpopulations (**h**) from shNT- or shEB1-expressing tumors in **d**,**f**. *n* = 3 mice per group. For **d**–**f**, the red triangle indicates initiated l-fuc supplementation; growth curves and column charts show mean ± s.e.m. from each mouse group. In **a**–**c**, representative images are shown for *n* = 3 independent biological replicate experiments. Source data

**Extended Data Fig. 3:**
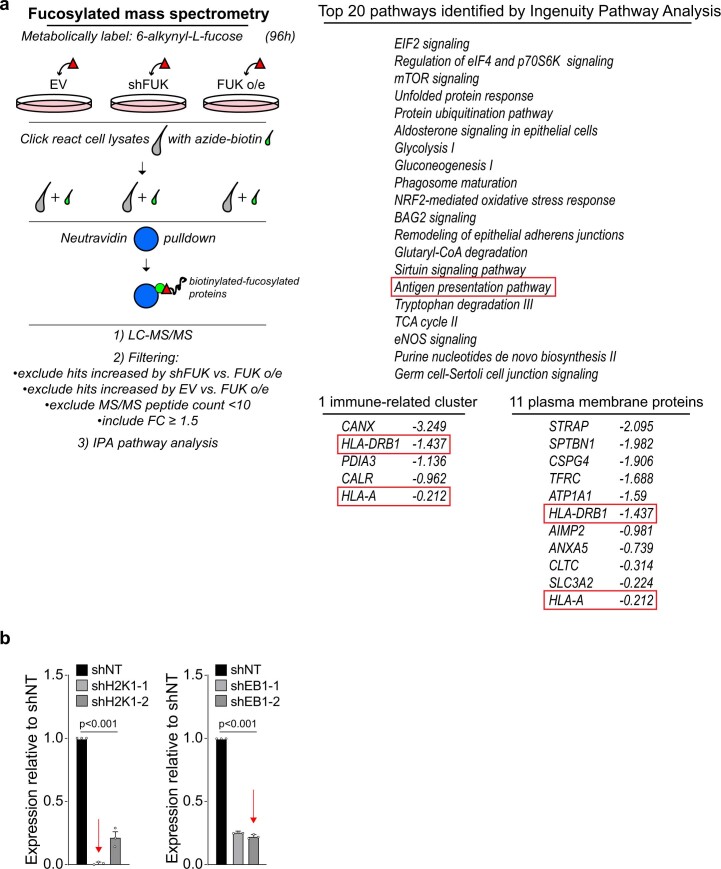
Fucosylated mass spectrometric analysis and knockdown efficiency of H2K1 and H2EB1. **(a)** (*left*) Schematic for proteomic analysis of fucosylated proteins in human WM793 melanoma cells using pLenti-GFP empty vector (EV)-, pLenti-FUK-GFP (o/e)-, or shFUK-expressing WM793 cells from^8^. Click chemistry biotinylated-fucosylated proteins that were pulled down using Neutravidin beads from the 6-alkynyl-L-fucose-labeled cells were subjected to LC-MS/MS, and hits were subjected to the indicated filtering scheme followed by Ingenuity Pathway Analysis (Qiagen). (*right*) Top 20 pathways, plasma membrane- and immune-related proteins identified by Ingenuity Pathway Analysis (Qiagen) to be significantly altered by fucosylation. **(b)** qRT-PCR analysis confirming knockdown of H2K1 (shH2K1; *left*) or H2EB1 (shEB1; *right*) using 2 shRNAs per target compared to control non-targeting (shNT) shRNA. n = 3 biologically independent experiments. Red arrows indicate the specific shRNA clones used in functional experiments in the remainder of the study. All error bars represent standard error of the mean. Source data

To identify melanoma proteins that contributed to fucosylation-triggered, CD4^+^ T cell-mediated melanoma suppression, we subjected fucosylated proteins from human melanoma cells to liquid chromatography–mass spectrometric (LC–MS/MS) analysis followed by Ingenuity Pathway Analysis^21^ (Extended Data Fig. 3a, left). These analyses identified ‘Antigen presentation pathway’ as the only immune-related pathway, in which the MHC-I and MHC-II proteins HLA-A and HLA-DRB1, respectively, were identified as the only antigen-presentation and plasma membrane proteins with T cell-modulating functions^22^ (Extended Data Fig. 3a, right). We confirmed their expression in human melanocytes and melanoma cells by immunoblot (IB) analysis (Fig. 3a). Furthermore, lectin pulldown (LPD) using AAL and *Ulex europaeus* agglutinin I (UEA1) lectins, which bind to common core and terminal fucosylated glycans, respectively^23–28^, revealed association of both proteins with AAL (and to a lesser extent, UEA1), suggesting *N*′-linked core glycosylation–fucosylation (Fig. 3b). Finally, immunoprecipitation and IB analysis of V5-tagged HLA-A or HLA-DRB1 revealed direct recognition of HLA-DRB1 by AAL, indicating that a fraction of total HLA-DRB1 but not HLA-A is directly fucosylated in melanoma (Fig. 3c).

To determine contributions of HLA-A or HLA-DRB1 to fucosylation-triggered anti-tumor immunity, we knocked down their C3H/HeN mouse orthologs H2K1 or EB1 (ref. ^29^), respectively, in SW1 tumors (Extended Data Fig. 3b) and assessed growth and itICs in vivo. Whereas l-fuc impaired control tumor growth, H2K1 knockdown suppressed tumor growth regardless of l-fuc (Fig. 3d,e), potentially reflecting tumor-protective, immunosuppressive roles of MHC-I proteins^30,31^. Notably, EB1 knockdown completely abolished l-fuc-triggered tumor suppression and induction of total itICs, including DC, CD8^+^ and CD4^+^ T cell subpopulations (Fig. 3f–h), similar to the effects elicited by CD4^+^ T cell depletion (Fig. 1l–o).

Consistent with roles of HLA-DRB1 in CD4^+^ T cell activation^32–34^, our findings demonstrate that HLA-DRB1 is expressed and fucosylated in melanoma and required for l-fuc-triggered CD4^+^ T cell-mediated itIC induction and melanoma suppression.

### Fucosylation regulates HLA-DRB1 localization and immunological effects

**Fig. 4:**
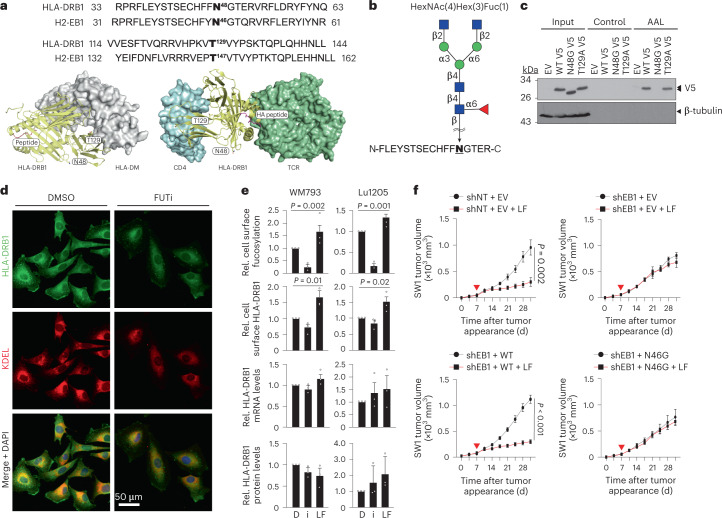
*N*-linked fucosylation of HLA-DRB1 at N48 regulates its cell surface localization and is required for tumor suppression and increased itIC abundance. **a**, Top, amino acid sequence alignments showing conservation of predicted *N*- and *O*-linked fucosylation sites in human HLA-DRB1 (N48 and T129) and mouse H2EB1 (N46 and T147). Structural modeling of the HLA-DRB1–HLA-DM (bottom left) and CD4–HLA-DRB1–TCR (bottom right) complexes. Potential glycosylation sites, N48 and T129, of the HLA-DR1 β-chain are shown as sticks. CD4 (cyan), HLA-DRB1 (yellow), antigen peptide (magenta) and TCR (green) (bottom right). **b**, The HLA-DRB1 peptide fragment was identified by nano-LC–MS to be fucosylated on N48, with the predicted HexNAc(4)Hex(3)Fuc(1) glycan structure shown above. **c**, LPD and IB analyses of EV and V5-tagged WT HLA-DRB1 (WT)-, HLA-DRB1^N48G^ (N48G)- and HLA-DRB1^T129A^ (T129A)-expressing WM793 cells. **d**, DMSO- or FUTi-treated WM793 cells immunofluorescently stained for endogenous HLA-DRB1 (green), KDEL (ER marker; red) and 4,6-diamidino-2-phenylindole (DAPI) (blue) (×20 magnification). **e**, Flow cytometric analysis for relative (rel.) cell surface fucosylation (top) and cell surface HLA-DRB1 (top middle), quantitative PCR with reverse transcription (RT–qPCR) analysis of relative HLA-DRB1 mRNA levels (bottom middle) and IB analysis of HLA-DRB1 protein levels (bottom) in WM793 and 1205Lu cells treated with DMSO (D), 250 µM FUTi (i) or 250 µM l-fuc. *n* = 3 biologically independent experiments. **f**, Volumetric growth curves for shNT and EV (control SW1 tumors) (top left) or shEB1 tumors reconstituted with EV (top right), EB1^WT^ (bottom left) or EB1^N46G^ (bottom right) in C3H/HeN mice. Control (gray) or l-fuc-supplemented water (red, 100 mM; red triangle, initiated supplementation) was provided ad libitum. Tumor growth curves show mean ± s.e.m. per mouse group. *n* = 7 mice per group except the shEB1 and EV group, which had six mice. For **c**,**d**, representative images are shown for *n* = 3 independent biological replicate experiments. Source data

We reasoned that determining how HLA-DRB1 is regulated by fucosylation would provide important insight into its crucial role in l-fuc-triggered anti-tumor immunity. Using NetNGlyc version 1 and NetOGlyc version 4 (https://services.healthtech.dtu.dk)^35^, we predicted *N*- and *O*-linked glycosylation sites at Asn48 (N48) and Thr129 (T129), respectively, which are conserved sites within constant regions of human and mouse HLA-DRB1 (Fig. 4a, top)^29,36^. Importantly, EB1 exhibits ~80% sequence homology with HLA-DRB1 and contains the conserved glycosylation–fucosylation site at N46 (ref. ^29^). Modeling of HLA-DRB1 interactions with prominent binding partners HLA-DM or CD4–TCR suggests that fucosylation of neither site affects interaction interfaces or peptide loading or presentation (Fig. 4a, bottom).

**Extended Data Fig. 4:**
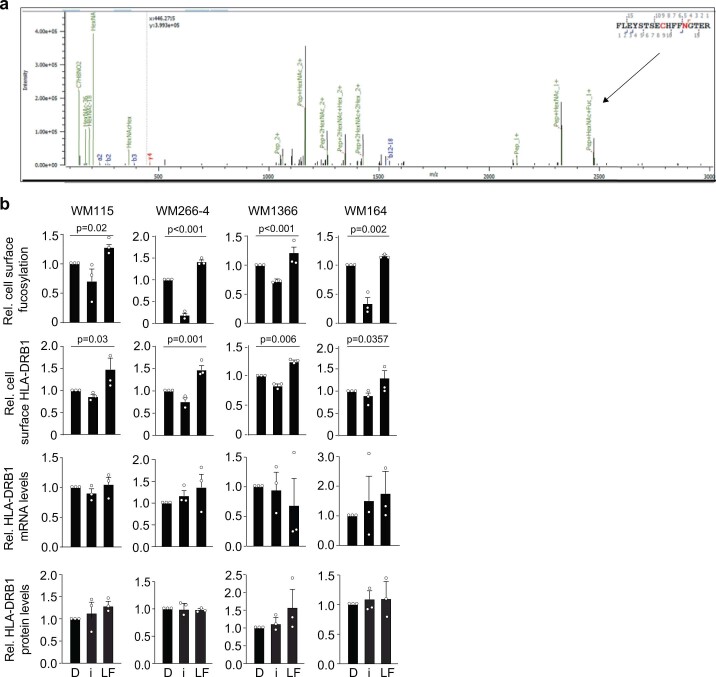
nano-LC/MS spectral identification of fucosylated HLA-DRB1 peptide; and the effects of modulating fucosylation on HLA-DRB1 localization, total protein, and mRNA levels. **(a)** nano-LC/MS/MS spectra showing fucosylated HLA-DRB1 peptide (*arrow*). **(b)** Flow cytometric analysis for relative cell surface fucosylation (*upper*) and cell surface HLA-DRB1 levels (*upper middle*), qRT-PCR analysis of relative HLA-DRB1 mRNA levels (*lower middle*), and IB analysis of HLA-DRB1 protein levels (*lower*) in indicated melanoma cell lines treated with DMSO (D), 250 µM FUTi (i), or 250µM L-fuc (LF). n = 3 biologically independent experiments. All error bars represent standard error of the mean. Source data

Nano-LC–MS/MS analysis of HLA-DRB1 immunoprecipitated from WM793 cells identified the fragment FLEYSTSECHFFNGTER as glycosylated–fucosylated at N48 with the predicted glycan HexNAc(4)Hex(3)Fuc(1) (Fig. 4b and Extended Data Fig. 4a). We mutated N48 or T129 to Gly or Ala, respectively, to abolish and verify fucosylation^37–39^. Unlike wild-type (WT) or the T129A ‘glycofucomutant’ HLA-DRB1, the HLA-DRB1^N48G^ glycofucomutant did not bind to AAL in LPD assays (Fig. 4c), confirming fucosylation at N48 on an N-linked glycan.

**Extended Data Fig. 5:**
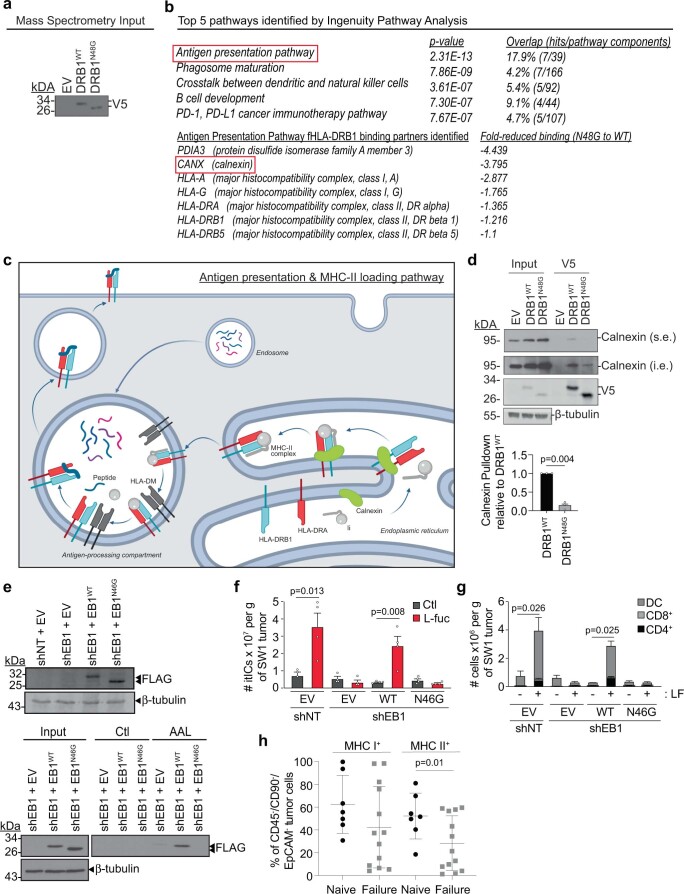
Proteomic analysis reveal fuco/glycosylation of HLA-DRB1 decreases binding to calnexin; knockdown/reconstitution and fucosylation of EB1 WT and N46G and its effects on itICs *in vivo*; loss of MHCII is associated with anti-PD1 failure in melanoma patients. **(a)** IB analysis of 5% input of V5 IP of tagged EV, HLA-DRB1^WT^, and HLA-DRB1^N48G^ mutant in WM793 melanoma cells. **(b)** (*top*) Top 5 pathways that are affected by HLA-DRB1 fuco/glycosylation identified by Ingenuity Pathway Analysis (Qiagen). (*bottom*) Individual proteins in the Antigen Presentation Pathway identified in the screen. **(c)** Schematic of proteins identified in the Antigen Presentation & MHC-II Loading Pathway. The schematic was created using BioRender.com. **(d)** (top) IP of EV, HLA-DRB1^WT^, and HLA-DRB1^N48G^ and IB analysis of calnexin (short exposure (s.e.), calnexin (intermediate exposure (that is), V5, and β-tubulin. (bottom) Quantification of calnexin band intensity to V5 intensity in V5 IP lanes (relative to HLA-DRB^WT^; (n = 3 biologically independent experiments). **(e)** (*upper*) IB analysis of non-targeting shRNA + empty vector (shNT + EV) or shEB1-expressing cells (*from* Extended Data Fig. 3b) reconstituted with FLAG-EV (shEB1 + EV), FLAG-EB1^WT^ (shEB1 + EB1^WT^), or FLAG-EB1^N46G^ (shEB1 + EB1^N46G^). (*lower*) LPD and IB analysis of indicated cells from (*upper*). Shown are representative images from 3 biologically independent experiments. **(f)** Total itIC counts and **(g)** indicated immune subpopulations in the shNT or shEB1 knockdown/EB1^WT^- or EB1^N46G^-reconstituted SW1 tumors of the Ctl- or LF-supplemented C3H/HeN mice. n = 4 mice per group. **(h)** % of total CD45^−^/CD90^−^/EpCAM^−^ tumor cells exhibiting positive pan MHC-I (*left*) or pan MHC-II (*lower*) staining from either anti-PD1 naïve patients (black squares; n = 7) or patients who failed anti-PD1 (*grey squares*; n = 13). All error bars represent standard error of the mean. Source data

To determine how fucosylation might regulate HLA-DRB1, we assessed its subcellular localization in WM793 cells that were pharmacologically modulated for fucosylation by treatment with 2F-peracetyl-fucose (a FUT inhibitor (FUTi)^40^) versus vehicle (dimethylsulfoxide, DMSO; control). Cells treated with FUTi exhibited dimmer, more central, endoplasmic reticulum (ER)-colocalization of HLA-DRB1 than vehicle-treated cells, suggesting less accumulation at the cell surface (Fig. 4d). Furthermore, flow cytometry revealed that cell surface fucosylation and HLA-DRB1 both decreased or increased after FUTi or l-fuc treatments, respectively, whereas mRNA and protein levels remained unchanged; thus fucosylation promotes cell surface localization of HLA-DRB1 (Fig. 4e and Extended Data Fig. 4b). Finally, global proteomic profiling to identify interactors that might mediate fucosylation-regulated cell surface localization of HLA-DRB1 revealed that N48 glycosylation–fucosylation promotes binding to calnexin, which has been reported to mediate maturation and trafficking of MHC-II complexes to the surface^41^ (Extended Data Fig. 5a–d).

To assess how HLA-DRB1 glycosylation–fucosylation contributes to tumor suppression and itICs, we compared control- or EB1-knocked-down SW1 tumors reconstituted with WT or glycofucomutant (N46G) EB1 (confirmation of knockdown reconstitution and fucosylation by IB and LPD, respectively, in Extended Data Fig. 5e). Abrogation of l-fuc-induced itIC and tumor growth suppression by EB1 knockdown was rescued by reconstitution with only WT but not glycofucomutant EB1, demonstrating that glycosylation–fucosylation of EB1 or HLA-DRB1 is essential for l-fuc-triggered itIC induction and melanoma suppression (Fig. 4f and Extended Data Fig. 5f,g). This is consistent with our finding that loss of glycosylation–fucosylation of HLA-DRB1 or EB1 abrogates its cell surface localization and impairs its ability to induce anti-tumor immunity. Thus, despite the other fucosylated proteins identified in melanoma cells (Extended Data Fig. 3), these data confirm that the N48 glycosylation–fucosylation of HLA-DRB1 is a key regulator of anti-melanoma immunity and tumor suppression. Despite other potential host physiological effects of dietary l-fuc (for example, microbiome, metabolome, etc.), these data confirm that l-fuc-induced itIC increases and melanoma suppression are critically mediated by melanoma-intrinsic expression and fucosylation of HLA-DRB1, which promotes its cell surface accumulation to trigger CD4^+^ T cell-mediated anti-tumor immune responses.

### Oral l-fuc augments anti-PD1-mediated melanoma suppression

**Fig. 5:**
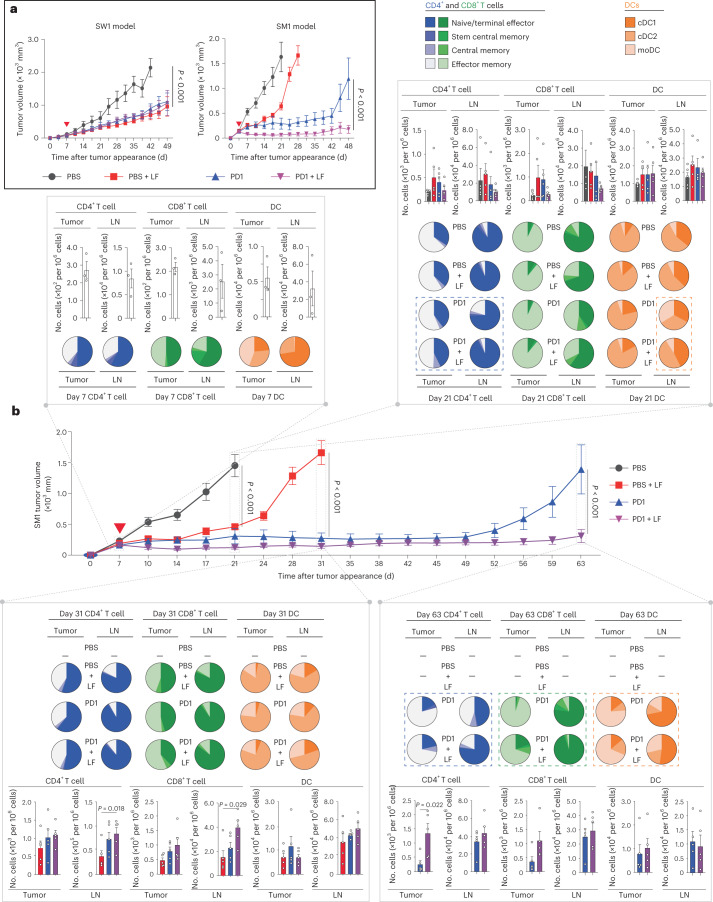
Administration of combination l-fuc and anti-PD1 therapy suppresses tumors and increases intratumoral CD4^+^ T central and effector memory cells. **a**, Volumetric growth curves for SW1 tumors in C3H/HeN mice (left) and SM1 tumors in C57BL/6 mice (right) fed with or without l-fuc and treated with PBS (control) or anti-PD1 therapy (concurrent initiation of l-fuc with or without anti-PD1 therapy (red triangle)). The tumor growth curves show mean ± s.e.m. per mouse group. For each group, *n* = 7 mice except PBS with l-fuc and anti-PD1 therapy with l-fuc groups, which each have eight mice. **b**, Volumetric growth curves for SM1 tumors in C57BL/6 mice fed with or without l-fuc and treated with PBS (control) or anti-PD1 therapy (PD1) (concurrent initiation of l-fuc with or without anti-PD1 therapy (red triangle)). The tumor growth curves show mean ± s.e.m. from ≥7 mice per group. At day 7 (before administration of l-fuc or anti-PD1 therapy, *n* = 3 mice), day 21 (endpoint for tumors of control-treated mice, *n* = 5 mice per group), day 31 (endpoint for tumors of l-fuc-treated mice, *n* = 5 mice per group) and day 63 (endpoint for tumors of anti-PD1-treated mice, *n* = 5 mice per group), the primary tumors (tumor) and draining lymph nodes of 4–5 mice per treatment group were analyzed by flow cytometry for intratumor levels of CD4^+^ and CD8^+^ T subpopulations (naive or terminal; stem central, central or effector memory) and DC subpopulations (cDC1, cDC2 and moDC) as in Fig. 2. Proportions of CD4^+^, CD8^+^ and DC subpopulations in each organ at each time point are represented by the color-coded pie charts (each pie chart represents 4–5 mice). Absolute numbers of the subpopulations per 10^6^ cells of tumor or tissue homogenate at each time point are represented in the color-coded column charts. Corresponding raw flow cytometric data for these charts are shown in Supplementary Table 2. Column charts show mean ± s.e.m. from each mouse group. Source data

**Extended Data Fig. 6:**
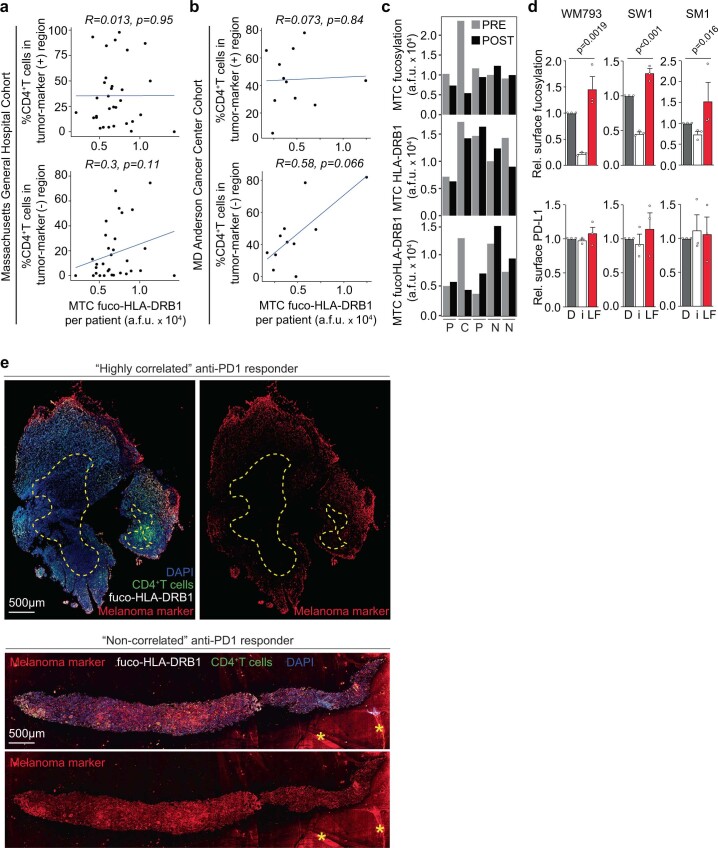
Spatial and pre-/post-anti-PD1 trends in fucosylation, total-/fuco-HLA-DRB1 & CD4^+^T cells in patient specimens, lack of effect of fucosylation on cell surface presence of melanoma PD-L1, & examples of stromal content discrepancy among patient biopsies. **(a)** WM793 (*left*), SW1 (*middle*), and SM1 (*right*) cells were treated with DMSO (D) or 250 µM FUTi (i) or L-fuc (LF) and subjected to flow cytometric assessment of changes in cell surface levels of fucosylation (*upper*) and PD-L1 (*lower*). n = 3 biologically independent experiments. All error bars represent standard error of the mean. Association of mean tumor cellular (MTC) fucosylated HLA-DRB1 with % CD4^+^T cells either inside (tumor marker (+); *upper*) or outside (tumor border/periphery; tumor marker (-); *lower*) melanoma tumors in patients from **(b)** Massachusetts General Hospital or **(c)** MD Anderson Cancer Center (MDACC). **(d)** A ‘Highly correlated’ anti-PD1 responder biopsy with high fuco-HLA-DRB1 and CD4^+^T cell (*upper*) vs. a ‘non-correlated’ responder biopsy with low fuco-HLA-DRB1 and CD4^+^T cells were stained for indicated markers. Yellow dashed lines represent the tumor:stromal interface surrounding melanoma marker-negative stroma in the highly correlated responder that is absent in the non-correlated responder. Yellow asterisks indicate non-nucleated non-specific staining on the non-correlated responder slide. **(e)** Mean tumor cellular (MTC) total fucosylation (*upper*), total HLA-DRB1 (*middle*), or fucosylated HLA-DRB1 (*lower*) levels in MDACC patient-matched pre-/post-anti-PD1 tumor specimens. C/P/N = Complete/partial/non-responder, respectively. P-values shown are two-sided P-values derived from the Spearman correlation test. Source data

Expression of MHC-II reportedly correlates with increased anti-PD1 efficacy^42,43^. Indeed, patients who failed anti-PD1 therapy exhibited relative >45% reduced cell surface MHC-II but not MHC-I (Extended Data Fig. 5h). As anti-PD1 efficacy can be limited by itIC abundance^44^, particularly of CD4^+^ T and memory CD4^+^ T cells^10,45–49^, we tested whether the ability to increase CD4^+^ T cell-mediated itIC induction and tumor suppression using oral l-fuc could be leveraged to augment anti-PD1 efficacy. In the SW1 model, oral l-fuc suppressed tumors as much as anti-PD1 but did not enhance efficacy of anti-PD1 therapy (~50–60%; Fig. 5a, left). By contrast, in the SM1 model, l-fuc was less tumor suppressive than anti-PD1 therapy alone but rather augmented durable suppression in combination with anti-PD1 therapy (Fig. 5a, right). Importantly, we also found that l-fuc does not alter cell surface levels of programmed cell death ligand 1 (PD-L1) in mouse or human melanoma cells (Extended Data Fig. 6a), suggesting that the l-fuc-associated tumor suppression in these models is attributed to determinants beyond the PD1–PD-L1 axis.

To clarify how the combination of l-fuc and anti-PD1 therapy enhanced suppression, we characterized immune cell profiles in the tumors and lymph nodes of SM1 tumor-bearing mice over a time course of treatment with l-fuc, with or without anti-PD1 therapy. Administration of l-fuc (1) alone increased intratumoral CD4^+^ T central and effector memory cells, an effect that was increased when combined with anti-PD1 therapy (Fig. 5b (blue dashed boxes) and Supplementary Table 2) and (2) initially expanded intratumoral cDC2 cells, followed by later expansion of cDC2 cells and moDCs in the lymph nodes when combined with anti-PD1 therapy (Fig. 5b (orange dashed boxes) and Supplementary Table 2). In addition to expanding the absolute numbers of intratumoral CD4^+^ and CD8^+^ T cells at the endpoint (day 63), the combination of l-fuc and anti-PD1 therapy increased the relative percentage of intratumoral CD8^+^ T central memory cells (Fig. 5b (green dashed box) and Supplementary Table 2). Thus, l-fuc can suppress some melanomas as effectively as anti-PD1 therapy, whereas, in others, it can enhance efficacy, which is associated with increased intratumoral CD4^+^ T central and effector memory subpopulations and lymph node cDC2 and moDC populations, consistent with the effects of l-fuc observed in Fig. 2.

### Clinical implications of tumor fucosylation and fucosylated HLA-DRB1

**Fig. 6:**
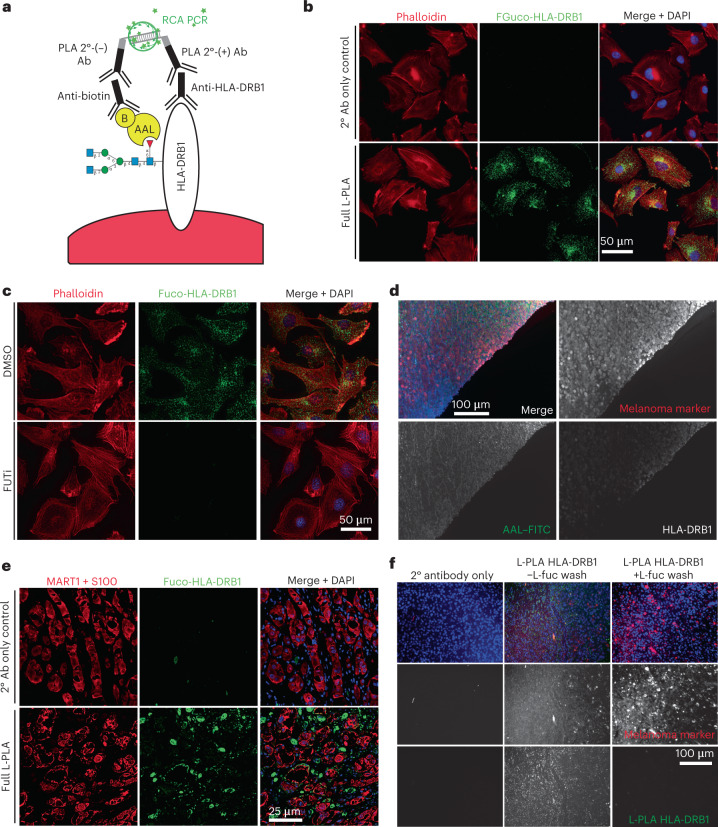
Immunofluorescent visualization of fucosylated HLA-DRB1: development of the lectin-mediated proximity ligation technique. **a**, Schematic of L-PLA using fucosylated HLA-DRB1 (fuco-HLA-DRB1) as an example. We stained for (1) HLA-DRB1 using anti-HLA-DRB1 antibody followed by oligonucleotide-conjugated PLA secondary antibody (2° Ab) (+) and (2) fucosylated glycan using biotinylated (‘B’) AAL lectin followed by anti-biotin antibody followed by oligonucleotide-conjugated PLA secondary (−). Ligated PLA oligonucleotides were subjected to rolling circle amplification PCR (RCA PCR), giving rise to fluorescent punctae. **b**, Representative images of secondary antibody-only control (top) or full L-PLA (bottom) staining of endogenous, fucosylated HLA-DRB1 (green) performed on coverslip-grown WM793 cells (with phalloidin (red) and DAPI (blue) co-stains). **c**, To further demonstrate that fucosylated HLA-DRB1 L-PLA staining is fucosylation species-specific, we performed L-PLA of endogenous, fucosylated HLA-DRB1 (green) on WM793 cells treated with DMSO or FUTi (phalloidin (red) and DAPI (blue) co-stains). **d**, To demonstrate specificity of individual L-PLA primary antibodies, FFPE melanoma tissue was stained for a melanoma marker (MART1 and S100 cocktail; red), AAL–FITC (green), HLA-DRB1 (white) and DAPI (blue). **e**, Representative images of secondary antibody-only control (top) or full L-PLA (bottom) staining of endogenous, fucosylated HLA-DRB1 (green) performed on human melanoma specimens (with MART1 and S100 (red) and DAPI (blue) co-stains). **f**, FFPE melanoma tissues were subjected to L-PLA HLA-DRB1 staining with or without washing with 500 mM l-fuc and subsequent staining with MART1 and S100 (red) and DAPI (blue). Total loss of fucosylated HLA-DRB1 (green) signal in tissue washed with l-fuc confirms the fucose-specificity of L-PLA for fucosylated HLA-DRB1. Single melanoma marker and fucosylated HLA-DRB1 channels are shown in white for clear visualization. For **b**–**f**, representative images are shown for *n* = 3 independent biological replicate experiments. Source data

Given the potent enhancement of anti-PD1 efficacy by oral l-fuc administration in mice, we investigated whether tumor fucosylation or total/fucosylated HLA-DRB1 might correlate at all with responsiveness to anti-PD1 therapy in human patient biopsies, as the identification of preliminary correlations might support their subsequent development into predictive biomarkers for anti-PD1 responsiveness. To this end, we devised a new technique: we modified the conventional proximity ligation assay (PLA)^50^ to facilitate immunofluorescent visualization of fucosylated HLA-DRB1 by applying anti-HLA-DRB1 antibody together with biotinylated AAL, which has previously been successfully used to stain tissues specifically for core-fucosylated glycans^51^ (Fig. 6a). This technique, lectin-mediated PLA (L-PLA), revealed cytoplasmic and/or membranous localization of endogenous fucosylated HLA-DRB1 in melanoma cells (Fig. 6b) that is lost upon FUTi treatment (Fig. 6c), confirming l-fuc-stimulated cell surface localization of HLA-DRB1 (Fig. 4d,e and Extended Data Fig. 4b). The cytoplasmic and/or ’vesicular-appearing’ staining is consistent with HLA-DRB1 that was fucosylated in the endoplasmic reticulum (ER)–Golgi and is en route to the surface via the secretory pathway. In applying this technique further to formalin-fixed, paraffin-embedded (FFPE) melanoma tissue specimens, we observed similar staining patterns for fucosylated HLA-DRB1 (Fig. 6d,e), which were completely abolished by washing the tissue with l-fuc, confirming specificity for fucosylated HLA-DRB1 (Fig. 6f).

**Fig. 7:**
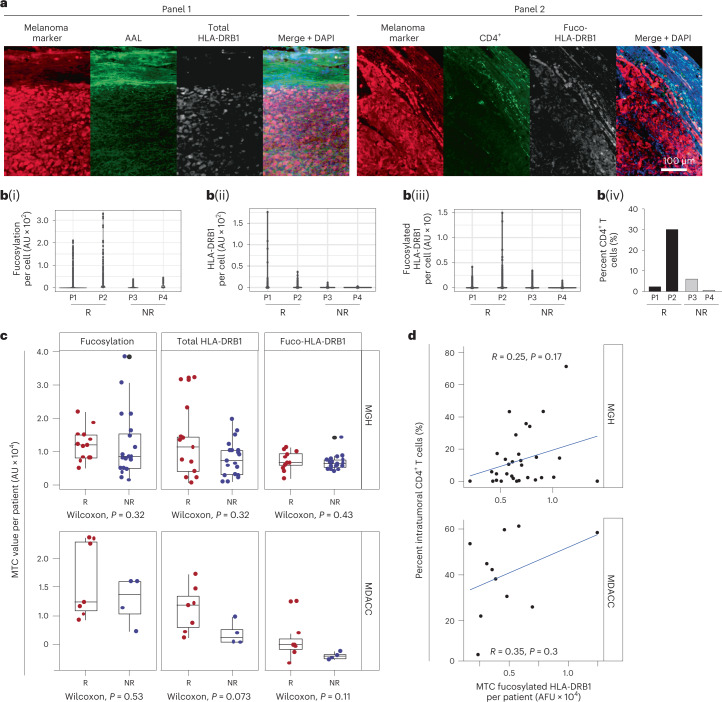
Clinical implications of melanoma fucosylation and fucosylated HLA-DRB1 for anti-PD1 therapy in melanoma. **a**, Representative images of one anti-PD1-treated Moffitt patient tumor subjected to immunofluorescent staining for the two indicated panels of markers. **b**, Dot plots showing single-cell distribution of total fucosylation (AAL) (i), total (ii) and fucosylated (iii) HLA-DRB1 staining intensities per melanoma cell and percent CD4^+^ T cells (of total cells) within tumors of two responder (R; patients (P)1 and 2) and two non-responder (NR; patients 3 and 4) Moffitt patients (iv). AU, arbitrary units. **c**, Box plots showing mean tumor cellular (MTC; means derived from single tumor cell intensities) fucosylation (left), total (center) and fucosylated (right; fuco-HLA-DRB1) HLA-DRB1 staining intensities of anti-PD1 responder (red dots) and non-responder (blue dots) patients from MGH (*n* = 32) (top) or the MDACC (*n* = 11) (bottom). In all box plots, the middle line indicates the median, the lower and upper hinges represent the first and third quartiles and whiskers (from the hinges) extend to 1.5× the interquartile range (IQR). **d**, Percent intratumoral CD4^+^ T cells (of total cells) plotted against corresponding average MTC fucosylated HLA-DRB1 values for each patient in the MGH (top) and MDACC (bottom) cohorts. *P* values shown are two-sided *P* values derived from the Spearman correlation test. Source data

To assess correlations of (1) tumor-specific fucosylation and total/fucosylated HLA-DRB1 of individual tumor cells and (2) intratumoral numbers CD4^+^ T cells with responder status to single-agent anti-PD1 therapy, we implemented L-PLA on primary melanoma biopsies from two distinct responder and two non-responder patients followed by single-cell segmented signal quantitation (Fig. 7a,b). Tumors of responders clearly contained tumor cell populations with high levels of fucosylation and total HLA-DRB1 as compared to non-responders (Fig. 7b(i,ii)). Although the tumor of only one of two responders contained melanoma cells with increased levels of fucosylated HLA-DRB1 compared with those of the non-responders (Fig. 7b(iii)), this trend mirrored that of intratumoral CD4^+^ T cell counts (Fig. 7b(iv)), consistent with the role for fucosylated HLA-DRB1 in CD4^+^ T cell-mediated tumor suppression.

We assessed potential associations between tumor fucosylation, total/fucosylated HLA-DRB1, CD4^+^ T cells and responder status in expanded cohorts of patients with melanoma treated with anti-PD1 therapy. Levels of tumor fucosylation and total and fucosylated HLA-DRB1 in tumor cells were generally higher in anti-PD1 responders than in non-responders from Massachusetts General Hospital (MGH; *n* = 31; Fig. 7c, top) and the MD Anderson Cancer Center (MDACC; *n* = 11; Fig. 7c, bottom). Total tumor fucosylated HLA-DRB1 exhibited weak or no association with tumoral CD4^+^ T cells (Fig. 7d, top and bottom), although the association was modestly increased when restricted to CD4^+^ T cells localized at the periphery of the tumors (Extended Data Fig. 6b,c; absolute CD4^+^ T numbers in Supplementary Table 3). Importantly, we found that some specimens containing divergent tumor–stroma content did exhibit divergent correlation strengths (for example, core biopsies containing only tumor versus non-core biopsies containing substantial stroma). For example, a ‘highly correlated’ anti-PD1 responder (non-core biopsy) containing substantial tumor–stromal interface exhibited correlated high levels of fucosylated HLA-DRB1 and CD4^+^ T cells, whereas a ‘non-correlated’ responder (a core biopsy) did not (Extended Data Fig. 6d), suggesting that variable stromal content within biopsies may have at least partially undermined the strength of the correlations that we assessed.

The lack of significant correlation may also be attributed to the dynamic relationship between fucosylated HLA-DRB1 and CD4^+^ T cell infiltration that is further weakened by suboptimal inclusion criteria and/or patient stratification. Comparison of these markers in five patient-matched tumors before and after anti-PD1 therapy revealed no significant correlation in total HLA-DRB1 levels. However, before treatment, tumor cell fucosylation was significantly higher in the complete responder versus partial responders and non-responders; this dropped to the equivalently lower levels of the other patients after treatment. With the exception of one non-responder, the complete responder also exhibited significantly increased fucosylated HLA-DRB1 in tumor cells before treatment (Extended Data Fig. 6e). The consistent trends in tumor fucosylation and fucosylated tumor HLA-DRB1 observed across the three independent cancer center cohorts appear to support potential utility but importantly point to the need for further study in expanded patient pre-treatment biopsy cohorts that are controlled for a number of specific clinical variables, which will be discussed below.

## Discussion

Here, we report the administration of a dietary sugar as a way to increase itICs and enhance efficacy of the immune checkpoint blockade anti-PD1 agent. These studies reveal insights into the post-translational regulation and immunological roles of melanoma cell-expressed MHC-II proteins, further highlighting their relationship with itICs^42,43,45–49^. Specifically, fucosylation regulates the cell surface abundance of HLA-DRB1, which triggers robust CD4^+^ T cell-mediated itIC induction and melanoma suppression. It is important to acknowledge that our reliance on AAL lectin predominantly focuses our study on α1,6-fucosylated proteins. Although this does not diminish the crucial role that α1,6-fucosylated HLA-DRB1, which was identified as fucosylated via lectin-agnostic click chemistry mass spectrometric screening, plays in l-fuc-triggered anti-tumor immune responses, it is possible that proteins with other fucosylation linkages might contribute to aspects of anti-tumor immunity. It is also likely that the statistical strength of our analyses of tumor fucosylation with patient outcomes (Fig. 7) was limited by use of only AAL lectin, which precludes the detection of other structural forms of fucosylation. Nonetheless, the ability to leverage this mechanism using oral l-fuc administration may help to enhance other immunotherapeutic modalities (that is, other checkpoint inhibitors or adoptive cell-transfer therapies). Notably, as a non-toxic dietary sugar with a past safety precedent as an experimental therapy for children with leukocyte adhesion deficiency II^52,53^, l-fuc appears to be a potentially safe and tolerable therapeutic agent.

The consistent trends that we observed in higher tumor fucosylation and fucosylated HLA-DRB1 across anti-PD1 responders versus non-responders between the three independent cancer center cohorts support their potential utility as biomarkers of anti-PD1 responsiveness. However, further analyses in expanded patient biopsy cohorts are clearly needed. Considering the variable tumor-suppressive effects of l-fuc observed in our anti-PD1-treated SM1 and SM1 mouse models, there are likely similar biological and clinical variables in patients that must be further explored and that may have precluded statistical significance in our small analyses.

In terms of biological variables, how T cell biology is regulated by fucosylation, for example, has heretofore been unclear. Reported divergent effects of fucosylation on T cell activation versus exhaustion (that is, via regulation of PD-L1 expression) point to FUT-specific expression and roles that remain to be elucidated^54–56^. The fact that l-fuc does not alter the cell surface levels of PD-L1 in human or mouse melanoma cells (Extended Data Fig. 6a), suggesting that the discrepant tumor suppression by single-agent versus combination l-fuc and anti-PD1 therapy in our SW1 and SM1 mouse models (Fig. 5a), is attributed to determinants beyond the PD1–PD-L1 axis. Indeed, our global fucosylated and phosphoproteomic analyses suggest that fucosylation in CD4^+^ T cells impacts integrin β5, PKA and actin signaling (Extended Data Fig. 2) and that this is associated with increased intratumoral T cell presence and memory phenotypes in our models (Figs. 2 and 5), consistent with previous reports that those functions are regulated by those pathways in T cell biology^15–17,57^. The fact that l-fuc can increase CD4^+^ T central memory cells also partially explains how it can augment anti-PD1 efficacy, which is associated with the presence of these cells^10^. How l-fuc may regulate these signaling pathways and enrich for CD4^+^ T memory subsets within the tumor microenvironment and, furthermore, how l-fuc alters DC biology and induces their intratumoral accumulation (Figs. 2 and 5) may contribute to anti-tumor immune responses and tumor suppression in this context are unclear and warrant further lines of study. In addition, sex might be a determinant, as melanoma fucosylation levels are lower but correlate more strongly with intratumoral CD3^+^ T cells in male versus female patients (Fig. 1j,k). Reduced melanoma fucosylation, which is expected to lower itICs, might explain increased lethality in male patients (American Cancer Society Facts and Figures, 2022).

The availability of pre-treatment anti-PD1 tumor tissue specimens for this study was extremely limited. Thus, the specimens that we acquired were subject to clinical variability that may have undermined statistical robustness in our analyses. Subsequent studies investigating tumor fucosylation, total/fucosylated HLA-DRB1 and CD4^+^ T cells as biomarkers will need to factor for clinical variables including therapies received before anti-PD1 therapy and pre-existing medical conditions as well as time from biopsy to anti-PD1 treatment. Because we were unable to control for these confounders in our specimens, it is unclear how they may have impacted tumor and HLA-DRB1 fucosylation and CD4^+^ T cell biology and thus the strength of correlations between these markers and responsiveness to treatment. Likewise, the importance and contribution of the immune environment of the peri-tumoral stroma in this setting remains to be elucidated, as some of our biopsies contained stroma, whereas other tumor core biopsies did not. Prior studies focusing on tumor–immune interactions and immunotherapies (including anti-PD1 therapy) have highlighted the importance of analyzing biomarker staining patterns at tumor–immune and tumor–stromal interfaces contained within biopsies, as these are areas of enriched immunological activity and signaling^58–60^. Indeed, our observation of ‘highly correlated’ and ‘non-correlated’ anti-PD1 responder biopsies containing disparate amounts of tumor stroma highlight how lack of sufficient stroma in tumor biopsies likely undermined the statistical robustness of the correlations between fucosylated HLA-DRB1 and CD4^+^ T cells (Extended Data Fig. 6d). The acquisition of such biopsy specimens that are controlled for the variables detailed above is an important consideration for subsequent studies. Our findings highlight the need for a prospective clinical trial with defined protocols for collection of monotherapy anti-PD1 pre-treatment biopsies at defined time points proximal to therapy and clear biopsy protocols to yield tumor specimens that contain substantial intact stromal interface.

In conclusion, fucosylation of HLA-DRB1 is a key regulator of itIC abundance in melanomas, and this mechanism, together with fucosylation-regulated CD4^+^ T cell biology, can be therapeutically exploited using oral l-fuc administration. Elucidation of the mechanistic determinants is expected to advance our understanding of the immunobiology of melanoma and other cancers and to inform efforts in implementing fucosylation and/or fucosylated HLA-DRB1 as biomarkers and of l-fuc as a therapeutic agent.

## Methods

Our research complies with all relevant ethical regulations: all animal experiments were approved by the Moffitt IACUC committee. All patient specimen-staining analyses are considered as IRB exempt: investigators were blinded from all patient health information, and the specimens were previously collected under IRB-approved protocols per respective institutional IRB committees. All cell line and antibody information is provided in the Nature Portfolio Reporting Summary.

### General cell culture

The following cell lines were from the American Tissue Type Collection: A375, HEMN (normal adult epidermal melanocytes) and Jurkat. The following cell lines were from Rockland Immunochemicals: WM983A, WM983B, WM1366, WM115, WM266-4, WM164, WM793 and Lu1205. SW1 melanoma cells (gift from the Ronai laboratory at the Sanford Burnham Prebys Medical Discovery Institute) and SM1 melanoma cells (gift from the Smalley laboratory at the Moffitt Cancer Center) were cultured in DMEM containing 10% FBS, 1 g ml^−1^ glucose and 4 mM l-glutamine at 37 °C with 5% CO_2_. HEMN cells were grown in Lonza MGM-4 growth medium; before collection for IB analysis, the cells were switched to the same medium as the other cells overnight. Cell lines were transfected using Lipofectamine 2000 (Invitrogen). Primary CD4^+^ T cells were collected using the EasySep Human CD4^+^ negative selection isolation kit (Stemcell Technologies) according to the manufacturer’s protocols. Upon arrival at the laboratory, all cell lines are quarantined until they have passed footprint identification and mycoplasma testing (as mycoplasma negative). The identities of all cell lines (human and mouse) in the Lau laboratory are verified annually by short tandem repeat-based authentication ‘CellCheck’ services provided through IDEXX BioResearch.

### Cloning and mutagenesis

The gene encoding mFuk was cloned using cDNA from SW1 cells into the pLenti-C-Myc-DDK-IRES-Puro expression vector (OriGene Technologies) using BamHI and NheI restriction sites. Mouse EB1 constructs were cloned using cDNA from SW1 cells into the pLenti-C-Myc-DDK-IRES-Puro expression vector (OriGene Technologies) using AscI and XhoI restriction sites. pLKO shNT, pLKO shK1-1, pLKO shK1-2, pLKO shEB1-1 and pLKO shEB1-2 were obtained from MilliporeSigma. pLX304::EV was obtained from OriGene Technologies. pLX304::HLA-A and pLX304::HLA-DRB1 constructs were obtained from DNAasu^61^. HLA-DRB1^N48G^ and HLA-DRB1^T129A^ as well as EB1^N46G^ mutants were generated using the QuikChange II XL site-directed mutagenesis kit according to the manufacturer’s protocol (Agilent Technologies).

### Lectin pulldown

Control beads and AAL or UEA1 lectin-conjugated agarose beads were pre-blocked for 2 h in blocking buffer (2% IgG-free BSA (Jackson ImmunoResearch Laboratories)) on a rotator at 4 °C. Cells were lysed on ice in 1% Triton X-100 lysis buffer (1% Triton X-100, 20 mM Tris-HCl, pH 7.4, 150 mM NaCl in ddH_2_O with protease and phosphatase inhibitors (Thermo Fisher Scientific)), briefly sonicated and pelleted. The resulting lysates were normalized in protein concentration to the sample with the lowest concentration, diluted to a final concentration of 0.25% Triton X-100 with dilution buffer (0% Triton X-100, 20 mM Tris-HCl, pH 7.4, 150 mM NaCl in ddH_2_O with protease and phosphatase inhibitors), incubated with 15 µl pre-blocked beads (beads were centrifuged out of a block and resuspended in dilution buffer) and rotated overnight at 4 °C. Next, the beads were washed twice with dilution buffer and subjected to (12%) SDS–PAGE and IB analysis using the indicated antibodies.

### Mass spectrometric analyses

#### Profiling fucosylated proteins

EV-, pLenti-FUK-GFP- or shFUK-expressing WM793 cells were cultured in biological triplicate in the presence of 50 µM l-fuc-alkyne for ~72 h to ~80% confluence. Cells were lysed in 1.5% *N*-dodecyl-β-d-maltoside, 20 mM HEPES, pH 7.4, and protease and phosphatase inhibitors. Lysates were precipitated with acetone overnight, and pelleted proteins were resuspended and subjected to click chemistry labeling with biotin–azide using the Click-iT kit according to the manufacturer’s protocol (Invitrogen). The negative control included EV cells not labeled with l-fuc-alkyne. All biotinylated–fucosylated samples were pulled down using neutravidin beads (pre-blocked with 2% IgG-free BSA). Samples were submitted to the Sanford Burnham Prebys proteomics core facility for on-bead digestion and LC–MS/MS analysis. Hits that were increased by >1.5 fold in pLenti-FUK-GFP-expressing cells and unchanged or decreased in pLenti-EV-GFP-expressing cells or decreased in pLenti-shFUK-expressing cells were subjected to Ingenuity Pathway Analysis (Qiagen).

#### Profiling HLA-DRB1 glycosylation–fucosylation

Stained bands (~1 µg) of exogenously expressed V5–HLA-DRB1 purified from WM793 cells were minced, reduced and alkylated using 20 mM TCEP (Tris(2-carboxyethyl)phosphine) and iodoacetamide in 50 mM Tris-HCl. Gel pieces were washed with 20 mM ammonium phosphate in 50% methanol overnight at 4 °C, followed by dehydrating for 30 min with 100% acetonitrile. Samples were next digested with trypsin for 4 h at 37 °C and eluted through C_18_ ZipTips with 50% methanol and 0.1% formic acid (FA). Five microliters of the elution were diluted in 0.1% FA and injected into a Q Exactive Orbitrap mass spectrometer equipped with an Easy Nano-LC HPLC system and a reverse-phase column (Thermo Fisher Scientific). A binary gradient solvent system consisting of 0.1% FA in water (solvent A) and 90% acetonitrile with 0.1% FA in water (solvent B) was used to separate peptides. Raw data were analyzed using both Proteome Discoverer version 2.1 (Thermo Fisher Scientific) with the Byonic (Protein Metrics) module and Byonic standalone version 2.10.5. All extracted ion chromatograms were generated using Xcalibur Qual Browser version 4.0 (Thermo Fisher Scientific). The UniProt sequence Q5Y7D1_Human was used as the reference sequence for peptide analysis.

#### Phosphoproteomic profiling of CD4^±^ T cells

The indicated CD4^+^ T cells were lysed in RIPA buffer with protease and phosphatase inhibitors. Briefly, ~1 mg of each lysate was reduced with 4.5 mM dithiothreitol for 30 min at 60 °C, alkylated with 10 mM iodoacetamide at room temperature in the dark for 20 min and digested overnight at 37 °C with an enzyme-to-protein ratio of trypsin of 1:20 (Worthington). Resulting peptides were desalted using a reversed-phase Sep-Pak C_18_ cartridge (Waters) and lyophilized for 48 h. Lyophilized peptides were enriched for global phosphopeptides (pSTY) using IMAC Fe-NTA magnetic beads (Cell Signaling Technology) on a KingFisher Flex Purification System (Thermo Fisher Scientific), followed by SpeedVac concentration and resuspension in loading buffer (5% ACN and 0.1% TFA) before auto-sampling and LC–MS/MS as described below.

#### Fucoproteomic profiling of CD4^±^ T cells

The indicated CD4^+^ T cells were lysed in RIPA buffer with protease and phosphatase inhibitors and subjected to LPD using control or AAL beads. The beads were washed with PBS and subjected to on-bead trypsin digestion. Resulting peptides were further denatured with 30 mM ammonium bicarbonate at 95 °C for 5 min and then acidified with TFA at a final concentration of 1%. ZipTip-purified, eluted peptides were concentrated with a SpeedVac and resuspended in loading buffer (5% ACN and 0.1% TFA) before auto-sampling and LC–MS/MS as described below.

#### Profiling HLA-DRB1 interactors

V5-tagged WT or N48G glycofucomutant HLA-DRB1-expressing WM793 cells were lysed and subjected to V5 bead pulldown. Five percent of pulled down protein was immunoblotted to ensure equal sample submission for processing as described above and LC–MS/MS as described below.

### Liquid chromatography–mass spectrometry

Tryptic peptides were analyzed using a nanoflow ultra-high-performance liquid chromatograph and an electrospray Orbitrap mass spectrometer (RSLCnano and Q Exactive Plus, Thermo) for tandem MS peptide sequencing. Peptide mixtures were loaded onto a pre-column (100-µm ID × 2-cm column packed with C_18_ reversed-phase resin; particle size, 5 µm; pore size, 100 Å) and washed for 5 min with aqueous 2% acetonitrile and 0.1% FA. Solvent A comprised 98% ddH_2_O, 2% acetonitrile and 0.1% FA, and solvent B comprised 90% acetonitrile, 10% ddH_2_O and 0.1% FA. Trapped peptides were eluted or separated on a C_18_ analytical column (75-µm ID × 50 cm; particle size, 2 µm; pore size, 100 Å; Thermo Fisher Scientific) using a 90-min gradient at a flow rate of 300 nl min^−1^ of 2% to 3% solvent B over 5 min, 3% to 30% solvent B over 27 min, 30% to 38.5% solvent B over 5 min, 38.5% to 90% solvent B over 3 min and then held at 90% for 3 min, followed by 90% to 2% solvent B in 1 min and re-equilibrated for 18 min. MS resolution was set at 70,000, and MS/MS resolution was set at 17,500 with a maximum IT of 50 ms. The top 16 tandem mass spectra were collected using data-dependent acquisition following each survey scan and 60-s exclusion for previously sampled peptide peaks. For phosphoproteomic, fucoproteomic and HLA-DRB1 WT and glycofucomutant interactor profiling, MaxQuant^62^ software (version 1.6.2.10) was used to identify and/or quantify phosphopeptides and proteins for the data-dependent acquisition runs. The false discovery rate was set to 1%.

### PyMOL structural modeling

In Fig. 4a, structural modeling was performed using PyMOL (Molecular Graphics System, version 2.0, Schrödinger) of the HLA-DRB1–HLA-DM complex (PDB ID 4FQX), HLA-DRB1 (yellow) and DM (gray). For the CD4–HLA-DRB1–TCR complex, the model was reconstituted by superimposing the DRB1 β-chains from the CD4–HLA-DR1 complex (PDB ID 3S5L) and the TCR–HLA-DR1 complex (PDB ID 6CQR) using PyMOL. RMSD between the 163 backbone atoms is 0.497. The potential glycosylation sites N48 and T129 of the HLA-DR1 β-chain are shown as sticks. The color scheme is CD4 (cyan), HLA-DRB1 (yellow), antigen peptide (magenta) and TCR (green).

### Tumor-infiltrating lymphocyte-isolation protocol

Tumors of SW1 or SM1 melanoma cells from C3H/HeJ or C57BL/6 mice, respectively, were digested using 1× tumor digest buffer (0.5 mg ml^−1^ collagenase I, 0.5 mg ml^−1^ collagenase IV, 0.25 mg ml^−1^ hyaluronidase V, 0.1 mg ml^−1^ DNase I in HBSS (MilliporeSigma)) and homogenized using a Miltenyi MACS dissociator. Red blood cells were lysed using ACK lysis buffer (Life Technologies). Tumor homogenate cells were counted using a standard hemocytometer.

### Human donor peripheral CD4^±^ T cell-isolation protocol

Human CD4^+^ T cells were (1) isolated from fresh PBMCs using a CD4^+^ T cell negative selection isolation kit (Stemcell Technologies) according to manufacturer’s protocols, (2) cultured in the presence of vehicle or 250 µM l-fuc and (3) activated using anti-CD3/CD28 Dynabeads (Thermo Fisher Scientific) at a bead:CD4^+^ T cell ratio of 1:1. After 48 h, cell pellets were collected and lysed for fucoproteomic or phosphoproteomic profiling.

### Flow cytometry

Gating schemes are provided in the Supplementary Information. Unless otherwise indicated, cytometry was performed using an LSR Flow Cytometer (BD Biosciences), and analysis was performed using FACSDiva version 9, CellQuest version 6 and FlowJo version 9 software (BD Biosciences).

#### Intratumoral immune cell and splenic profiling

Total itICs were gated first to single cells as described in Supplementary Fig. 1. Single-cell suspensions from tumor and spleen tissue were stained with Live/Dead Zombie NIR (BioLegend) at 1:1,000 in PBS for 20 min. Cell suspensions were centrifuged and stained with the following with antibodies at 0.5 µg ml^−1^ per antibody: APC anti-mouse CD3, Pacific Blue anti-mouse CD4, BV785 anti-mouse CD8, PerCP anti-mouse CD25, FITC anti-mouse F4/80, Pe-Cy7 anti-mouse CD11c, PE anti-mouse NK1.1 or PE anti-mouse DX5 and PerCP-Cy5.5 anti-mouse CD11b. Compensation controls were prepared using 0.5 µg ml^−1^ of each antibody with UltraComp eBeads (Thermo Fisher Scientific). After staining, cells were washed, fixed (2% formaldehyde), washed and subjected to flow cytometric analysis. Dendritic and CD4^+^ T cell subpopulations were subjected to staining and flow cytometric analyses as in Supplementary Figs. 2 and 3.

#### Assessment of cell surface fucosylation, HLA-DRB1 and PD-L1

The indicated cells were treated for 72 h with DMSO, 250 µM FUTi (MilliporeSigma) or 250 µM l-fuc (Biosynth), followed by staining with 0.1 µM PKH26 (MilliporeSigma) before fixation in 4% formaldehyde solution. Cells were stained with anti-HLA-DRB1 antibody and fluorescein AAL or anti-human or anti-mouse PD-L1 antibodies overnight, followed by three washes and staining with Alexa Fluor 594 donkey anti-rabbit antibody. Cells were washed and subjected to flow cytometric analyses using a FACSCalibur (BD Biosciences) as in Supplementary Fig. 4.

#### Assessment of cell surface pan-MHC-I and pan-MHC-II

Surgically resected patient tumors were minced to fragments less than 1 mm and digested in 1× tumor digest buffer. Single-cell suspensions were strained through 40-µm nylon mesh and counted for viability by trypan blue exclusion. Strained tumor homogenates were stained using Live/Dead Zombie NIR, PE anti-pan-MHC-I (HLA-A–HLA-C), FITC anti-pan-MHC-II, PerPCy5.5 anti-CD45, APC anti-CD90 and BV421 anti-EpCAM antibodies and subjected to flow cytometric analysis as in Supplementary Fig. 5.

### Immunoblot analyses

Cells were lysed on ice in standard RIPA buffer (25 mM Tris-HCl, pH 7.6, 150 mM NaCl, 5 mM EDTA, 1% NP-40 or 1% Triton X-100, 1% sodium deoxycholate, 0.1% SDS in diH_2_O with protease and phosphatase inhibitors), sonicated and pelleted, and the resulting lysates were normalized by protein concentration using the DC assay (Bio-Rad Laboratories). Lysates were subjected to (12%) SDS–PAGE and IB using the indicated antibodies. IB imaging and analysis was performed using either an Odyssey FC scanner and Image Studio (LI-COR Biosciences) or film.

### Quantitative PCR with reverse transcription

RNA from the indicated cells was extracted using the GenElute Mammalian Total RNA extraction system (MilliporeSigma) and reversed transcribed using the High-Capacity cDNA Reverse Transcription Kit (Thermo Fisher Scientific). RT–qPCR was performed using FastStart Universal SYBR Green Master Mix (Rox) (Roche Diagnostics) using CFX Manager version 3.1 on a Bio-Rad CFX96 Real-Time system (Bio-Rad Laboratories). RT–qPCR cycles were as follows: 95 °C for 10 min, 35 cycles of 95 °C for 15 s, 55 °C for 60 s and 72 °C for 30 s. Gene expression was normalized to histone H3A expression. Primers for RT–qPCR were generated using NCBI Primer BLAST software (National Center for Biotechnology Information). Oligonucleotide sequences are provided in Supplementary Table 4.

### Fluorescent immunocytochemical and immunohistological staining and analysis

#### General immunofluorescent cell staining

Cells were grown on German glass coverslips (Electron Microscopy Services) and fixed in fixation buffer (4% formaldehyde, 2% sucrose in PBS) for 20 min at room temperature. Cells were washed with PBS, permeabilized in permeabilization buffer (0.4% Triton X-100 and 0.4% IgG-free BSA (Jackson ImmunoResearch Laboratories) in PBS for 20 min at room temperature, washed with PBS again and incubated with the indicated antibodies. Unless otherwise indicated, images were acquired using a Keyence BZ-X710 microscope and processed and analyzed using ImageJ version 1.53a (NIH).

#### General immunofluorescent tissue staining

FFPE tumor sections (or tumor microarray (TMA) slides) were melted at 70 °C for 30 min, de-paraffinized using xylene and rehydrated in serial alcohol washes. The slides were pressure cooked at 15 psi for 15 min in 1× DAKO antigen-retrieval buffer (Agilent Technologies). Slides were subjected to two 5-min standing washes in PBS before blocking in 1× Carbo-Free Blocking Solution (Vector Laboratories) for 2–3 h, followed by two more washes and staining with the indicated lectin and/or antibodies before washing and mounting with VECTASHIELD and DAPI (Vector Laboratories).

#### Mouse tumor fucosylation analysis

For Extended Data Fig. 1a,d,k, tumors were immunostained with FITC-conjugated AAL lectin (0.4 µg ml^−1^) and rabbit anti-MART1 and rabbit anti-S100 antibodies. Images were acquired using a Keyence BZ-X710 microscope, and images were processed and analyzed using ImageJ version 1.53a (NIH) as follows: melanoma marker-positive regions were assigned as regions of interest in which we measured the integrated density of the AAL signal. Integrated densities of control tumors were assigned as 1, and integrated AAL density values of experimental tumors were divided by control values to produce relative fold changes and plotted as column charts.

#### Melanoma TMA analysis

For Fig. 1i–k, melanoma TMA (ME1002b; US Biomax; 18 female and 22 male patients, ages 25–88 years, stages 1A–4, cutaneous and mucosal tumors) was immunostained with FITC-conjugated AAL lectin (0.4 µg ml^−1^), rabbit anti-MART1, rabbit anti-S100 and anti-CD3 antibodies, followed by Alexa Fluor 568 (Cy3) donkey anti-rabbit and Alexa Fluor 647 (Cy5) donkey anti-mouse secondary antibodies. Slides were mounted with VECTASHIELD and DAPI. An Aperio ScanScope FL scanner (Leica Biosystems) was used to scan the TMA at 20×. Definiens Tissue Studio version 4.7 (Definiens) was used to identify individual cores followed by single-cell segmentation. Mean fluorescence intensity (MFI) values for fucosylation, melanoma markers and CD3 channels were extracted from each segmented cell; minimum MFI thresholds were set to enumerate melanoma and CD3^+^ T cells. Average MFI values for each core were reported for fucosylation and melanoma marker channels.

We used nonparametric Wilcoxon rank-sum test to compare melanoma-specific fucosylation levels between CD3^+^ T cell high versus low groups. Density values of CD3^+^ T cells were all log_2_ transformed in the statistical analysis. Multivariable linear regression was used to assess association between fucosylation and T cells while adjusting for confounding factors including sex, age and stage. The Spearman correlation coefficient was used to measure the correlation between melanoma-specific fucosylation and T cells in different sex groups.

#### Lectin-mediated proximity ligation assay

Coverslip-grown cells or FFPE tumor sections subjected to L-PLA were processed upfront as described above. Both approaches used mouse anti-HLA-DRB1 antibody (0.2 µg ml^−1^) and biotinylated AAL lectin (0.2 µg ml^−1^), staining overnight at 4 °C. Coverslips and/or slides were washed twice with PBS and incubated with phalloidin Alexa Fluor 488 with goat anti-biotin antibody for 2 h at 4 °C. Subsequent steps of the protocol were adapted from the Duolink In Situ Green PLA kit’s manufacturer’s protocol (MilliporeSigma). PLA anti-goat MINUS and PLA anti-mouse PLUS probes were applied at 1:5 for 1 h at 37 °C. The coverslips and/or slides were washed twice with wash buffer A before ligation with 1:5 ligation buffer and 1:40 ligase in ddH_2_O for 30 min at 37 °C. Coverslips and/or slides were washed twice with wash buffer A before incubation in amplification mix (1:5 amplification buffer and 1:80 polymerase in ddH_2_O for 1.5 h at 37 °C). Coverslips and/or slides were washed twice with wash buffer B before mounting with VECTASHIELD and DAPI.

#### Immunofluorescent staining and analysis of anti-PD1-treated patients with melanoma

For Fig. 5c, the indicated FFPE sections were immunostained with anti-HLA-DRB1 antibody or by L-PLA as detailed above with the addition of anti-CD4 antibody. WTS imaging was performed using the Vectra 3 Automated Quantitative Pathology Imaging System (PerkinElmer). Tiles (20× region of interest) were sequentially scanned across the slide and spectrally unmixed using inForm (PerkinElmer). HALO (Indica Labs) was used to fuse tile images together. For each whole-tumor image, (1) every individual melanoma marker (MART1 and S100)-positive cell was segmented and quantitatively measured for total fucosylation and total and fucosylated HLA-DRB1, and (2) every CD4^+^ T cell within the melanoma marker-positive tissue region and melanoma marker-negative periphery was counted. For each patient, marker values were displayed in box plots to visualize staining distribution of individual tumor cells. The total numbers of melanoma cells per patient section measured and analyzed were as follows: patient 1, 557,146 cells; patient 2, 743,172 cells; patient 3, 95,628 cells; and patient 4, 13,423 cells.

### Anti-PD1-treated patient specimens

#### Moffitt Cancer Center patient specimens

For Fig. 5d,e and Extended Data Fig. 4d,e, de-identified specimens from MCC patients with advanced stage melanoma were collected and analyzed following patient consent under MCC Institutional Review Board (IRB)-approved protocols:

For Fig. 7b, ‘responder’ patients exhibited >20 months of progression-free survival, whereas ‘non-responder’ patients progressed after <6 months after receiving anti-PD1 therapy.

For Extended Data Fig. 5h, non-response status to PD1 checkpoint blockade (nivolumab or pembrolizumab) was defined by RECIST 1.1 as disease progression on therapy or within 3 months of the last dose.

#### University of Texas MD Anderson Cancer Center patient specimens

Biospecimens were retrieved, collected and analyzed after patient consent under University of Texas MDACC IRB-approved protocols. University of Texas MDACC patients with advanced (stages III–IV) melanoma between 1 July 2015 and 1 May 2020 who received >1 dose of PD1 checkpoint blockade agent (nivolumab or pembrolizumab) were identified from detailed review of clinic records. Responders or non-responders were defined as those with a complete or partial response or stable or progressive disease, respectively, by RECIST 1.1. Pathologic response was defined by the presence or absence of viable tumors on pathologic review when available.

#### Massachusetts General Hospital patient specimens

Patients initiating anti-PD1 therapy (pembrolizumab) as front-line treatment for metastatic melanoma at MGH provided written informed consent for the collection of tissue and blood samples for research (DF/HCC IRB-approved protocol 11-181). Responders showed radiographic decrease in disease at initial staging for ≥12 weeks. Non-responders did not exhibit radiographic response and/or had rapid progression. Progression-free survival was defined as days from treatment start to radiographic scan when progression was first noted (uncensored) or last progression-free scan (censored). Overall survival was defined as days from treatment start to date of death (uncensored) or last follow-up (censored).

### Animal models

All animals were housed at the Vincent A. Stabile Research building animal facility at the MCC, which is fully accredited by the Association for Assessment and Accreditation of Laboratory Animal Care International (434) and is managed in accordance with the Guide for the Care and Use of Laboratory Animals, the Animal Welfare Regulations Title 9 Code of Federal Regulations Subchapter A, ‘Animal Welfare’, parts 1–3 (AWR), the Public Health Service Policy on Humane Care and Use of Laboratory Animals and by the USF Institutional Animal Care and Use Committee’s Principles and Procedures of Animal Care and Use. Experiments in this study received institutional approval by the Moffitt IACUC committee (protocol RIS00001625). Four-to-six-week-old female C3H/HeN and male C57BL6 mice were purchased from Charles Rivers Laboratories; immunodeficient NSG mice^63^ were from the Lau laboratory breeding colony. All mice were housed in rooms on a standard 12-h–12-h light cycle, with a temperature range of 68–72 °F and humidity range of 30–70%.

Power calculations determined cohort sizes to detect significant changes in tumor sizes. Generally, by using ten mice per group, we estimate that we will be able to detect a 10% difference in tumor development between any two conditions with *a P* value of 0.05 and a power value of 0.80 and a 20% change with *a P* value of 0.05 and a power of 0.95. This calculation has been used previously to designate groups of ten mice^9^, which accommodates for incidental loss of mice due to issues beyond our control (for example, tumor ulceration that requires exclusion from the study). Mouse tumor volumes were measured using length, width and depth divided by 2. At each experimental endpoint or if mice showed signs of metastatic disease, mice were humanely euthanized using CO_2_ inhalation in accordance with American Veterinary Medical Association guidelines.

For all mouse models, 1 × 10^6^ melanoma cells were injected subcutaneously in the right hind flanks of each mouse. Between 7 and 14 d, when tumor volumes reached ~150 mm^3^, mice were supplemented with or without 100 mM l-fuc (Biosynth) via drinking water, which was provided ad libitum^9^. This dosage is within previously reported ranges for dietary supplementation with l-fuc and other similar dietary sugars (for example, d-mannose) in rodent studies^64–68^. When tumors reached the endpoint volume (~2 cm^3^), animals were killed, and tumors and organs were processed for flow cytometric profiling or histological analysis as indicated. The maximum permitted tumor volume was not exceeded.

#### Models of control versus mFuk with or without l-fucose

For Fig. 1 and Extended Data Fig. 1, SW1 or SM1 mouse melanoma cells were injected into syngeneic C3H/HeN (or NSG) female or C57BL/6 male mice, respectively, as follows: parental SW1 cells for Fig. 1a, parental SM1 cells for Extended Data Fig. 1e, SW1 cells stably expressing either EV or mFuk for Fig. 1e and parental SW1 cells for Extended Data Fig. 1k.

#### Models of control versus l-fucose with or without FTY720

For Fig. 2b, SW1 mouse melanoma cells were injected into syngeneic C3H/HeN female mice. FTY720 (Cayman Chemicals) was administered at 20 µg every 2 d to inhibit lymph node egress^69^ starting on day 12, just before the initiation of l-fuc administration, until endpoint.

#### Immunodepletion mouse models

For Fig. 1l–o and Extended Data Fig. 1s–w, 3 d before tumor engraftment, PBS (control) or ~300 µg anti-CD4 (20 mg per kg) or anti-CD8 (20 mg per kg) antibodies were administered by intraperitoneal injection into the indicated cohorts of mice. Immunodepletion antibody or PBS injections were continued every 3–4 d until endpoint. Syngeneic recipient C3H/HeN female or C57BL/6 male mice were injected with SW1 or SM1 cells, respectively.

#### HLA-A and HLA-DRB1 knockdown and glycofucomutant H2EB1 reconstitution mouse model

For Figs. 3d–h and 4f, SW1 mouse melanoma cells expressing shNT, shH2K1, shEB1, shNT and EV, shEB1 and EV, shEB1 and EB1^WT^, or shEB1 and EB1^N46G^ were injected into syngeneic C3H/HeN female mice.

#### Anti-PD1 mouse model

For Fig. 5, SW1 or SM1 mouse melanoma cells were injected into syngeneic C3H/HeN female or C57BL/6 male mice, respectively. After approximately 7 d, when the mouse tumors reached ~150 mm^3^, the mice were supplemented either with or without 100 mM l-fuc via drinking water, which was provided ad libitum. Simultaneously, PBS (control) or anti-PD1 antibody (20 mg per kg) was administered via intraperitoneal injection every 3–4 d until endpoint.

### Statistics and reproducibility

GraphPad Prism version 8 was used for statistical calculations unless otherwise indicated. For all comparisons between two independent conditions, *t*-tests were performed to obtain *P* values and s.e.m. For comparisons between ≥2 groups, one-way or two-way ANOVAs were performed, and *P* values and s.e.m. were obtained. For the TMA data, the Wilcoxon signed-rank test was used to determine significance. Data distribution was assumed to be normal but this was not formally tested; we have included individual data points in all relevant plots.

For molecular and cell-based assays, experiments were performed in three biologically independent replicates with similar results and outcomes. No data were excluded from analyses. Only when cell lines were either contaminated or lost knockdown or exogenous expression were the cell lines regenerated and the experiments performed again (that is, more than three times total). For cost feasibility, three biologically independent specimens were pooled for mass spectrometric profiling experiments, which were performed once. However, post-profiling validation experiments were performed in three biologically independent experiments with similar results.

Each of the 12 unique mouse models was performed once. However, with the exception of the NSG mouse model (Extended Data Fig. 1k), each mouse model contained built-in repeat control groups (for example, control and l-fuc-fed-only groups) that were repeated in at least one of the other models. Furthermore, SW1 mouse models were replicated in SM1 mouse models, which show conservation of our results across different melanoma mutational background and strains and sex of mice. No mice were excluded from the analyses unless tumors ulcerated or did not graft successfully before treatment.

In general, for molecular, cell-based and mouse-based experiments, the investigators were not blinded to allocation during experiments and outcome assessment; randomization was not used in these cases. However, the investigators were blinded to allocation during TMA and patient specimen immunostaining, signal measurement and initial analysis.

### Reporting summary

Further information on research design is available in the Nature Portfolio Reporting Summary linked to this article.

## Supplementary information

Supplementary Information Supplementary Figs. 1–5

Reporting Summary

Supplementary Tables Supplementary Table 1. Flow cytometric source data for the SW1 mouse model of l-fuc with or without FTY720 (Fig. 2). Supplementary Table 2. Flow cytometric source data for the SM1 mouse model of l-fuc with or without anti-PD1 therapy (Fig. 5). Supplementary Table 3. Single-cell segmented immunofluorescent staining-derived data values for Moffitt Cancer Center, MGH and MDACC patient tumor sections. Supplementary Table 4. Oligonucleotide sequences.

## Data Availability

Mass spectrometry data have been deposited in ProteomeXchange with the primary dataset identifiers as follows: Extended Data Fig. 2a, PXD038065; Extended Data Fig. 2e, PXD038636; Extended Data Fig. 3a, PXD038303; Extended Data Fig. 5a, PXD038068. All other data supporting the findings of this study are available from the corresponding author on reasonable request. Source data are provided with this paper.

## Source data

Source Data Figs. 1–7 and Extended Data Figs. 1–6 Statistical source data for all figures (figures and items indicated on individual tabs).
Source Data Fig. 3 Unprocessed blots for Fig. 3.
Source Data Fig. 4 Unprocessed blots for Fig. 4.
Source Data Extended Data Fig. 1 Unprocessed blots for Extended Data Fig. 1.
Source Data Extended Data Fig. 2 Unprocessed blots for Extended Data Fig. 2.
Source Data Extended Data Fig. 4 Unprocessed blots for Extended Data Fig. 4.
Source Data Extended Data Fig. 5 Unprocessed blots for Extended Data Fig. 5.

## Code availability

No custom code was used in this study.
