# Supplementary material for: Fucosylation of HLA-DRB1 regulates CD4+ T cell-mediated anti-melanoma immunity and enhances immunotherapy efficacy
Source: Nat Cancer. 2023 Jan 23;4(2):222–39. doi: 10.1038/s43018-022-00506-7 (PMC9970875; doi:10.1038/s43018-022-00506-7)
Supplement: Supplementary file 1 — Supplementary Figs. 1–5 [file 43018_2022_506_MOESM1_ESM.pdf]

nature cancer

Article https://doi.org/10.1038/s43018-022-00506-7

Fucosylation of HLA-DRB1 regulates CD4^{+} T cell-mediated anti-melanoma immunity and enhances immunotherapy efficacy

In the format provided by the authors and unedited

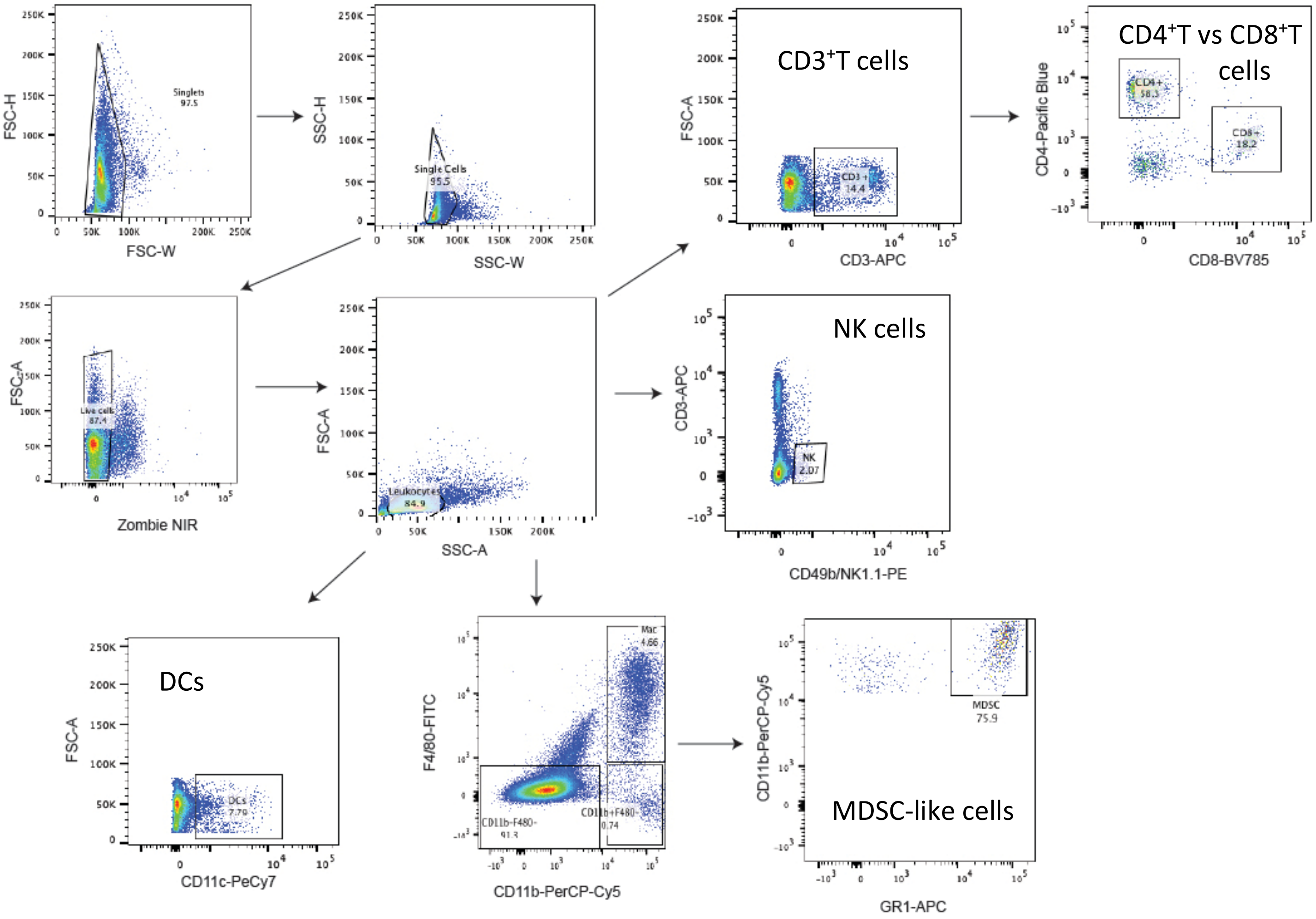

Figure S1: Intratumoral immune cell (itIC) gating panel. Intratumoral immune cell (itIC), intra lymph node immune cell, and splenocyte flow cytometry gating scheme (relevant to Fig. 1b-d, f-h, 2c-e, 3g,h, 5b, Extended Data Fig. 1b,c,f,g,h,i,j,m,n,p,q,v,w, 5f, 5g,). itICs or splenocytes were harvested as indicated in the Methods. 1 x 10^6 itICs or splenocytes were fixed and immunofluorescently stained with the indicated itIC subpopulation markers. As shown above, the cells were first gated for FSC/SSC and live/dead cell stain (Zombie NIR; were stained with Live/Dead Zombie NIR (Biolegend, (San Diego, CA)) at 1:1,000 in PBS for 20 min). Live cells were gated from the Zombie-negative population. ItICs were gated based on splenocyte size from a control spleen. Individual immune subpopulations were sub-gated from the total TIL population using the following staining criteria: $CD3^+$ for $CD3^+$ T cells; $CD3^+/CD4^+/CD8^-$ for CD4+T cells; $CD3^+/CD4^-/CD8^+$ for $CD8^+$ T cells, $CD11c^+/CD11b^+$ for DCs; either NK1.1 (for C57/BL6 mice) or DX5 (for C3H/HeJ) for NK cells; $CD11b^+/GR1^+$ for MDSC-like cells; and $F4/80^+$ for macrophages.

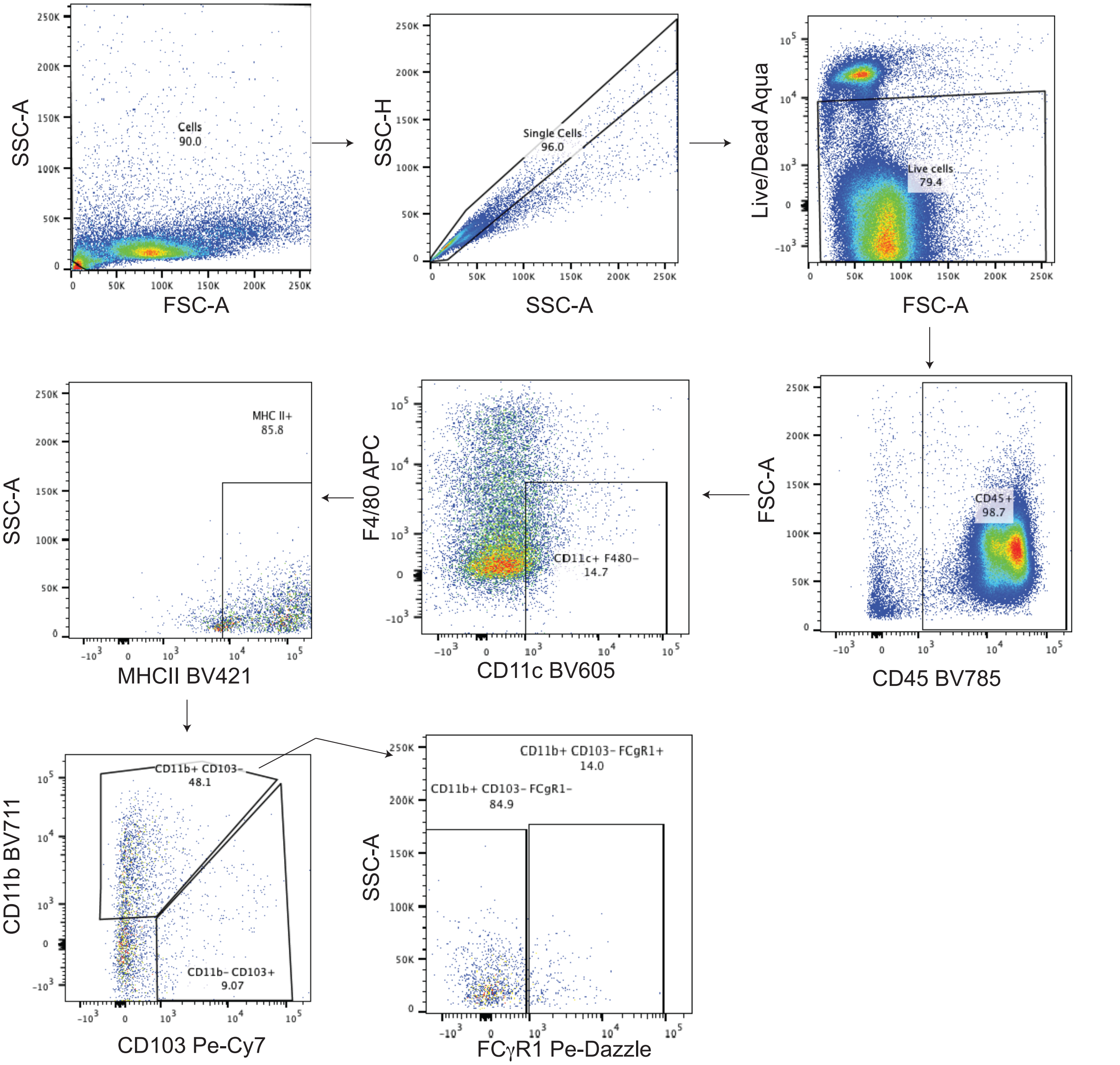

Figure S2: Dendritic cell gating panel for FTY720 and anti-PD1 mouse models (Relevant to Fig. 2c and 5b). SW1 or SM1 melanoma tumors or lymph nodes were harvested and processed as indicated in the Methods section. 2 x 10^6 cells for tumors and 2.5 x 10^5 for lymph nodes were fixed and immunofluorescently stained with indicated antibody. As detailed above, the cells were first gated for FSC/SSC, followed by live/dead stain (live/dead aqua). After gating for $CD45^+$ cells, dendritic cells were gated for CD11c, using F4/80 to gate out macrophages/monocyte populations. For specific dendritic cell populations, $MHCII^+$ cells were gated by CD103 and CD11b to identify cDC1s and FCγR1 was used to identify cDC2s and monocyte-derived dendritic cells (moDCs).

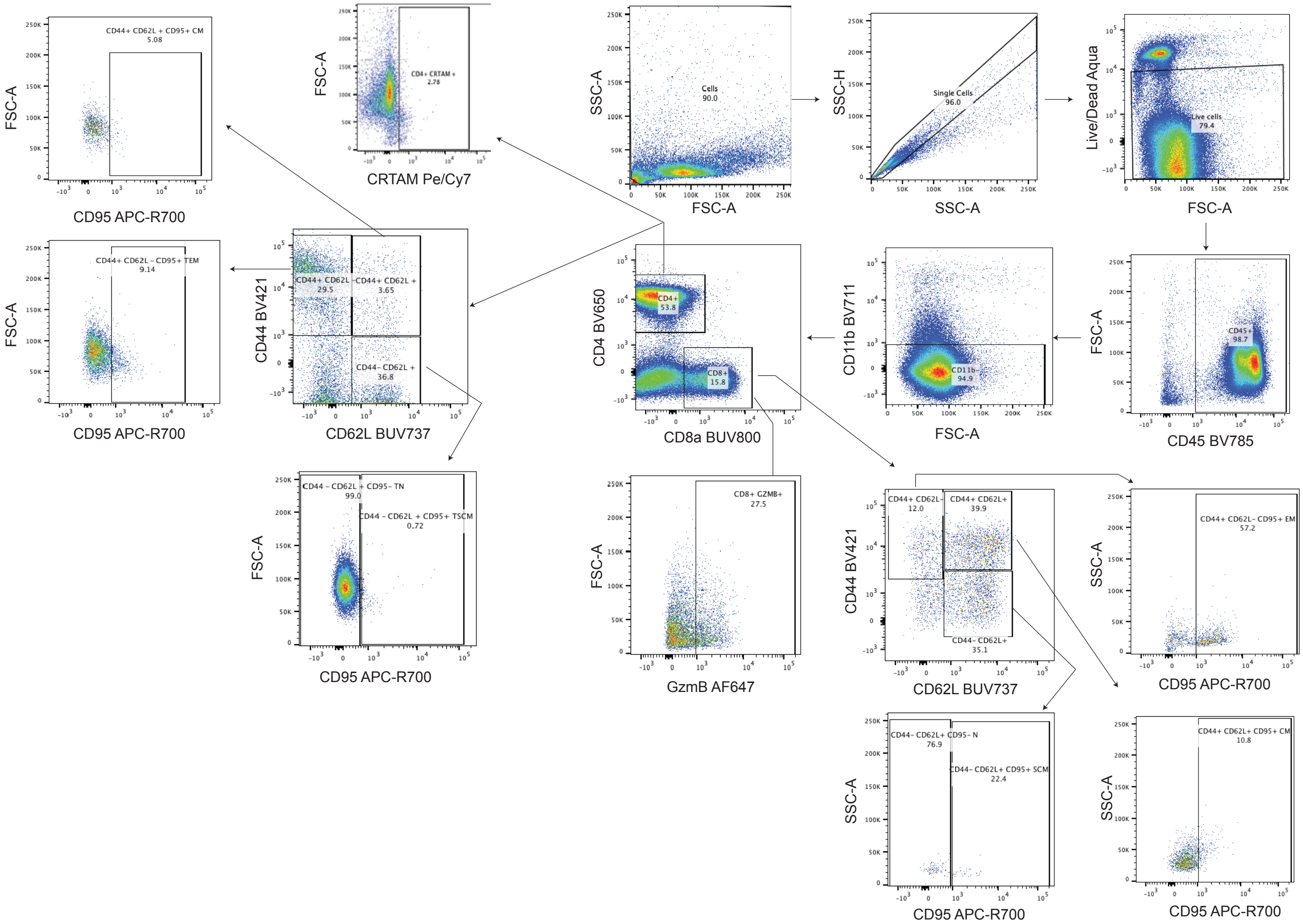

Figure S3: Memory and cytotoxic phenotype of CD4$^+$ and CD8$^+$ T cell gating panel for FTY720 and anti-PD1 mouse models (Fig. 2c, 2d, 2e, and 5b). SW1 or SM1 melanoma tumors or lymph nodes were harvested and processed as indicated in the Methods section. 2 x 10^6 cells for tumors and 2.5 x 10^5 for lymph nodes were fixed and immunofluorescently stained with indicated antibody. As detailed above, the cells were first gated for FSC/SSC, followed by live/dead stain (live/dead aqua). After gating for CD45$^+$ cells, CD11b was used to exclude myeloid cells from the analysis. CD4$^+$ and CD8$^+$ subpopulations were gated from this, followed by CRTAM and Granzyme B as cytotoxic markers. For memory phenotypes, CD4$^+$ and CD8$^+$ cells were gated by CD62L and CD44, followed by CD95.

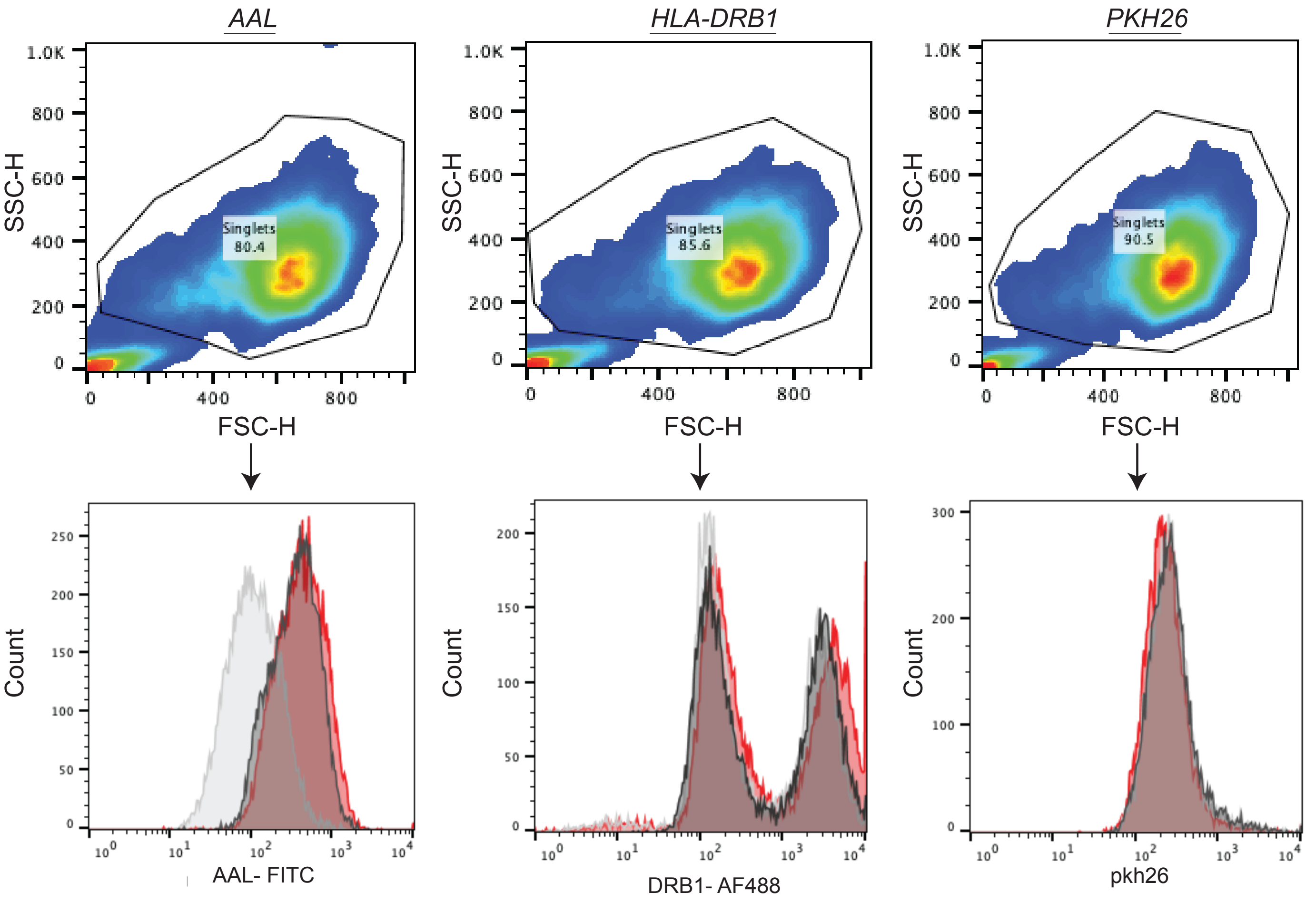

	Sample	MFI
	WM793 DMSO	351
	WM793 FUTi	104
	WM793 L-fuc	404

	Sample	MFI
	WM793 DMSO	335
	WM793 FUTi	230
	WM793 L-fuc	357

	Sample	MFI
	WM793 DMSO	248
	WM793 FUTi	249
	WM793 L-fuc	218

Figure S4: Cell surface fucosylation gating panel. Cell surface fucosylation/HLA-DRB1/PD-L1 flow cytometry gating scheme (Relevant to Fig. 4e and Extended Data Fig. 4b, 6d). Melanoma cells were harvested and processed as indicated in the Methods section. As detailed above, the cells were first gated for FSC and SSC followed by gating for indicated stains. A minimum of $1x10^4$ cells were analyzed within the singlet SSC-FSC gate for analysis of mean fluorescence intensity (MFI) of AAL-FITC, HLA-DRB1-AF488, or PKH26. AAL and HLA-DRB1 values were normalized to the respective PKH26 values (which reflect plasma membrane surface area) in order to discern AAL or HLA-DRB1 intensity per plasma membrane area.

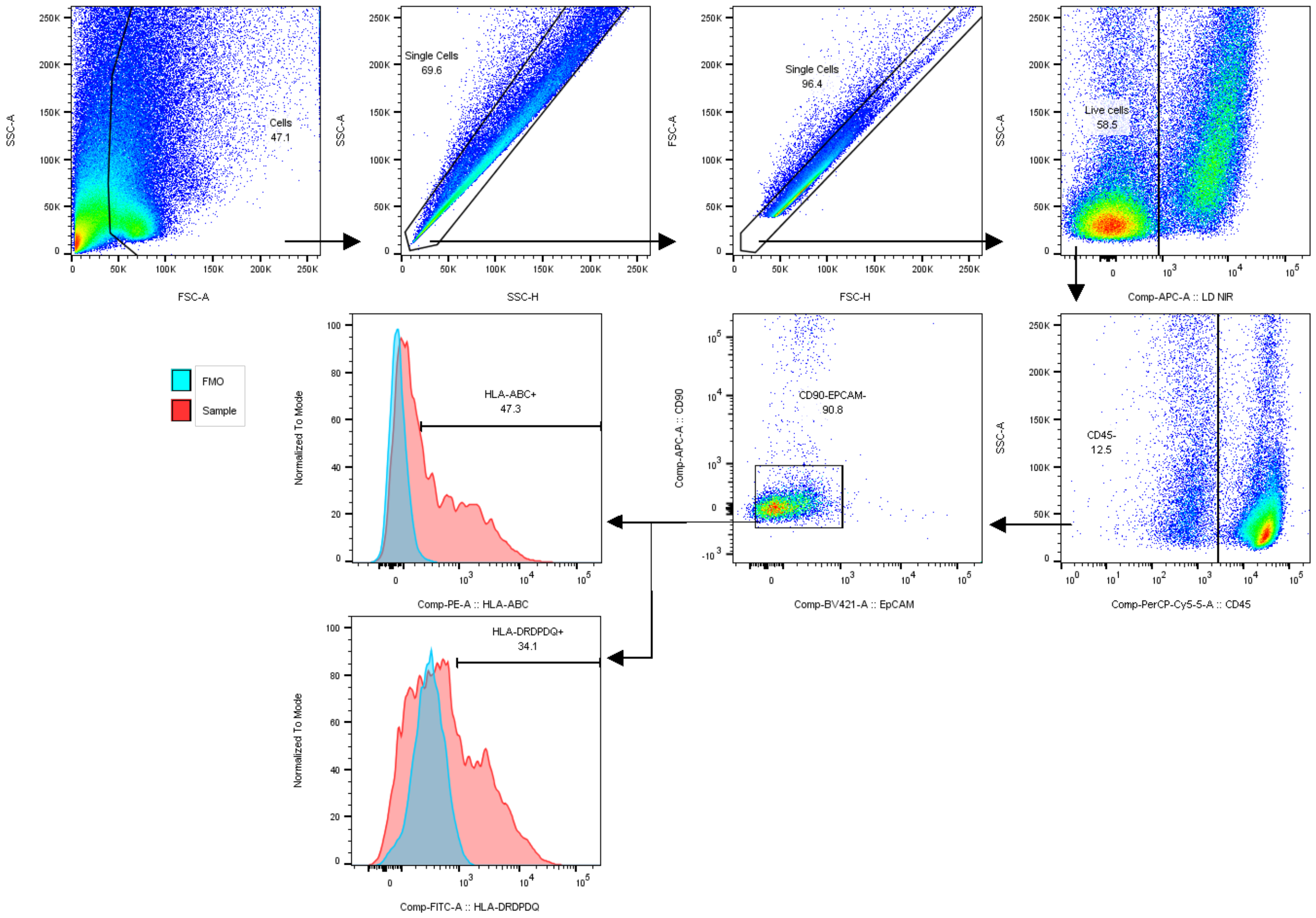

Figure S5: Patient specimen tumor cell surface MHC-I and MHC-II gating panel (Relevant to Extended Data Fig. 5h). Patient-derived melanoma tumor homogenates were processed and stained as described in the Methods section. As detailed above, $5x10^5$ stained single cells were first gated for FSC and SSC followed by gating for live cells followed by gating for CD45 and CD90/EPCAM negative cells. Cells were then profiled for pan MHC-I (HLA-A/B/C) or pan MHC-II (HLA-DR/DP/DQ). MHC-I and MHC-II expression was dichotomized as positive or negative based on FMO samples for each marker.
