# Supplementary material for: Fucosylation of HLA-DRB1 regulates CD4+ T cell-mediated anti-melanoma immunity and enhances immunotherapy efficacy
Source: Nat Cancer. 2023 Jan 23;4(2):222–39. doi: 10.1038/s43018-022-00506-7 (PMC9970875; doi:10.1038/s43018-022-00506-7)
Supplement: Supplementary file 2 — Reporting Summary [file 43018_2022_506_MOESM2_ESM.pdf]

nature portfolio

Corresponding author(s): Eric K. Lau

Last updated by author(s): Dec 14, 2022

Reporting Summary

Nature Portfolio wishes to improve the reproducibility of the work that we publish. This form provides structure for consistency and transparency in reporting. For further information on Nature Portfolio policies, see our Editorial Policies and the Editorial Policy Checklist.

Statistics

For all statistical analyses, confirm that the following items are present in the figure legend, table legend, main text, or Methods section.

n/a	Confirmed	
☐	☒	The exact sample size (*n*) for each experimental group/condition, given as a discrete number and unit of measurement
☐	☒	A statement on whether measurements were taken from distinct samples or whether the same sample was measured repeatedly
☐	☒	The statistical test(s) used AND whether they are one- or two-sided<br>*Only common tests should be described solely by name; describe more complex techniques in the Methods section.*
☐	☒	A description of all covariates tested
☐	☒	A description of any assumptions or corrections, such as tests of normality and adjustment for multiple comparisons
☐	☒	A full description of the statistical parameters including central tendency (e.g. means) or other basic estimates (e.g. regression coefficient) AND variation (e.g. standard deviation) or associated estimates of uncertainty (e.g. confidence intervals)
☐	☒	For null hypothesis testing, the test statistic (e.g. *F*, *t*, *r*) with confidence intervals, effect sizes, degrees of freedom and *P* value noted<br>*Give P values as exact values whenever suitable.*
☒	☐	For Bayesian analysis, information on the choice of priors and Markov chain Monte Carlo settings
☒	☐	For hierarchical and complex designs, identification of the appropriate level for tests and full reporting of outcomes
☐	☒	Estimates of effect sizes (e.g. Cohen's *d*, Pearson's *r*), indicating how they were calculated

Our web collection on statistics for biologists contains articles on many of the points above.

Software and code

Policy information about availability of computer code

Data collection	For flow cytometry, we used Cellquest v6 or FACSDiva v9 software (BD Biosciences) on a LSR Flow Cytometer (BD Biosciences) for collection of data.<br>For qRT-PCR, we used CFX Manager v3.1 software on a CFX96 Touch Real-Time PCR Detection System (Bio-Rad)
Data analysis	Usage of these software in specific experiments are explained further in the main text and Methods sections.<br><br>For mass spectrometry, data were analyzed using both Proteome Discoverer v2.1 (ThermoFisher Scientific) with Byonic (Protein Metrics) as a module and Byonic standalone v2.10.5. All extracted ion chromatograms (EICs) were generated using Xcalibur Qual Browser v4.0 (ThermoFisher Scientific). MaxQuant software (version 1.6.2.10) was used to identify and quantify the proteins for the DDA runs.<br>For pathway analyses of our fucosylated proteomic hits, we used Ingenuity Pathway Analysis (Qiagen).<br>For N-linked glycosylation site prediction of HLA-DRB1, we used NetNGlyc 1 (DTU Bioinformatics).<br>For O-linked glycosylation site prediction HLA-DRB1, we used NetOGlyc 4 (DTU Bioinformatics).<br>For structural modeling, we used PyMOL v2.0 (Molecular Graphics System, Schrödinger, LLC).<br>For flow cytometry, we used FlowJo v9 software (BD Biosciences) for analysis.<br>For analysis of mouse tumor images (e.g., fucosylation intensity), we used ImageJ v1.53a (NIH).<br>For general statistical analyses, we used Prism v8 (GraphPad).

For manuscripts utilizing custom algorithms or software that are central to the research but not yet described in published literature, software must be made available to editors and reviewers. We strongly encourage code deposition in a community repository (e.g. GitHub). See the Nature Portfolio guidelines for submitting code & software for further information.

Data

Policy information about availability of data

All manuscripts must include a data availability statement. This statement should provide the following information, where applicable:

- Accession codes, unique identifiers, or web links for publicly available datasets
- A description of any restrictions on data availability
- For clinical datasets or third party data, please ensure that the statement adheres to our policy

Mass spectrometry data have been deposited in ProteomeXchange with the primary dataset identifiers as follows: Extended Data Fig. 2a: PXD038065; Extended Data Fig. 2e: PXD038636; Extended Data Fig. 3a: PXD038303; Extended Data Fig. 5a: PXD038068. Source data for Figs. 1, 2, 3, 4, 5, and 7 and Extended Data Figs. 1,2, 3, 4, 5, and 6 have been provided as Source Data files. The source data for the single-cell-level dot plots in Figs. 7bi, 7bii, and 7biii are provided via figshare (https://doi.org/10.6084/m9.figshare.21433155). All other data supporting the findings of this study are available from the corresponding author on reasonable request.

Human research participants

Policy information about studies involving human research participants and Sex and Gender in Research.

Reporting on sex and gender

This study focuses on basic biological mechanisms regulating tumor immunological interactions in melanoma, and as such, sex and gender were not predominant topics of study. However, factoring for biological variability, patient-derived de-identified specimens included tissues from both female and male patients. Inclusion criteria was not sex-based but rather based on availability, and specifically, confirmed diagnosis of melanoma and treatment with anti-PD1 immune checkpoint blockade therapy. In general, there were more male than female patient specimens, which likely reflects the higher incidence rate of melanoma in men than women.

Population characteristics

The tumor microarray used in Fig. 1i-1j is a commercial product from US Biomax. This microarray includes specimens from 18 female and 22 male patients; age range: 25-88; tumor stage range: 1A-4.

For Figs. 6 and 7 and Extended Data Fig. 6, patient specimen characteristics were as follows:
Moffitt Cancer Center patients: 2 female and 2 male patients, age range: 51-80
University of Texas MD Anderson Cancer Center patients: 3 female and 8 male patients, age range: 44-76
Massachusetts General Hospital patients: 10 female and 21 male, age range: 41-82

The PBMCs used in this study were purchased from LifeSouth without sex-based inclusion criteria and were derived from deidentified healthy male and female donors between the ages of 30-50 (average age range).

Recruitment

The tumor microarray used in Fig. 1i-1j is a commercial product from US Biomax, and thus the construction of the microarray was subject to availability and out of our control. We chose this particular microarray as it represented relatively even representation of female vs. male patients, staging, and tumor sites.

For Figs. 6 and 7 and Extended Data Fig. 6, consented patients with advanced stage melanoma who received anti-PD1 immune checkpoint blockade therapy between 2014 and 2020 at Moffitt Cancer Center, UT MD Anderson Cancer Center, or Massachusetts General Hospital were identified, and specimens collected and analyzed following patient consent under respective Institutional Review Board-approved protocols.
Responder status criteria:
•For Moffitt Cancer Center: patients exhibiting >20 months of progression-free survival were considered as “Responders”, whereas “Non-Responder” patients progressed in less than 6 months after receiving anti-PD1.
•For UT MD Anderson Cancer Center: responder status was defined as a complete or partial response and non-responder was defined as stable or progressive disease by RECIST 1.1. Pathologic response was defined by the presence or absence of viable tumor on pathologic review when available.
•For Massachusetts General Hospital: patients were classified as responders (R) who showed clear radiographic decrease in disease at initial staging through a minimum of 12 weeks. Patients classified as non-responders (NR) did not respond to treatment radiographically and/or had clear and rapid progression. Progression free survival (PFS) is given in days from treatment start to radiographic scan when progression was first noted (uncensored) or last progression free scan (censored). Overall survival (OS) is given in days from treatment start to date of death (uncensored) or last follow-up (censored).

The fresh PBMCs were purchased from LifeSouth bloodbank. The lots of PBMCs were acquired from deidentified healthy male and female donors. There were no recruitment criteria except that they are healthy and between the ages 30-50 (average age range).

Ethics oversight

For the TMA, donors provided consent prior to specimen donation to US Biomax. For Figs. 6 and 7 and Extended Data Fig. 6, previous studies from which this study acquired Moffitt patient specimens were performed under Moffitt's Institutional Review Board-approved protocols including the Total Cancer Care protocol. All patient samples were de-identified prior to use in research studies. All patients signed approved consent forms. All patient specimens acquired from the University of Texas MD Anderson Cancer Center and Massachusetts General Hospital were collected prior to this study for other studies and protocols that were previously approved by their respective Institutional Review Boards. Fresh PBMCs were purchased as a de-identified products from healthy human donors from LifeSouth bloodbank. Donors were consented by LifeSouth prior to donation.

Note that full information on the approval of the study protocol must also be provided in the manuscript.

Field-specific reporting

Please select the one below that is the best fit for your research. If you are not sure, read the appropriate sections before making your selection.

☒ Life sciences ☐ Behavioural & social sciences ☐ Ecological, evolutionary & environmental sciences

For a reference copy of the document with all sections, see nature.com/documents/nr-reporting-summary-flat.pdf

Life sciences study design

All studies must disclose on these points even when the disclosure is negative.

Sample size

For mouse models: We planned all experiments to implement 10 mice per group. With this number, we estimated the ability to detect a 10% difference in tumor development between any 2 conditions with a p-value of 0.05 and a power of 0.80, and a 20% change with a p-value of 0.05 and a power of 0.95. In our experience, 10 mice per group have been more than sufficient to provide statistical power and buffer for incidental loss of mice due to factors outside of our control (e.g., aberrant tumor ulceration resulting in exclusion of the animal from the results). This calculation has been used previously to designate groups of 10 mice (Lau et al. Sci Signal 2015).

For patient specimen studies: our cohort numbers were limited by availability and inclusion criteria from each cancer center.

For all other experiments involving other living entities (i.e., cell-based assays), every experiment was performed in at least standard biological triplicate. Experiments entailing specific sample sizes included flow cytometry. For all flow cytometric analyses, we stained and analyzed 1x10^6 per mouse tumor for at least 3 mice per treatment group per standard protocols from previous publications of our collaborator and co-author, Dr. Shari-Pilon Thomas. For cell surface flow cytometric analysis experiments using human or mouse melanoma cell lines, we stained ~1x10^5 cells per sample but used a gating and analysis standard of 10,000 cells per sample.

As the study focus was not a sex-associated biological mechanism, we implemented both male and female mouse models as well as a tumor microarray with ~ equal distribution of male and female melanoma tissue donors, and both male and female bulk tumor tissue donors.

Data exclusions

No data were excluded from the analyses.

Replication

All cell-based experiments were repeated in independent biological triplicates. In addition, to ensure reproducibility, key experiments (for example, lectin pulldown and IP-lectin blot for HLA-DRB1 to demonstrate fucosylation) were performed independently by Lau laboratory members other than those who performed the experiments for the data shown in the Figures). All of the data shown include "successful" replication attempts; "unsuccessful" replication attempts that are not shown include for example, if on rare occasion, cells either were contaminated or lost stable expression of a viral construct. In those cases, cells were either re-cultured or parental cells were re-transduced to generate indicated cell lines, and the experiments were repeated until at least 3 independent biological replicates were attained. In no case, ever, were experiments repeated due to bias.

Importantly, for the 12 mouse models showcased in this study, each successive mouse experiment generally included independently repeated control and experimental conditions from a preceding experiment, ensuring evidence of reproducibility and consistency. For example, control vs. fucose fed treatment mouse groups (no other injections or viral modifications, etc.) were independently replicated 7 times.

For mass spectrometric experiments, mass spectrometry was performed once on samples pooled from 3 biological replicates. Subsequent validation was performed in biological triplicate.

Randomization

For cell- and mouse-based experiments, treatment groups were delineated as indicated in each experiment/Figure (detailed treatment group designs specific to each of the 12 mouse models in this study are in the Methods section); randomization was not applicable for these experiments.
For the patient specimen staining analyses:
Tumor microarray staining (Fig. 1i-k) was by definition randomized, as all biopsies were simultaneously stained and scanned.
Patient tumor biopsy staining (Fig. 5c-e): We obtained blinded patient specimens from Moffitt, MD Anderson, and Harvard/Massachusetts General Hospital. We immunostained and analyzed the specimens completely blinded to patient responder status; thus, by design, all patient specimens were randomized during processing and analysis.

Blinding

Tumor microarray staining (Fig. 1i-k) and analysis was performed blinded, until the final analysis, whereupon the biostatisticians, who are not part of the Lau laboratory, were unblinded to tissue donor sex, age, and stage.

Patient tumor biopsy staining (Fig. 4c-e): We obtained blinded patient specimens from Moffitt, MD Anderson, and Harvard/Massachusetts General Hospital. Staining and analysis was performed blinded, until the final analysis, whereupon the biostatisticians, who are not part of the Lau laboratory, were unblinded to responder patient responder status.

The investigators were completely blinded to group allocation during data collection and all analyses.

Reporting for specific materials, systems and methods

We require information from authors about some types of materials, experimental systems and methods used in many studies. Here, indicate whether each material, system or method listed is relevant to your study. If you are not sure if a list item applies to your research, read the appropriate section before selecting a response.

Materials & experimental systems

n/a	Involved in the study
☐	☒ Antibodies
☐	☒ Eukaryotic cell lines
☒	☐ Palaeontology and archaeology
☐	☒ Animals and other organisms
☐	☒ Clinical data
☒	☐ Dual use research of concern

Methods

n/a	Involved in the study
☒	☐ ChIP-seq
☐	☒ Flow cytometry
☒	☐ MRI-based neuroimaging

Antibodies

Antibodies used

Mouse anti-V5 (Cat. #: v8012; final use concentration: 0.2 µg/mL; Clone #V5-10; Lot #00000999991; Millipore Sigma (St. Louis, MO))
•Website: https://www.sigmaaldrich.com/US/en/product/sigma/v8012
•Associated Figure(s): Fig 2c & 3c, extended data fig 6a-d

Mouse anti-V5 gel (Cat. #:a7345; Clone # V5-10; Lot #119M4791V; Millipore Sigma (St. Louis, MO))
•Website: https://www.sigmaaldrich.com/US/en/product/sigma/a7345?context=product
•Associated Figure(s): Fig 2c, extended data fig 6a-d

Mouse anti-human HLA-DRB1 (Cat. #:ab215835; final use concentration: 0.2 µg/mL for IF; Clone #HLA-DRB/1067; Lot# NA; Abcam (Cambridge, UK))
•Website: https://www.abcam.com/hla-class-ii-drb1-antibody-hla-drb1067-ab215835.html
•Associated Figure(s): Fig 3d, Fig 5b-f, Extended Data Fig 7b-e

Rabbit anti-human HLA-DRB1 (Cat. #:ab92371; final use concentration: 0.2 µg/mL for WB; Polyclonal; Lot #GR922220-2; Abcam (Cambridge, UK))
•Website: https://www.abcam.com/hla-class-ii-drb1-antibody-ab92371.html
•Associated Figure(s): Fig. 2a-e, Fig 3e, Extended Fig 3b

Mouse anti-[illegible]-tubulin (Cat. #: E7; final use concentration: 0.3 µg/mL; clone# E7; Lot# 11/29/18; developed by M. McCutcheon and S. Carroll and obtained from Developmental Studies Hybridoma Bank (University of Iowa, Iowa City, IA))
•Website: https://dshb.biology.uiowa.edu/E7_2
•Associated Figure(s): Fig 2a, Fig 3c,e, Extended Data Fig 1l, Extended Data Fig 5b, Extended Data Fig 6e

Goat anti-biotin (Cat. #: sp3000; final use concentration: 0.1 µg/mL; polyclonal; Lot# ZH0202; Vector Labs (Burlingame, CA))
•Website: https://vectorlabs.com/goat-anti-biotin-unconjugated.html#documents
•Associated Figure(s): Fig 5b-f, Extended Data Fig 7b-h

Anti-mouse CD4 (Cat. #: BE0003-1; Dosage: 20 mg/kg for immunodepletion; Clone# GK1.5; Lot# 689518A1; Bioxcell (West Lebanon, NH))
•Website: https://bxcell.com/product/m-cd4/
Manufacturer verified for use: immunodepletion, flow cytometry, western blot
•Associated Figure(s): Fig 1n-o, Extended Data Fig 1 n,p,q,u,v,w

Anti-mouse CD8 (Cat. #: BE0061; Dosage: 20 mg/kg for immunodepletion; Clone# 2.43; Lot #: 643017F1; Bioxcell (West Lebanon, NH))
•Website: https://bxcell.com/product/m-cd8a-2/
•Associated Figure(s): Fig 1m, Extended Data 1o,r,t

Anti-mouse IgGk horseradish peroxidase (HRP) (Cat. #: SC-516102; final use concentration: 0.04 µg/mL; Clone: NA; Lot#F1620; Santa Cruz Biotechnology (Dallas, TX))
•Website: https://www.scbt.com/p/m-igg-kappa-bp-hrp
•Associated Figure(s): Fig 2a-c, Fig 3c, Extended Data Fig 1l, Extended Data Fig 5b

Mouse anti-rabbit HRP (Cat. #: SC-2357; final use concentration: 0.04 µg/mL; Clone# NA; Lot# G0720; Santa Cruz Biotechnology (Dallas, TX))
•Website: https://www.scbt.com/p/mouse-anti-rabbit-igg-hrp
•Associated Figure(s): Fig 2a-c, Fig 3c, Extended Data Fig 1l, Extended Data Fig 5b

Goat anti-rabbit AlexaFluor 488 (Cat. #: A11008; final use concentration: 0.04 µg/mL; Clone # NA; Lot #1275894; ThermoFisher Scientific (Waltham, MA))
•Website: https://www.thermofisher.com/antibody/product/Goat-anti-Rabbit-IgG-H-L-Highly-Cross-Adsorbed-Secondary-Antibody-Polyclonal/A32731
•Associated Figure(s): Fig 3e, Extended Data 5b

Donkey anti-mouse AlexaFluor 594 (Cat. #: A21203; final use concentration: 0.05 µg/mL; Clone #NA; Lot #1918277; ThermoFisher Scientific (Waltham, MA))
•Website: https://www.thermofisher.com/antibody/product/Donkey-anti-Mouse-IgG-H-L-Highly-Cross-Adsorbed-Secondary-Antibody-Polyclonal/A32744
•Associated Figure(s): Fig 1i-k

AlexaFluor 594 donkey anti-rabbit (Cat. #: A21207; final use concentration: 0.05 µg/mL; Clone #NA; Lot #1938375; ThermoFisher Scientific (Waltham, MA))
•Website: https://www.thermofisher.com/antibody/product/Donkey-anti-Rabbit-IgG-H-L-Highly-Cross-Adsorbed-Secondary-Antibody-Polyclonal/A-21207
•Associated Figure(s): Fig 3d,

Rabbit anti-Mart1 (Cat. #: SAB4500949; final use concentration: 0.2 µg/mL; Clone #NA; Lot #310257; Millipore Sigma (St. Louis, MO)
•Website: https://www.sigmaaldrich.com/US/en/product/sigma/sab4500949?context=product
•Associated Figure(s): Fig 1i-k, Fig 5c-f, Extended Data Fig 1a,d,l, Extended Data Fig 4 b-h

Rabbit anti-S100 (Cat. #: Z0311; final use concentration: 0.2 µg/mL; Clone #NA; Lot #NA; Agilent Technologies (Santa Clara, CA))
•Website: https://www.agilent.com/en/product/immunohistochemistry/antibodies-controls/primary-antibodies/s100-(dako-omnis)-76198
•Associated Figure(s): Fig 1i-k, Fig 5c-f, Extended Data Fig 1a,d,l, Extended Data Fig 7 b-h

Rat APC anti-mouse CD3 (Cat. #: 100236; final use concentration: 0.5 µg/mL; Clone #17A2; Lot #B274725; Biolegend, inc. (San Diego, CA))
•Website: https://www.biolegend.com/en-us/products/apc-anti-mouse-cd3-antibody-8055
•Associated Figure(s): Fig 1c,d,g,h,o, Fig 2h, Extended Data Fig 1b,c,g,h,i,j,m,n,q,w, Extended Data Fig 3e

Rat Pacific Blue anti-mouse CD4 (Cat. #: 100428; final use concentration: 0.5 µg/mL; Clone #GK1.5; Lot #B265546; Biolegend, Inc. (San Diego, CA))
•Website:https://www.biolegend.com/en-us/products/pacific-blue-anti-mouse-cd4-antibody-3316?GroupID=BLG4745
•Associated Figure(s): Fig 1d,h,o, Fig 2h, Extended Data Fig 1j,n,q,w, Extended Data Fig 3e

Rat BV785 anti-mouse CD8 (Cat. #: 100750; final use concentration: 0.5 µg/mL; Clone #53-6.7; Lot #B266841; Biolegend (San Diego, CA))
•Website: https://www.biolegend.com/en-us/products/brilliant-violet-785-anti-mouse-cd8a-antibody-7957
•Associated Figure(s): Fig 1d,h,o, Fig 2h, Extended Data Fig 1j,w, Extended Data Fig 3e

Rat FITC anti-mouse F4/80 (Cat. #: 123108; final use concentration: 0.5 µg/mL; Clone #BM8; Lot #B257637; Biolegend, Inc (San Diego, CA))
•Website: https://www.biolegend.com/en-us/products/fitc-anti-mouse-f4-80-antibody-4067
•Associated Figure(s): Fig 1c,g, Extended Data Fig 1b,c,g,h,i,m

Rat APC anti-mouse GR-1 (Cat. #: 108412; final use concentration: 0.5 µg/mL; Clone #RB6-8C5; Lot #B288472; Biolegend, Inc. (San Diego, CA))
•Website: https://www.biolegend.com/en-us/products/apc-anti-mouse-ly-6g-ly-6c-gr-1-antibody-456
Manufacturer verified for use: flow cytometry
•Associated Figure(s): Fig 1c, Extended Data Fig 1b,c,g,i

Armenian Hamster PeCy7 anti-mouse CD11c (Cat. #: 117318; final use concentration: 0.5 µg/mL; Clone #N418; Lot #B269973; Biolegend, Inc. (San Diego, CA))
•Website: https://www.biolegend.com/en-us/products/pe-cyanine7-anti-mouse-cd11c-antibody-3086
•Associated Figure(s): Fig 1c,g,o, Fig 2h, Extended Data Fig 1b,c,g,l,m,w

Mouse PE anti-mouse NK1.1 (Cat. #: 156504; final use concentration: 0.5 µg/mL; Clone #PK136; Lot #B277026; Biolegend, Inc. (San Diego, CA))
•Website: https://www.biolegend.com/en-us/products/pe-anti-mouse-nk-1-1-antibody-16926
•Associated Figure(s): Extended Data Fig 1g,h,i,j,w

Rat PE anti-mouse CD49b (Cat. #: 108908; final use concentration: 0.5 µg/mL; Clone #DX5; Lot #4289830; Biolegend, Inc. (San Diego, CA))
•Website: https://www.biolegend.com/en-us/products/pe-anti-mouse-cd49b-pan-nk-cells-antibody-234
•Associated Figure(s): Fig 1c,g,o, Extended Data Fig 1b,c,m,w

Rat PerCP-Cy5.5 anti-mouse CD11b (Cat. #: 101228; final use concentration: 0.5 µg/mL; Clone #M1/70; Lot #B256661; Biolegend, Inc. (San Diego, CA))
•Website: https://www.biolegend.com/en-us/products/percp-cyanine5-5-anti-mouse-human-cd11b-antibody-4257
•Associated Figure(s): Fig 1c,g,o, Fig 2h, Extended Data Fig 1b,c,g,l,m,w

Rabbit anti-human PD-L1 (Cat. #: NBP1-76769; final use concentration: 0.5 µg/mL; clone #NBP1-76769; Lot #8293-1701; Novus Biologicals, Centennial, CO)
•Website: https://www.novusbio.com/products/pd-l1-antibody_nbp1-76769
•Associated Figure(s): Extended Data Fig. 7i

Rat PE anti-mouse PD-L1 (Cat. #: 124308; final use concentration: 0.5 µg/mL; clone #10F.9G2; Lot #B278301; Biolegend, (San Diego, CA))
•Website: https://www.biolegend.com/en-us/products/pe-anti-mouse-cd274-b7-h1-pd-l1-antibody-4497
•Associated Figure(s): Extended Data Fig. 7i

Phalloidin Alexafluor 488 (Cat. #: A12379; final use concentration: 0.2µg/mL; clone #NA; Lot #1737901; ThermoFisher Scientific (Waltham, MA))
•Website: https://www.thermofisher.com/order/catalog/product/A12379?ef_id=CjwKCAjwieuGBhAsEiwA1Ly_nbHglCspHMlFqajO6O2qiH_nv_inLTLvWwYXUfQUMh1OCprNzU7-

VBoCUI4QAvD_BwE:G:s&s_kwcid=AL!3652!3!447292198730!b!!g!!
&cid=bid_pca_iva_r01_co_cp1359_pjt0000_bid00000_0se_gaw_dy_pur_con&gclid=CjwKCAjwieuGBhAsEiwA1Ly_nbHglCspHMlFqaj O6O2qiH_nv_inLTLvWwYXUfQUMh1OCprNzU7-VBoCUI4QAvD_BwE#/A12379?
ef_id=CjwKCAjwieuGBhAsEiwA1Ly_nbHglCspHMlFqajO6O2qiH_nv_inLTLvWwYXUfQUMh1OCprNzU7-
VBoCUI4QAvD_BwE:G:s&s_kwcid=AL!3652!3!447292198730!b!!g!!
&cid=bid_pca_iva_r01_co_cp1359_pjt0000_bid00000_0se_gaw_dy_pur_con&gclid=CjwKCAjwieuGBhAsEiwA1Ly_nbHglCspHMlFqaj O6O2qiH_nv_inLTLvWwYXUfQUMh1OCprNzU7-VBoCUI4QAvD_BwE
•Associated Figure(s): Extended Data Fig. 7c

Mouse anti-FLAG (Cat. #: F1804; final use concentration: 0.2 µg/mL; clone #M2; Lot #SLBQ7119V; Millipore Sigma (St. Louis, MO))
•Website: https://www.sigmaaldrich.com/US/en/substance/monoclonalantiflagm2antibodyproducedinmouse1234598765?context=product
•Associated Figure(s): Extended Data Fig. 1i, extended data 6c

Fabbit polyclonal anti-HLA-A (Cat. #: 15240-1-AP; final use concentration: 0.2 µg/mL; clone # NA; Lot #00006298; Proteintech (Rosemont, IL))
•Website: https://www.ptglab.com/products/HLA-A-Antibody-15240-1-AP.htm
•Associated Figure(s): Fig. 2a-c

Normal mouse IgG (Cat. #: SC-2025; final use concentration: NA; clone # NA; Lot #B0619; Santa Cruz Biotechnology (Dallas, TX))
•Website: https://www.scbt.com/p/normal-mouse-igg
•Associated Figure(s): Fig 2c

Rabbit anti-KDEL (Cat. #: PA1-013; final use concentration: 0.1 µg/mL; clone # JB42-04; Lot # VG3025834; ThermoFisher Scientific (Waltham, MA))
•Website: https://www.thermofisher.com/antibody/product/KDEL-Antibody-clone-JB42-04-Recombinant-Monoclonal/MA5-34715
•Associated Figure(s): Fig. 3d

Mouse anti-PD1 (Cat. #: BE-0146; Dosage 20 mg/kg; clone# RMP1-14; Lot# 780120S1C; Bioxcell (West Lebanon, NH))
•Website: https://bxcell.com/product/invivomab-anti-m-pd-1/
•Associated Figure(s): Fig 4a & b

Donkey anti-goat plus PLA secondary antibody (Cat. #: DUO-92003; final use concentration: NA; clone# NA; Lot# SLBZ8370; Millipore Sigma (St. Louis, MO))
•Website: https://www.sigmaaldrich.com/US/en/product/sigma/duo92003?context=product
•Associated Figure(s): Fig. 5b-5f, Extended Data Fig. 7ch

Donkey anti-mouse minus PLA secondary antibody (Cat. #: DUO-92004; final use concentration: NA clone# NA; Lot# SLCC8780; Millipore Sigma (St. Louis, MO))
•Website: https://www.sigmaaldrich.com/US/en/product/sigma/duo92004?context=product
•Associated Figure(s): Fig. 5b-5f, Extended Data Fig. 7cf

Rat anti-mouse CD8 (Cat. #: 550-281; final use concentration: 0.2 µg/mL; Clone# 53-6.7; Lot #5338976; BD Pharmingen (San Jose, CA))
•Website: https://www.bdbiosciences.com/en-us/products/reagents/flow-cytometry-reagents/research-reagents/single-color-antibodies-ruo/purified-rat-anti-mouse-cd8a.550281
•Associated Figure(s): Extended Data Fig. 1o, 1r

AlexaFluor 594 goat anti-rat secondary antibody (Cat. #: A11007; final use concentration: 0.05 µg/mL; Clone# NA; Lot# 1697164; ThermoFisher Scientific (Waltham, MA))
•Website: https://www.thermofisher.com/antibody/product/Goat-anti-Rat-IgG-H-L-Cross-Adsorbed-Secondary-Antibody-Polyclonal/A-11007
•Associated Figure(s): Extended Data Fig. 1o, 1r

Mouse anti-CD3 (Cat. #: SC-59013; final use concentration: 0.2 µg/mL; Clone# PS1; Lot # G2419; Santa Cruz Biotechnology (Dallas, TX)
•Website: https://www.scbt.com/p/cd3-antibody-ps1
•Associated Figure(s): Fig. 1i-1k

Mouse PE anti-pan-MHC-I (HLA-A,B,C) (Cat. #: 555553; Clone #: G46-2.6; final use dilution: 1:10; Lot #: multiple; BD Pharmingen (San Jose, CA))
•Website: https://www.bdbiosciences.com/en-us/products/reagents/flow-cytometry-reagents/research-reagents/single-color-antibodies-ruo/pe-mouse-anti-human-hla-abc.555553
•Associated Figure(s): Extended Data Fig. 6h

Mouse FITC anti-pan-MHC-II (HLA-DP, DQ, DQ)(Cat. #: 555558; Clone #: Tu39; Lot #: multiple; final use dilution: 1:10; BD Pharmingen (San Jose, CA))
•Website: https://www.bdbiosciences.com/en-us/products/reagents/flow-cytometry-reagents/research-reagents/single-color-antibodies-ruo/fitc-mouse-anti-human-hla-dr-dp-dq.555558
•Associated Figure(s): Extended Data Fig. 6h

Mouse PerPCy5.5 anti-CD45 (Cat. #: 45-9459-42; Clone #: 2D1; Lot #: multiple; final use dilution: 1:20; Invitrogen (Waltham, MA))
•Website: https://www.thermofisher.com/antibody/product/CD45-Antibody-clone-2D1-Monoclonal/45-9459-42
•Associated Figure(s): Extended Data Fig. 6h

Mouse APC anti-CD90 (Cat. #: 328114; Clone #: 5E10; Lot #: multiple; final use dilution: 1:20; Biolegend (San Diego, CA))
•Website: https://www.biolegend.com/en-us/products/apc-anti-human-cd90-thy1-antibody-4116

•Associated Figure(s): Extended Data Fig. 6h

Mouse BV421 anti EpCAM (Cat. #: 324220; Clone #: 9C4; Lot #: multiple; final use dilution: 1:20; Biolegend (San Diego, CA)).
•Website: https://www.biolegend.com/en-us/products/brilliant-violet-421-anti-human-cd326-epcam-antibody-7549
•Associated Figure(s): Extended Data Fig. 6h

Biotinylated AAL (Cat. #: B-1395; final use concentration: 0.4 μg/mL; Lot #ZH0504; Vector Labs, Burlingame, CA)
•Website: https://vectorlabs.com/biotinylated-aleuria-aurantia-lectin-aal.html
•Associated Figure(s): Fig 2c, Fig 3e, Fig 5b-e, Extended Data 5b, Extended Data Fig 7c-f

Fluorescein-conjugated AAL (Cat. #: FL-1391; final use concentration: 0.4 μg/mL; Lot# ZF0326; Vector Laboratories, Burlingame, CA)
•Website: https://vectorlabs.com/fluorescein-labeled-aleuria-aurantia-lectin-aal.html
•Associated Figure(s): Fig 1i-k, Fig 3e, Fig 5b-e, Extended Data 1a,d,l, Extended Data 5b, Extended data 7c-f

Agarose UEA1 and AAL (Cat. #: AL-1393 and AL-1063-2, respectively; Lot# ZG0918 for AAL; Vector Laboratories, (Burlingame, CA))
•Website: https://vectorlabs.com/agarose-bound-aleuria-aurantia-lectin-aal.html https://vectorlabs.com/agarose-bound-ulex-europaeus-agglutinin-i-uea-i.html
•Associated Figure(s): Fig 2b, Fig 3c, Extended Data 6e

Fixable Live/Dead stain (Biolegend (San Jose, CA)
•Website:https://www.biolegend.com/en-us/products/zombie-nir-fixable-viability-kit-8657?GroupID=BLG2181
•Associated Figure(s): Fig. 1b-d, 1f-h, 1o, 2g,h, Extended Data Fig. 1b,c,f,g,h,l,j,m,n,p,q,v,w, 3d,e, 4a

PKH26 Millipore Sigma (Cat. #: 555553; St. Louis, MO))
•Website:https://www.sigmaaldrich.com/US/en/product/sigma/pkh26gl?gclid=CjwKCAjwieuGBhAsEiwA1Ly_nQjfjFZr31KPneW6vzdP_OXJyufdHtdDgQD5n88STPgkzO-GumvM8hoCESoQAvD_BwE
•Associated Figure(s): Fig. 3e, Extended Fig. 5b, 7i

Alexa Fluor 647 anti-human/mouse Granzyme B Antibody (Cat. #: 515406; Lot # B266742; Concentration: 0.5 μg/mL; Biolegend (San Diego, CA))
Website: https://www.biolegend.com/en-us/products/alexa-fluor-647-anti-human-mouse-granzyme-b-antibody-6067?GroupID=BLG15670
Associated Figures: Extended Data Fig 3d & e

BUV805 Rat Anti-Mouse CD8a (Cat. # 612898; Lot # 1194016; Concentration: 0.5 μg/mL; BD Biosciences (Franklin Lakes, NJ))
Website: https://www.bdbiosciences.com/en-us/products/reagents/flow-cytometry-reagents/research-reagents/single-color-antibodies-ruo/buv805-rat-anti-mouse-cd8a.612898
Associated Figures: Extended Data Fig 3c-e, Fig. 4b

BV421 Rat Anti-Mouse I-A/I-E (Cat.# 562564 Lot # 1074223; Concentration: 0.5 μg/mL; BD Biosciences (Franklin Lakes, NJ))
Website: https://www.bdbiosciences.com/en-us/products/reagents/flow-cytometry-reagents/research-reagents/single-color-antibodies-ruo/bv421-rat-anti-mouse-i-a-i-e.562564
Associated Figures: Extended Data Fig 3c, Fig. 4b

Live/Dead Aqua (Cat# L34965; Lot #: multiple; ThermoFisher Scientific (Waltham, MA))
Website:https://www.thermofisher.com/order/catalog/product/L34957?s_kwcid=AL!3652!3!358452930868!e!!g!!live%20dead%20aqua&ef_id=CjwKCAjw8sCRBhA6EiwA6_IF4YuAc427TmvCQ_pkCYMPlc2WPRiJJVnVdniFZLRrqcfT57IgVnt6uxoCCaQQAvD_BwE:G:s&s_kwcid=AL!3652!3!358452930868!e!!g!!live%20dead%20aqua&cid=bid_pca_frg_r01_co_cp1359_pjt0000_bid00000_0se_gaw_bt_pur_con&gclid=CjwKCAjw8sCRBhA6EiwA6_IF4YuAc427TmvCQ_pkCYMPlc2WPRiJJVnVdniFZLRrqcfT57IgVnt6uxoCCaQQAvD_BwE
Associated Figures: Extended Data Fig 3c-e, Fig. 4b

BV605 anti-mouse CD11c (Cat. #:117334; Lot # B342521; Concentration: 0.5 μg/mL; Biolegend (San Diego, CA))
Website: https://www.biolegend.com/en-us/products/brilliant-violet-605-anti-mouse-cd11c-antibody-7865?GroupID=BLG11937
Associated Figures: Extended Data Fig 3c, Fig. 4b

BV711 Rat anti-mouse CD11b (Cat.# 563168; Lot # 1116158; Concentration: 0.5 μg/mL; BD Biosciences (Franklin Lakes, NJ))
Website: https://www.bdbiosciences.com/en-us/products/reagents/flow-cytometry-reagents/research-reagents/single-color-antibodies-ruo/bv711-rat-anti-cd11b.563168
Associated Figures: Extended Data Fig 3c-e, Fig 4b

BV421 Rat anti-mouse CD44 (Cat.# 563970; Lot #0337154; Concentration: 0.5 μg/mL; BD Biosciences (Franklin Lakes, NJ))
Website: https://www.bdbiosciences.com/en-us/products/reagents/flow-cytometry-reagents/research-reagents/single-color-antibodies-ruo/bv421-rat-anti-mouse-cd44.563970
Associated Figures: Extended Data Fig 3c, Fig 4b

BV650 Rat anti-mouse CD4 (Cat.# 563747; Lot #1013120; Concentration: 0.5 μg/mL; BD Biosciences (Franklin Lakes, NJ))
Website: https://www.bdbiosciences.com/en-us/products/reagents/flow-cytometry-reagents/research-reagents/single-color-antibodies-ruo/bv650-rat-anti-mouse-cd4.563747
Associated Figures: Extended Data Fig 3c-e, Fig 4b

BV785 Rat anti-mouse CD45 (Cat.# 564225; Lot #1175935; Concentration: 0.5 μg/mL; BD Biosciences (Franklin Lakes, NJ))
Website: https://www.bdbiosciences.com/en-us/products/reagents/flow-cytometry-reagents/research-reagents/single-color-antibodies-ruo/bv786-rat-anti-mouse-cd45.564225
Associated Figures: Extended Data Fig 3c-e, Fig 4b

APC anti-mouse F4/80 (Cat. #: 123116; Lot # B298926; Concentration: 0.5 µg/mL; Biolegend (San Diego, CA))
Website: https://www.biolegend.com/en-gb/products/apc-anti-mouse-f4-80-antibody-4071?GroupID=BLG5319
Associated Figures: Extended Data Fig 3c, Fig 4b

PeCy anti-mouse CD103 (Cat. #: 121426; Lot # B283947; Concentration: 0.5 µg/mL; Biolegend (San Diego, CA))
Website: https://www.biolegend.com/en-us/products/pe-cyanine7-anti-mouse-cd103-antibody-9899?GroupID=BLG7093
Associated Figures: Extended Data Fig 3c, Fig 4b

BUV Rat anti-mouse CD62L (Cat.# 612833; Lot #111748; Concentration: 0.5 µg/mL; BD Biosciences, (Franklin Lakes, NJ))
Website: https://www.bdbiosciences.com/en-us/products/reagents/flow-cytometry-reagents/research-reagents/single-color-antibodies/buv737-rat-anti-mouse-cd62l.612833
Associated Figures: Extended Data Fig 3c, Fig 4b

PeCy7 anti-mouse CRTAM (Cat. #: 142013; Lot # B285616; Concentration: 0.5 µg/mL; Biolegend, (San Diego, CA))
Website: https://www.biolegend.com/en-us/products/pe-cyanine7-anti-mouse-cd355-crtam-antibody-15073?GroupID=BLG9494
Associated Figures: Extended Data Fig 3d-e, Fig 4b

APC-R700 hamster anti-mouse CD95 (Cat.# 565130; Lot #1057239; Concentration: 0.5 µg/mL; BD Biosciences (Franklin Lakes, NJ))
Website: https://www.bdbiosciences.com/en-us/products/reagents/flow-cytometry-reagents/research-reagents/single-color-antibodies-ruo/apc-r700-hamster-anti-mouse-cd95.565130
Associated Figures: Extended Data Fig 3c, Fig 4b

PE-Dazzle Hamster anti-mouse FC[illegible]R1 (Cat. #: 139319; Lot # B343474; Concentration: 0.5 µg/mL; Biolegend (San Diego, CA))
Website: https://www.biolegend.com/en-us/products/pe-dazzle-594-anti-mouse-fcepsilonrialpha-antibody-14527?GroupID=BLG6716
Associated Figures: Extended Data Fig 3c, Fig 4b

Rabbit Calnexin (C5C9) R1 (Cat. #: 2679T; Lot # 4; Concentration: 1/1000; Cell Signaling Technology (Danvers, MA))
Website: https://www.cellsignal.com/products/primary-antibodies/calnexin-c5c9-rabbit-mab/2679
Associated Figures: Extended Data Fig 6d
Finglimod (hydrochloride) (FTY720) (Cat. #: 10006292; Lot # 0572050-040; Concentration: 20 µg per mouse every 2 days; Cayman Chemical (Ann Arbor MI))
Website: https://www.caymanchem.com/product/10006292/fingolimod-(hydrochloride)
Associated Figures: Extended Data Fig 3b-e

Goat anti-Integrin [illegible]5 (Cat#: ab31327; Lot# GR68472-8; Concentration: 0.5 µg/mL; Abcam (Cambridge, UK)))
Website: https://www.abcam.com/integrin-beta-5-antibody-ab31327.html
Associated Figures: Extended Data Fig 2e

Human Easy Sep CD4 isolation negative selection cocktail (Cat#: 17952; Lot# 10000072656; Stem Cell Technologies (Vancouver, Canada))
Website: https://www.stemcell.com/easysep-human-cd4-t-cell-isolation-kit.html
Associated Figures: Extended Data Fig 2a-e

Ficoll-Paque PLUS (Cat# 17144002; Lot# 10305896; Cytiva (Marlborough, MA))
Website: https://www.cytivalifesciences.com/en/us/shop/cell-therapy/media/ficoll-paque-plus-density-gradient-media-p-05824
Associated Figures: Extended Data Fig 2a-e

CD3/CD28 human dynabeads (Cat# 11131D; Lot# Multiple; Thermofisher Scientific (Waltham, MA))
Website: https://www.thermofisher.com/order/catalog/product/11131D
Associated Figures: Extended Data Fig 2a-e

Phospho-PKA Substrate (RRXS*/T*) (Cat. #: 9624S; Lot # 21; Concentration: 1/1000; Cell Signaling (Danvers, MA))
Website: https://www.cellsignal.com/products/primary-antibodies/phospho-pka-substrate-rrxs-t-100g7e-rabbit-mab/9624#:~:text=Phospho%2DPKA%20substrate%2Dspecific%20antibodies,high%20throughput%20kinase%20drug%20discovery.
Associated Figures: Extended Data Fig 2b

Ponceau Stain, Proteomics Grade (Cat. #: 97063-650; Lot # 20J1356566; VWR (Radnor, PA))
Website: https://us.vwr.com/store/product/7437354/ponceau-s-proteomics-grade
Associated Figures: Extended Data Fig 2b-c

Validation

Mouse anti-V5 (Cat. #: v8012; final use concentration: 0.2 µg/mL; Clone #V5-10; Lot #00000999991; Millipore Sigma (St. Louis, MO))
•Website https://www.sigmaaldrich.com/US/en/product/sigma/v8012
•Manufacturer verified for use: immunocytochemistry, western blot
•Relevant Reference(s): Ortuno D, Carlisle HJ, Miller S. Does inactivation of USP14 enhance degradation of proteasomal substrates that are associated with neurodegenerative diseases? F1000Res. 2016 Feb 4;5:137. doi: 10.12688/f1000research.7800.2. PMID: 26998235; PMCID: PMC4792207.
Ciabatti E, González-Rueda A, Mariotti L, Morgese F, Tripodi M. Life-Long Genetic and Functional Access to Neural Circuits Using Self-Inactivating Rabies Virus. Cell. 2017 Jul 13;170(2):382-392.e14. doi: 10.1016/j.cell.2017.06.014. Epub 2017 Jul 6. PMID: 28689641; PMCID: PMC5509544
•Associated Figure(s): Fig 2c & 3c, Extended data fig 6a-d

Mouse anti-V5 gel (Cat. #:a7345; Clone # V5-10; Lot #119M4791V; Millipore Sigma (St. Louis, MO))
•Website https://www.sigmaaldrich.com/US/en/product/sigma/a7345?context=product
•Manufacturer verified for use: affinity chromatography, immunoprecipitation

•Relevant Reference(s): Meng Q, Li M, Silberg MA, Conrad F, Bettencourt J, To R, Huang C, Ma J, Meyer K, Shimizu R, Cao L, Tomic MT, Marks JD. Domain-based assays of individual antibody concentrations in an oligoclonal combination targeting a single protein. Anal Biochem. 2012 Feb 15;421(2):351-61. doi: 10.1016/j.ab.2011.09.030. Epub 2011 Oct 6. PMID: 22037290; PMCID: PMC4209596.
Dasgupta A, Chen CH, Lee C, Gladfelter AS, Dunlap JC, Loros JJ. Biological Significance of Photoreceptor Photocycle Length: VIVID Photocycle Governs the Dynamic VIVID-White Collar Complex Pool Mediating Photo-adaptation and Response to Changes in Light Intensity. PLoS Genet. 2015 May 15;11(5):e1005215. doi: 10.1371/journal.pgen.1005215. PMID: 25978382; PMCID: PMC4433212
•Associated Figure(s): Fig 2c, Extended data fig 6a-d

Mouse anti-human HLA-DRB1 (Cat. #:ab215835; final use concentration: 0.2 µg/mL for IF; Clone #HLA-DRB/1067; Lot# NA; Abcam (Cambridge, UK))
•Website https://www.abcam.com/hla-class-ii-drb1-antibody-hla-drb1067-ab215835.html
•Manufacturer verified for use: immunohistochemistry, western blot, flow cytometry, immunocytochemistry
•Relevant Reference(s): NA
•Associated Figure(s): Fig 3d, Fig 5b-f, Extended Data Fig 7b-e

Rabbit anti-human HLA-DRB1 (Cat. #:ab92371; final use concentration: 0.2 µg/mL for WB; Polyclonal; Lot #GR922220-2; Abcam (Cambridge, UK))
•Website https://www.abcam.com/hla-class-ii-drb1-antibody-ab92371.html
•Manufacturer verified for use: western blot, ELISA
•Relevant Reference(s): NA
•Associated Figure(s): Fig. 2a-e, Fig 3e, Extended Fig 3b

Mouse anti-beta-tubulin (Cat. #: E7; final use concentration: 0.3 µg/mL; clone# E7; Lot# 11/29/18; developed by M. McCutcheon and S. Carroll and obtained from Developmental Studies Hybridoma Bank (University of Iowa, Iowa City, IA))
•Website https://dshb.biology.uiowa.edu/E7_2
•Manufacturer verified for use: immunofluorescence, immunohistochemistry, immunoprecipitation, western blot
•Relevant Reference(s): Chu DT, Klymkowsky MW. The appearance of acetylated alpha-tubulin during early development and cellular differentiation in Xenopus. Dev Biol. 1989 Nov;136(1):104-17. doi: 10.1016/0012-1606(89)90134-6. PMID: 2680681
Monyak RE, Emerson D, Schoenfeld BP, Zheng X, Chambers DB, Rosenfelt C, Langer S, Hinchey P, Choi CH, McDonald TV, Bolduc FV, Sehgal A, McBride SMJ, Jongens TA. Insulin signaling misregulation underlies circadian and cognitive deficits in a Drosophila fragile X model. Mol Psychiatry. 2017 Aug;22(8):1140-1148. doi: 10.1038/mp.2016.51. Epub 2016 Apr 19. PMID: 27090306; PMCID: PMC5071102
•Associated Figure(s): Fig 2a, Fig 3c,e, Extended Data Fig 1l, Extended Data Fig 5b, Extended Data Fig 6e

Goat anti-biotin (Cat. #: sp3000; final use concentration: 0.1 µg/mL; polyclonal; Lot# ZH0202; Vector Labs (Burlingame, CA))
•Website https://vectorlabs.com/goat-anti-biotin-unconjugated.html#documents
•Manufacturer verified for use: immunohistochemistry, immunofluorescence, in situ hybridization, blotting, ELISPOT, ELISA
•Relevant Reference(s): Mosialou I, Shikhel S, Liu JM, Maurizi A, Luo N, He Z, Huang Y, Zong H, Friedman RA, Barasch J, Lanzano P, Deng L, Leibel RL, Rubin M, Nickolas T, Chung W, Zeltser LM, Williams KW, Pessin JE, Kousteni S. MC4R-dependent suppression of appetite by bone-derived lipocalin 2. Nature. 2017 Mar 16;543(7645):385-390. doi: 10.1038/nature21697. Epub 2017 Mar 8. Erratum in: Nature. 2017 Jun 14;546(7658):440. PMID: 28273060; PMCID: PMC5975642
Cholanian M, Krajewski-Hall SJ, McMullen NT, Rance NE. Chronic oestradiol reduces the dendritic spine density of KNDy (kisspeptin/neurokinin B/dynorphin) neurones in the arcuate nucleus of ovariectomised Tac2-enhanced green fluorescent protein transgenic mice. J Neuroendocrinol. 2015 Apr;27(4):253-63. doi: 10.1111/jne.12263. PMID: 25659412; PMCID: PMC4788980
•Associated Figure(s): Fig 5b-f, Extended Data Fig 7b-h

Anti-mouse CD4 (Cat. #: BE0003-1; Dosage: 20 mg/kg for immunodepletion; Clone# GK1.5; Lot# 689518A1; Bioxcell (West Lebanon, NH))
•Website https://bxcell.com/product/m-cd4/
•Manufacturer verified for use: immunodepletion, flow cytometry, western blot
•Relevant Reference(s): Balogh KN, Templeton DJ, Cross JV. Macrophage Migration Inhibitory Factor protects cancer cells from immunogenic cell death and impairs anti-tumor immune responses. PLoS One. 2018 Jun 4;13(6):e0197702. doi: 10.1371/journal.pone.0197702. PMID: 29864117; PMCID: PMC5986154.
Budda SA, Zenewicz LA. IL-22 deficiency increases CD4 T cell responses to mucosal immunization. Vaccine. 2018 Jun 14;36(25):3694-3700. doi: 10.1016/j.vaccine.2018.05.011. Epub 2018 May 5. PMID: 29739717
•Associated Figure(s): Fig 1n-o, Extended Data Fig 1 n,p,q,u,v,w

Anti-mouse CD8 (Cat. #: BE0061; Dosage: 20 mg/kg for immunodepletion; Clone# 2.43; Lot #: 643017F1; Bioxcell (West Lebanon, NH))
•Website https://bxcell.com/product/m-cd8a-2/
•Manufacturer verified for use: immunodepletion, western blot
•Relevant Reference(s): Balogh KN, Templeton DJ, Cross JV. Macrophage Migration Inhibitory Factor protects cancer cells from immunogenic cell death and impairs anti-tumor immune responses. PLoS One. 2018 Jun 4;13(6):e0197702. doi: 10.1371/journal.pone.0197702. PMID: 29864117; PMCID: PMC5986154.
Li J, Lee Y, Li Y, Jiang Y, Lu H, Zang W, Zhao X, Liu L, Chen Y, Tan H, Yang Z, Zhang MQ, Mak TW, Ni L, Dong C. Co-inhibitory Molecule B7 Superfamily Member 1 Expressed by Tumor-Infiltrating Myeloid Cells Induces Dysfunction of Anti-tumor CD8+ T Cells. Immunity. 2018 Apr 17;48(4):773-786.e5. doi: 10.1016/j.immuni.2018.03.018. Epub 2018 Apr 3. PMID: 29625896
•Associated Figure(s): Fig 1m, Extended Data 1o,r,t

Anti-mouse IgGk horseradish peroxidase (HRP) (Cat. #: SC-516102; final use concentration: 0.04 µg/mL; Clone: NA; Lot#F1620; Santa Cruz Biotechnology (Dallas, TX))
•Website https://www.scbt.com/p/m-igg-kappa-bp-hrp
•Manufacturer verified for use: western blot, immunohistochemistry
•Relevant Reference(s): Mani R, Rose S, Suresh A, Sambantham S, Anandan B, Ibrahim M, Meena B. Cellular alterations and damage to the renal tissue of marine catfish Arius arius following Cd exposure and the possible sequestrant role of Metallothionein. Mar Pollut Bull. 2021 Feb;163:111930. doi: 10.1016/j.marpolbul.2020.111930. Epub 2020 Dec 26. PMID: 33373888.

Franco-Molina MA, Santana-Krímskaya SE, Madrigal-de-León LM, Coronado-Cerda EE, Zárate-Triviño DG, Hernández-Martínez SP, García-Coronado PL, Rodríguez-Padilla C. Evaluation of the cytotoxic and immunogenic potential of temozolamide, panobinostat, and Lophophora williamsii extract against C6 glioma cells. EXCLI J. 2021 Mar 9;20:614-624. doi: 10.17179/excli2020-3181. PMID: 33883986; PMCID: PMC8056056
•Associated Figure(s): Fig 2a-c, Fig 3c, Extended Data Fig 1l, Extended Data Fig 5b

Mouse anti-rabbit HRP (Cat. #: SC-2357; final use concentration: 0.04 µg/mL; Clone# NA; Lot# G0720; Santa Cruz Biotechnology (Dallas, TX))
•Website https://www.scbt.com/p/mouse-anti-rabbit-igg-hrp
•Manufacturer verified for use: western blot, immunohistochemistry
•Relevant Reference(s): Mani R, Rose S, Suresh A, Sambantham S, Anandan B, Ibrahim M, Meena B. Cellular alterations and damage to the renal tissue of marine catfish Arius arius following Cd exposure and the possible sequestrant role of Metallothionein. Mar Pollut Bull. 2021 Feb;163:111930. doi: 10.1016/j.marpolbul.2020.111930. Epub 2020 Dec 26. PMID: 33373888
Guo L, Bai Y, Ni T, Li Y, Cao R, Ji S, Li S. MicroRNA-153-3p suppresses retinoblastoma cell growth and invasion via targeting the IGF1R/Raf/MEK and IGF1R/PI3K/AKT signaling pathways. Int J Oncol. 2021 Jul;59(1):47. doi: 10.3892/ijo.2021.5227. Epub 2021 May 26. PMID: 34036380; PMCID: PMC8143749.
•Associated Figure(s): Fig 2a-c, Fig 3c, Extended Data Fig 1l, Extended Data Fig 5b

Goat anti-rabbit AlexaFluor 488 (Cat. #: A11008; final use concentration: 0.04 µg/mL; Clone # NA; Lot #1275894; ThermoFisher Scientific (Waltham, MA))
•Website https://www.thermofisher.com/antibody/product/Goat-anti-Rabbit-IgG-H-L-Highly-Cross-Adsorbed-Secondary-Antibody-Polyclonal/A32731
•Manufacturer verified for use: western blot, immunocytochemistry
•Relevant Reference(s): Saju JM, Hossain MS, Liew WC, Pradhan A, Thevasagayam NM, Tan LSE, Anand A, Olsson PE, Orbán L. Heat Shock Factor 5 Is Essential for Spermatogenesis in Zebrafish. Cell Rep. 2018 Dec 18;25(12):3252-3261.e4. doi: 10.1016/j.celrep.2018.11.090. PMID: 30566854
Fetoni AR, Zorzi V, Paciello F, Ziraldo G, Peres C, Raspa M, Scavizzi F, Salvatore AM, Crispino G, Tognola G, Gentile G, Spampinato AG, Cuccaro D, Guarnaccia M, Morello G, Van Camp G, Fransen E, Brumat M, Girotto G, Paludetti G, Gasparini P, Cavallaro S, Mammano F. Cx26 partial loss causes accelerated presbycusis by redox imbalance and dysregulation of Nfr2 pathway. Redox Biol. 2018 Oct;19:301-317. doi: 10.1016/j.redox.2018.08.002. Epub 2018 Aug 7. PMID: 30199819; PMCID: PMC6129666.
•Associated Figure(s): Fig 3e, Extended Data 5b

Donkey anti-mouse AlexaFluor 594 (Cat. #: A21203; final use concentration: 0.05 µg/mL; Clone #NA; Lot #1918277; ThermoFisher Scientific (Waltham, MA))
•Website https://www.thermofisher.com/antibody/product/Donkey-anti-Mouse-IgG-H-L-Highly-Cross-Adsorbed-Secondary-Antibody-Polyclonal/A32744
•Manufacturer verified for use: immunocytochemistry
•Relevant Reference(s): Komabayashi-Suzuki M, Yamanishi E, Watanabe C, Okamura M, Tabata H, Iwai R, Ajioka I, Matsushita J, Kidoya H, Takakura N, Okamoto T, Kinoshita K, Ichihashi M, Nagata KI, Ema M, Mizutani KI. Spatiotemporally Dependent Vascularization Is Differently Utilized among Neural Progenitor Subtypes during Neocortical Development. Cell Rep. 2019 Oct 29;29(5):1113-1129.e5. doi: 10.1016/j.celrep.2019.09.048. PMID: 31665628
•Associated Figure(s): Fig 1i-k

AlexaFluor 594 donkey anti-rabbit (Cat. #: A21207; final use concentration: 0.05 µg/mL; Clone #NA; Lot #1938375; ThermoFisher Scientific (Waltham, MA))
•Website https://www.thermofisher.com/antibody/product/Donkey-anti-Rabbit-IgG-H-L-Highly-Cross-Adsorbed-Secondary-Antibody-Polyclonal/A-21207
•Manufacturer verified for use: immunocytochemistry, immunohistochemistry, flow cytometry, immunocytochemistry
•Relevant Reference(s): Baskfield A, Li R, Beers J, Zou J, Liu C, Zheng W. An induced pluripotent stem cell line (TRNDi009-C) from a Niemann-Pick disease type A patient carrying a heterozygous p.L302P (c.905 T>C) mutation in the SMPD1 gene. Stem Cell Res. 2019 Jul;38:101461. doi: 10.1016/j.scr.2019.101461. Epub 2019 May 15. PMID: 31132580; PMCID: PMC6686851
Hong J, Xu M, Li R, Cheng YS, Kouznetsova J, Beers J, Liu C, Zou J, Zheng W. Generation of an induced pluripotent stem cell line (TRNDi008-A) from a Hunter syndrome patient carrying a hemizygous 208insC mutation in the IDS gene. Stem Cell Res. 2019 May;37:101451. doi: 10.1016/j.scr.2019.101451. Epub 2019 Apr 25. PMID: 31071499; PMCID: PMC6642610
•Associated Figure(s): Fig 3d

Rabbit anti-Mart1 (Cat. #: SAB4500949; final use concentration: 0.2 µg/mL; Clone #NA; Lot #310257; Millipore Sigma (St. Louis, MO)
•Website https://www.sigmaaldrich.com/US/en/product/sigma/sab4500949?context=product
•Manufacturer verified for use: ELISA, immunohistochemistry, western blot
•Relevant Reference(s): Valmori D, Fonteneau JF, Lizana CM, Gervois N, Liénard D, Rimoldi D, Jongeneel V, Jotereau F, Cerottini JC, Romero P. Enhanced generation of specific tumor-reactive CTL in vitro by selected Melan-A/MART-1 immunodominant peptide analogues. J Immunol. 1998 Feb 15;160(4):1750-8. PMID: 9469433.
Chiu CG, Nakamura Y, Chong KK, Huang SK, Kawas NP, Triche T, Elashoff D, Kiyohara E, Irie RF, Morton DL, Hoon DS. Genome-wide characterization of circulating tumor cells identifies novel prognostic genomic alterations in systemic melanoma metastasis. Clin Chem. 2014 Jun;60(6):873-85. doi: 10.1373/clinchem.2013.213611. Epub 2014 Apr 9. PMID: 24718909
•Associated Figure(s): Fig 1i-k, Fig 5c-f, Extended Data Fig 1a,d,l, Extended Data Fig 4 b-h

Rabbit anti-S100 (Cat. #: Z0311; final use concentration: 0.2 µg/mL; Clone #NA; Lot #NA; Agilent Technologies (Santa Clara, CA))
•Website https://www.agilent.com/en/product/immunohistochemistry/antibodies-controls/primary-antibodies/s100-(dako-omnis)-76198
•Manufacturer verified for use: immunohistochemistry
•Relevant Reference(s): NA
•Associated Figure(s): Fig 1i-k, Fig 5c-f, Extended Data Fig 1a,d,l, Extended Data Fig 7 b-h

Rat APC anti-mouse CD3 (Cat. #: 100236; final use concentration: 0.5 µg/mL; Clone #17A2; Lot #B274725; Biolegend, inc. (San Diego, CA))

•Website https://www.biolegend.com/en-us/products/apc-anti-mouse-cd3-antibody-8055
•Manufacturer verified for use: flow cytometry
•Relevant Reference(s): Liu Y, et al. 2017. Oncogene. 10.1038/onc.2017.209
Agelidis A, et al. 2017. Cell Rep. 10.1016/j.celrep.2017.06.041
•Associated Figure(s): Fig 1c,d,g,h,o, Fig 2h, Extended Data Fig 1b,c,g,h,i,j,m,n,q,w, Extended Data Fig 3e

Rat Pacific Blue anti-mouse CD4 (Cat. #: 100428; final use concentration: 0.5 μg/mL; Clone #GK1.5; Lot #B265546; Biolegend, Inc. (San Diego, CA))
•Websitehttps://www.biolegend.com/en-us/products/pacific-blue-anti-mouse-cd4-antibody-3316?GroupID=BLG4745
•Manufacturer verified for use: flow cytometry
•Relevant Reference(s): Borkner L, et al. 2017. J Immunol. 10.4049/jimmunol.1602115
Vacca M, et al. 2017. Front Immunol. . 10.3389/fimmu.2017.01462
•Associated Figure(s): Fig 1d,h,o, Fig 2h, Extended Data Fig 1j,n,q,w, Extended Data Fig 3e

Rat BV785 anti-mouse CD8 (Cat. #: 100750; final use concentration: 0.5 μg/mL; Clone #53-6.7; Lot #B266841; Biolegend (San Diego, CA))
•Website https://www.biolegend.com/en-us/products/brilliant-violet-785-anti-mouse-cd8a-antibody-7957
•Manufacturer verified for use: flow cytometry
•Relevant Reference(s): Shih FF, et al. 2006. J. Immunol. 176:3438. Bouwer HGA, et al. 2006.
P. Natl. Acad. Sci. USA 103:5102.
•Associated Figure(s): Fig 1d,h,o, Fig 2h, Extended Data Fig 1j,w, Extended Data Fig 3e

Rat FITC anti-mouse F4/80 (Cat. #: 123108; final use concentration: 0.5 μg/mL; Clone #BM8; Lot #B257637; Biolegend, Inc (San Diego, CA))
•Website https://www.biolegend.com/en-us/products/fitc-anti-mouse-f4-80-antibody-4067
•Manufacturer verified for use: flow cytometry
•Relevant Reference(s): Kobayashi M, et al.2008. J. Leukoc. Biol. 83:1354
Poeckel D, et al. 2009. J. Biol Chem. 284:21077
•Associated Figure(s): Fig 1c,g, Extended Data Fig 1b,c,g,h,i,m

Rat APC anti-mouse GR-1 (Cat. #: 108412; final use concentration: 0.5 μg/mL; Clone #RB6-8C5; Lot #B288472; Biolegend, Inc. (San Diego, CA))
•Website https://www.biolegend.com/en-us/products/apc-anti-mouse-ly-6g-ly-6c-gr-1-antibody-456
•Manufacturer verified for use: flow cytometry
•Relevant Reference(s): Andoniou CE, et al. 2005. Nature Immunology 6:1011
Dzhagalov I, et al. 2007. Blood 109:1620
•Associated Figure(s): Fig 1c, Extended Data Fig 1b,c,g,i

Armenian Hamster PeCy7 anti-mouse CD11c (Cat. #: 117318; final use concentration: 0.5 μg/mL; Clone #N418; Lot #B269973; Biolegend, Inc. (San Diego, CA))
•Website https://www.biolegend.com/en-us/products/pe-cyanine7-anti-mouse-cd11c-antibody-3086
•Manufacturer verified for use: flow cytometry
•Relevant Reference(s): Cervantes-Barragan L, et al. 2007. Blood 109:1131.
Turnquist HR, et al. 2007. J. Immunol. 178:7018. (FC)
•Associated Figure(s): Fig 1c,g,o, Fig 2h, Extended Data Fig 1b,c,g,I,m,w

Mouse PE anti-mouse NK1.1 (Cat. #: 156504; final use concentration: 0.5 μg/mL; Clone #PK136; Lot #B277026; Biolegend, Inc. (San Diego, CA))
•Website https://www.biolegend.com/en-us/products/pe-anti-mouse-nk-1-1-antibody-16926
•Manufacturer verified for use: flow cytometry
•Relevant Reference(s): Lanier LL. 1997. Immunity 6:371.
Yokoyama WM, Seaman WE. 1993. Annu. Rev. Immunol. 11:613.
•Associated Figure(s): Extended Data Fig 1g,h,i,j,w

Rat PE anti-mouse CD49b (Cat. #: 108908; final use concentration: 0.5 μg/mL; Clone #DX5; Lot #4289830; Biolegend, Inc. (San Diego, CA))
•Website https://www.biolegend.com/en-us/products/pe-anti-mouse-cd49b-pan-nk-cells-antibody-234
•Manufacturer verified for use: flow cytometry
•Relevant Reference(s): Del Barco Barrantes I, et al. 2018. Stem Cell Reports. 10:257.
Seong J, et al. 2018. Development. 145:14
•Associated Figure(s): Fig 1c,g,o, Extended Data Fig 1b,c,m,w

Rat PerCP-Cy5.5 anti-mouse CD11b (Cat. #: 101228; final use concentration: 0.5 μg/mL; Clone #M1/70; Lot #B256661; Biolegend, Inc. (San Diego, CA))
•Website https://www.biolegend.com/en-us/products/percp-cyanine5-5-anti-mouse-human-cd11b-antibody-4257
•Manufacturer verified for use: flow cytometry
•Relevant Reference(s): Milasan A, et al. 2017. J Am Heart Assoc. 10.1161/JAHA.117.006892. Bennett FC, et al. 2018. Neuron. 98:1170
•Associated Figure(s): Fig 1c,g,o, Fig 2h, Extended Data Fig 1b,c,g,I,m,w

Rabbit anti-human PD-L1 (Cat. #: NBP1-76769; final use concentration: 0.5 μg/mL; clone #NBP1-76769; Lot #8293-1701; Novus Biologicals, Centennial, CO)
•Website https://www.novusbio.com/products/pd-l1-antibody_nbp1-76769
•Manufacturer verified for use: western blot, ELISA, flow cytometry, immunocytochemistry/immunofluorescence, immunohistochemistry (FFPE/frozen)
•Relevant Reference(s): Holokai L, Chakrabarti J, Lundy J et al. Murine- and Human-Derived Autologous Organoid/Immune Cell Co-

Cultures as Pre-Clinical Models of Pancreatic Ductal Adenocarcinoma Cancers Dec 17 2020. Yang A, Li MY, Zhang ZH et al. Erianin regulates programmed cell death ligand 1 expression and enhances cytotoxic T lymphocyte activity J Ethnopharmacol Nov 18 2020 [PMID: 33220359] (IHC, ICC/IF)
•Associated Figure(s): Extended Data Fig. 7i

Rat PE anti-mouse PD-L1 (Cat. #: 124308; final use concentration: 0.5 µg/mL; clone #10F.9G2; Lot #B278301; Biolegend, (San Diego, CA))
•Website https://www.biolegend.com/en-us/products/pe-anti-mouse-cd274-b7-h1-pd-l1-antibody-4497
•Manufacturer verified for use: flow cytometry
•Relevant Reference(s): Maier H, et al. 2007. J. Immunol. 178:2714. Meng Q, et al. 2006. Invest. Ophthalmol. Vis. Sci. 47:4444.
•Associated Figure(s): Extended Data Fig. 7i

Phalloidin Alexafluor 488 (Cat. #: A12379; final use concentration: 0.2µg/mL; clone #NA; Lot #1737901; ThermoFisher Scientific (Waltham, MA))
•Website https://www.thermofisher.com/order/catalog/product/A12379?ef_id=CjwKCAjwieuGBhAsEiwA1Ly_nbHglCspHMlFqajO6O2qiH_nv_inLTLvWwYXUfQUMh1OCprNzU7-VBoCUI4QAvD_BwE:G:s&s_kwcid=AL!3652!3!447292198730!b!!g!!&cid=bid_pca_iva_r01_co_cp1359_pjt0000_bid00000_0se_gaw_dy_pur_con&gclid=CjwKCAjwieuGBhAsEiwA1Ly_nbHglCspHMlFqajO6O2qiH_nv_inLTLvWwYXUfQUMh1OCprNzU7-VBoCUI4QAvD_BwE#/A12379?ef_id=CjwKCAjwieuGBhAsEiwA1Ly_nbHglCspHMlFqajO6O2qiH_nv_inLTLvWwYXUfQUMh1OCprNzU7-VBoCUI4QAvD_BwE:G:s&s_kwcid=AL!3652!3!447292198730!b!!g!!&cid=bid_pca_iva_r01_co_cp1359_pjt0000_bid00000_0se_gaw_dy_pur_con&gclid=CjwKCAjwieuGBhAsEiwA1Ly_nbHglCspHMlFqajO6O2qiH_nv_inLTLvWwYXUfQUMh1OCprNzU7-VBoCUI4QAvD_BwE
•Manufacturer verified for use: immunofluorescent immunocytochemistry
•Relevant Reference(s): Baddeley D, Crossman D, Rossberger S, Cheyne JE, Montgomery JM, Jayasinghe ID, Cremer C, Cannell MB, Soeller C,
PLoS One (2011) 6:e20645-e20645
•Associated Figure(s): Extended Data Fig. 7c

Mouse anti-FLAG (Cat. #: F1804; final use concentration: 0.2 µg/mL; clone #M2; Lot #SLBQ7119V; Millipore Sigma (St. Louis, MO))
•Website https://www.sigmaaldrich.com/US/en/substance/monoclonalantiflagm2antibodyproducedinmouse1234598765?context=product
•Manufacturer verified for use: western blot, immunoprecipitation, immunohistochemistry, immunofluorescence, immunocytochemistry.
•Relevant Reference(s):
Monika Srivastava et al. Nature communications, 6, 6253-6253 (2015-02-24)
•Associated Figure(s): Extended Data Fig. 1i, extended data 6c

Rabbit polyclonal anti-HLA-A (Cat. #: 15240-1-AP; final use concentration: 0.2 µg/mL; clone # NA; Lot #00006298; Proteintech (Rosemont, IL))
•Website https://www.ptglab.com/products/HLA-A-Antibody-15240-1-AP.htm
•Manufacturer verified for use: flow cytometry, immunofluorescence, immunohistochemistry, immunoprecipitation, western blot, ELISA
•Relevant Reference(s): Hao L et al. Front Oncol. 2020 May 6;10:615. doi: 10.3389/fonc.2020.00615
•Associated Figure(s): Fig. 2a-c

Normal mouse IgG (Cat. #: SC-2025; final use concentration: NA; clone # NA; Lot #B0619; Santa Cruz Biotechnology (Dallas, TX))
•Website https://www.scbt.com/p/normal-mouse-igg
•Manufacturer verified for use: negative control for western blot, immunoprecipitation, and immunohistochemistry
•Relevant Reference(s): Bejjani et al. Nucleic Acids Res. 2021 Mar 18;49(5):2488-2508. doi: 10.1093/nar/gkab053.
•Associated Figure(s): Fig 2c

Rabbit anti-KDEL (Cat. #: PA1-013; final use concentration: 0.1 µg/mL; clone # JB42-04; Lot # VG3025834; ThermoFisher Scientific (Waltham, MA))
•Website https://www.thermofisher.com/antibody/product/KDEL-Antibody-clone-JB42-04-Recombinant-Monoclonal/MA5-34715
•Manufacturer verified for use: western blot, immunohistochemistry, flow cytometry, immunocytochemistry (immunofluorescent)
•Relevant Reference(s): NA
•Associated Figure(s): Fig. 3d

Mouse anti-PD1 (Cat. #: BE-0146; Dosage 20 mg/kg; clone# RMP1-14; Lot# 780120S1C; Bioxcell (West Lebanon, NH))
•Website https://bxcell.com/product/invivomab-anti-m-pd-1/
•Manufacturer verified for use: immunocytochemistry, western blot, in vivo blockade of PD1:PD-L1 immune checkpoint
•Relevant Reference(s): Grasselly et al. Front Immunol. 2018 Oct 9;9:2100. doi: 10.3389/fimmu.2018.02100
•Associated Figure(s): Fig 4a & b

Donkey anti-goat plus PLA secondary antibody (Cat. #: DUO-92003; final use concentration: NA; clone# NA; Lot# SLBZ8370; Millipore Sigma (St. Louis, MO))
•Website https://www.sigmaaldrich.com/US/en/product/sigma/duo92003?context=product
•Manufacturer verified for use: proximity ligation assay
•Relevant Reference(s): A Frappaolo et al. Methods in cell biology, 137, 85-103 (2017-01-10), Annabel Christ et al. Developmental cell, 22(2), 268-278 (2012-02-22)
•Associated Figure(s): Fig. 5b-5f, Extended Data Fig. 7ch

Donkey anti-mouse minus PLA secondary antibody (Cat. #: DUO-92004; final use concentration: NA clone# NA; Lot# SLCC8780; Millipore Sigma (St. Louis, MO))
•Website https://www.sigmaaldrich.com/US/en/product/sigma/duo92004?context=product

•Manufacturer verified for use: proximity ligation assay
•Relevant Reference(s): A Frappaolo et al. Methods in cell biology, 137, 85-103 (2017-01-10), Annabel Christ et al. Developmental cell, 22(2), 268-278 (2012-02-22)
•Associated Figure(s): Fig. 5b-5f, Extended Data Fig. 7cf

Rat anti-mouse CD8 (Cat. #: 550-281; final use concentration: 0.2 µg/mL; Clone# 53-6.7; Lot #5338976; BD Pharmingen (San Jose, CA))
•Website https://www.bdbiosciences.com/en-us/products/reagents/flow-cytometry-reagents/research-reagents/single-color-antibodies-ruo/purified-rat-anti-mouse-cd8a.550281
•Manufacturer verified for use: flow cytometry, immunohistochemistry
•Relevant Reference(s): Alexander-Miller MA, Leggatt GR, Sarin A, Berzofsky JA. Role of antigen, CD8, and cytotoxic T lymphocyte (CTL) avidity in high dose antigen induction of apoptosis of effector CTL. J Exp Med. 1996; 184(2):485-492.
•Associated Figure(s): Extended Data Fig. 1o,r

AlexaFluor 594 goat anti-rat secondary antibody (Cat. #: A11007; final use concentration: 0.05 µg/mL; Clone# NA; Lot# 1697164; ThermoFisher Scientific (Waltham, MA))
•Website https://www.thermofisher.com/antibody/product/Goat-anti-Rat-IgG-H-L-Cross-Adsorbed-Secondary-Antibody-Polyclonal/A-11007
•Manufacturer verified for use: western blot, immunohistochemistry, immunocytochemistry, flow cytometry
•Relevant Reference(s): Bancells et al., Nat Microbiol. 2019 Jan;4(1):144-154. doi: 10.1038/s41564-018-0291-7.
•Associated Figure(s): Extended Data Fig. 1o,r

Mouse anti-CD3 (Cat. #: SC-59013; final use concentration: 0.2 µg/mL; Clone# PS1; Lot # G2419; Santa Cruz Biotechnology (Dallas, TX)
•Website https://www.scbt.com/p/cd3-antibody-ps1
•Manufacturer verified for use: western blot, immunoprecipitation, immunofluorescesnce, immunohistochemistry
•Relevant Reference(s): Ino et al., Oncotarget. 2019 Feb 1;10(10):1149-1159. doi: 10.18632/oncotarget.26646.
•Associated Figure(s): Fig. 1i-1k

Mouse PE anti-pan-MHC-I (HLA-A,B,C) (Cat. #: 555553; Clone #: G46-2.6; Lot #: multiple; BD Pharmingen (San Jose, CA)
•Website https://www.bdbiosciences.com/en-us/products/reagents/flow-cytometry-reagents/research-reagents/single-color-antibodies-ruo/pe-mouse-anti-human-hla-abc.555553
•Manufacturer verified for use: flow cytometry
•Relevant Reference(s): Crisa L, Cirulli V, Ellisman MH, Ishii JK, Elices MJ, Salomon DR. Cell adhesion and migration are regulated at distinct stages of thymic T cell development: the roles of fibronectin, VLA4, and VLA5. J Exp Med. 1996; 184(1):215-228.
•Associated Figure(s): Extended Data Fig. 6h

Mouse FITC anti-pan-MHC-II (HLA-DP, DQ, DQ)(Cat. #: 555558; Clone #: Tu39; Lot #: multiple; BD Pharmingen (San Jose, CA)
•Website https://www.bdbiosciences.com/en-us/products/reagents/flow-cytometry-reagents/research-reagents/single-color-antibodies-ruo/fitc-mouse-anti-human-hla-dr-dp-dq.555558
•Manufacturer verified for use: flow cytometry
•Relevant Reference(s): Pawelec G, Ziegler A, Wernet P. Dissection of human allostimulatory determinants with cloned T cells: stimulation inhibition by monoclonal antibodies TU22, 34, 35, 36, 37, 39, 43, and 58 against distinct human MHC class II molecules. Hum Immunol. 1985; 12(3):165-176.
•Associated Figure(s): Extended Data Fig. 6h

Mouse PerPCy5.5 anti-CD45 (Cat. #: 45-9459-42; Clone #: 2D1; Lot #: multiple; Invitrogen (Waltham, MA))
•Website https://www.thermofisher.com/antibody/product/CD45-Antibody-clone-2D1-Monoclonal/45-9459-42
•Manufacturer verified for use: flow cytometry
•Relevant Reference(s): Watanabe et al., JCI Insight. 2018 Apr 5;3(7):e99573. doi: 10.1172/jci.insight.99573.
•Associated Figure(s): Extended Data Fig. 6h

Mouse APC anti-CD90 (Cat. #: 328114; Clone #: 5E10; Lot #: multiple; Biolegend (San Diego, CA))
•Website https://www.biolegend.com/en-us/products/apc-anti-human-cd90-thy1-antibody-4116
•Manufacturer verified for use: flow cytometry
•Relevant Reference(s): Craig W, et al. 1993. J. Exp. Med. 177:1331.
•Associated Figure(s): Extended Data Fig. 6h

Mouse BV421 anti EpCAM (Cat. #: 324220; Clone #: 9C4; Lot #: multiple; Biolegend (San Diego, CA)).
•Website https://www.biolegend.com/en-us/products/brilliant-violet-421-anti-human-cd326-epcam-antibody-7549
•Manufacturer verified for use: flow cytometry
•Relevant Reference(s): Lammers R, et al. 2002. Exp. Hematol. 30:537.
•Associated Figure(s): Extended Data Fig. 6h

Fixable Live/Dead stain (Biolegend (San Jose, CA)
•Website https://www.biolegend.com/en-us/products/zombie-nir-fixable-viability-kit-8657?GroupID=BLG2181
•Manufacturer verified for use: flow cytometry
•Relevant Reference(s): McMaster SR, et al. 2015. PLoS One. 10:115725.
•Associated Figure(s): Fig. 1b-d, 1f-h, 1o, 2g,h, Extended Data Fig. 1b,c,f,g,h,I,j,m,n,p,q,v,w, 3d,e, 4a

PKH26 Millipore Sigma (Cat. #: 555553; St. Louis, MO))
•Websitehttps://www.sigmaaldrich.com/US/en/product/sigma/pkh26gl?gclid=CjwKCAjwieuGBhAsEiwA1Ly_nQjfjFZr31KPneW6vzdP_OXJyufdHtdDgQD5n88STPgkzO-GumvM8hoCESoQAvD_BwE
•Manufacturer verified for use: in vitro cell labeling, flow cytometry
•Relevant Reference(s): Ofra Ben Menachem-Zidon et al. PloS one, 14(6), e0218081-e0218081 (2019-06-14)
•Associated Figure(s): Fig. 3e, Extended Fig. 5b, 7i

Biotinylated AAL (Cat. #: B-1395; final use concentration: 0.4 μg/mL; Lot #ZH0504; Vector Labs, Burlingame, CA)
•Website https://vectorlabs.com/biotinylated-aleuria-aurantia-lectin-aal.html
•Manufacturer verified for use: immunohistochemistry/immunocytochemistry, immunofluorescence, blotting, elispot, ELISA
•Relevant Reference(s): Arriagada C, Cavieres VA, Luchsinger C, González AE, Muñoz VC, Cancino J, Burgos PV, Mardones GA. GOLPH3 Regulates EGFR in T98G Glioblastoma Cells by Modulating Its Glycosylation and Ubiquitylation. Int J Mol Sci. 2020 Nov 23;21(22):8880. doi: 10.3390/ijms21228880. PMID: 33238647; PMCID: PMC7700535.
Taubenschmid J, Stadlmann J, Jost M, Klokk TI, Rillahan CD, Leibbrandt A, Mechtler K, Paulson JC, Jude J, Zuber J, Sandvig K, Elling U, Marquardt T, Thiel C, Koerner C, Penninger JM. A vital sugar code for ricin toxicity. Cell Res. 2017 Nov;27(11):1351-1364. doi: 10.1038/cr.2017.116. Epub 2017 Sep 19. PMID: 28925387; PMCID: PMC5674155
•Associated Figure(s): Fig 2c, Fig 3e, Fig 5b-e, Extended Data 5b, Extended Data Fig 7c-f

Fluorescein-conjugated AAL (Cat. #: FL-1391; final use concentration: 0.4 μg/mL; Lot# ZF0326; Vector Laboratories, Burlingame, CA)
•Website https://vectorlabs.com/fluorescein-labeled-aleuria-aurantia-lectin-aal.html
•Manufacturer verified for use: immunofluorescence
•Relevant Reference(s): Bae K, Zheng W, Ma Y, Huang Z. Real-Time Monitoring of Pharmacokinetics of Antibiotics in Biofilms with Raman-Tagged Hyperspectral Stimulated Raman Scattering Microscopy. Theranostics. 2019 Feb 14;9(5):1348-1357. doi: 10.7150/thno.32043. PMID: 30867835; PMCID: PMC6401501.
Palaiologou E, Etter O, Goggin P, Chatelet DS, Johnston DA, Lofthouse EM, Doherty R, Pearson-Farr J, Sengers BG, Torrens C, Cleal JK, Page AM, Lewis RM. Human placental villi contain stromal macrovesicles associated with networks of stellate cells. J Anat. 2020 Jan;236(1):132-141. doi: 10.1111/joa.13082. Epub 2019 Sep 11. PMID: 31512233; PMCID: PMC6904625
•Associated Figure(s): Fig 1i-k, Fig 3e, Fig 5b-e, Extended Data 1a,d,l, Extended Data 5b, Extended data 7c-f

Agarose UEA1 and AAL (Cat. #: AL-1393 and AL-1063-2, respectively; Lot# ZG0918 for AAL; Vector Laboratories, (Burlingame, CA))
•Website https://vectorlabs.com/agarose-bound-aleuria-aurantia-lectin-aal.html https://vectorlabs.com/agarose-bound-ulex-europaeus-agglutinin-i-uea-i.html
•Manufacturer verified for use: affinity chromatography
•Relevant Reference(s): Amorim Filho L, Szwarcwald CL, Mateos SOG, Leon ACMP, Medronho RA, Veloso VG, Lopes JIF, Porto LCMS, Chieppe A, Werneck GL; Grupo Hemorio de Pesquisa em Covid-19. Seroprevalence of anti-SARS-CoV-2 among blood donors in Rio de Janeiro, Brazil. Rev Saude Publica. 2020;54:69. doi: 10.11606/s1518-8787.2020054002643. Epub 2020 Jul 6. PMID: 32638883; PMCID: PMC7334006.
Liu Y, He J, Li C, Benitez R, Fu S, Marrero J, Lubman DM. Identification and confirmation of biomarkers using an integrated platform for quantitative analysis of glycoproteins and their glycosylations. J Proteome Res. 2010 Feb 5;9(2):798-805. doi: 10.1021/pr900715p. PMID: 19961239; PMCID: PMC2838716.
•Associated Figure(s): Fig 2b, Fig 3c, Extended Data 6e

Alexa Fluor 647 anti-human/mouse Granzyme B Antibody (Cat. #: 515406; Lot # B266742; Concentration: 0.5 μg/mL; Biolegend (San Diego, CA))
Website: https://www.biolegend.com/en-us/products/alexa-fluor-647-anti-human-mouse-granzyme-b-antibody-6067?GroupID=BLG15670
Manufacturer’s verified use: Flow Cytometry
Relevant References: Griffin BD, Muthumani K, Warner BM, Majer A, Hagan M, Audet J, Stein DR, Ranadheera C, Racine T, De La Vega MA, Piret J, Kucas S, Tran KN, Frost KL, De Graff C, Soule G, Scharikow L, Scott J, McTavish G, Smid V, Park YK, Maslow JN, Sardesai NY, Kim JJ, Yao XJ, Bello A, Lindsay R, Boivin G, Booth SA, Kobasa D, Embury-Hyatt C, Safronetz D, Weiner DB, Kobinger GP. DNA vaccination protects mice against Zika virus-induced damage to the testes. Nat Commun. 2017 Jun 7;8:15743. doi: 10.1038/ncomms15743. PMID: 28589934; PMCID: PMC5467228.
Gordon-Alonso M, Hirsch T, Wildmann C, van der Bruggen P. Galectin-3 captures interferon-gamma in the tumor matrix reducing chemokine gradient production and T-cell tumor infiltration. Nat Commun. 2017 Oct 6;8(1):793. doi: 10.1038/s41467-017-00925-6. PMID: 28986561; PMCID: PMC5630615.
Associated Figures: Extended Data Fig 3d & e

BUV805 Rat Anti-Mouse CD8a (Cat. # 612898; Lot # 1194016; Concentration: 0.5 μg/mL; BD Biosciences (Franklin Lakes, NJ))
Website: https://www.bdbiosciences.com/en-us/products/reagents/flow-cytometry-reagents/research-reagents/single-color-antibodies-ruo/buv805-rat-anti-mouse-cd8a.612898
Manufacturer’s verified use: Flow Cytometry
Relevant References: Fujiura Y, Kawaguchi M, Kondo Y, et al. Development of CD8 alpha alpha+ intestinal intraepithelial T cells in beta 2-microglobulin- and/or TAP1-deficient mice. J Immunol. 1996; 156(8):2710-2715. (Clone-specific: Flow cytometry)
Ledbetter JA, Rouse RV, Micklem HS, Herzenberg LA. T cell subsets defined by expression of Lyt-1,2,3 and Thy-1 antigens. Two-parameter immunofluorescence and cytotoxicity analysis with monoclonal antibodies modifies current views. J Exp Med. 1980; 152(2):280-295.
Associated Figures: Extended Data Fig 3c-e, Fig. 4b

BV421 Rat Anti-Mouse I-A/I-E (Cat.# 562564 Lot # 1074223; Concentration: 0.5 μg/mL; BD Biosciences (Franklin Lakes, NJ))
Website: https://www.bdbiosciences.com/en-us/products/reagents/flow-cytometry-reagents/research-reagents/single-color-antibodies-ruo/bv421-rat-anti-mouse-i-a-i-e.562564
Manufacturer’s verified use: Flow Cytometry, Immunofluorescence
Relevant References: Ernst DN, McQuitty DN, Weigle WO, Hobbs MV. Expression of membrane activation antigens on murine B lymphocytes stimulated with lipopolysaccharide. Cell Immunol. 1988; 114(1):161-173
Viville S, Neefjes J, Lotteau V, et al. Mice lacking the MHC class II-associated invariant chain. Cell. 1993; 72(4):635-648.
Associated Figures: Extended Data Fig 3c, Fig. 4b

Live/Dead Aqua (Cat# L34965; Lot #: multiple; ThermoFisher Scientific (Waltham, MA))
Website:https://www.thermofisher.com/order/catalog/product/L34957?s_kwcid=AL!3652!3!358452930868!e!!g!!live%20dead%20aqua&ef_id=CjwKCAjw8sCRBhA6EiwA6_IF4YuAc427TmvCQ_pkCYMPlc2WPRiJJVnVdniFZLRrqcfT57IgVnt6uxoCCaQQAvD_BwE:G:s&s_kwcid=AL!3652!3!358452930868!e!!g!!live%20dead%20aqua&cid=bid_pca_frg_r01_co_cp1359_pjt0000_bid00000_0se_gaw_bt_pur_con&gclid=CjwKCAjw8sCRBhA6EiwA6_IF4YuAc427T

mvCQ_pkCYMPlc2WPRiJJVnVdniFZLRrqcfT57IgVnt6uxoCCaQQAvD_BwE
Manufacturer's verified use: Flow Cytometry
Relevant References: Zeng X, Wei YL, Huang J, Newell EW, Yu H, Kidd BA, Kuhns MS, Waters RW, Davis MM, Weaver CT, Chien YH. γδ T cells recognize a microbial encoded B cell antigen to initiate a rapid antigen-specific interleukin-17 response. Immunity. 2012 Sep 21;37(3):524-34. doi: 10.1016/j.immuni.2012.06.011. Epub 2012 Sep 6. PMID: 22960222; PMCID: PMC3495981.
Idoyaga J, Suda N, Suda K, Park CG, Steinman RM. Antibody to Langerin/CD207 localizes large numbers of CD8alpha+ dendritic cells to the marginal zone of mouse spleen. Proc Natl Acad Sci U S A. 2009 Feb 3;106(5):1524-9. doi: 10.1073/pnas.0812247106. Epub 2009 Jan 23. PMID: 19168629; PMCID: PMC2635812.
Associated Figures: Extended Data Fig 3c-e, Fig. 4b

BV605 anti-mouse CD11c (Cat. #:117334; Lot # B342521; Concentration: 0.5 μg/mL; Biolegend (San Diego, CA))
Website: https://www.biolegend.com/en-us/products/brilliant-violet-605-anti-mouse-cd11c-antibody-7865?GroupID=BLG11937
Manufacturer's verified use: Flow Cytometry
Relevant References: de Mingo Pulido Á, Gardner A, Hiebler S, Soliman H, Rugo HS, Krummel MF, Coussens LM, Ruffell B. TIM-3 Regulates CD103+Dendritic Cell Function and Response to Chemotherapy in Breast Cancer. Cancer Cell. 2018 Jan 8;33(1):60-74.e6. doi: 10.1016/j.ccell.2017.11.019. PMID: 29316433; PMCID: PMC5764109.
Medler TR, Murugan D, Horton W, Kumar S, Cotechini T, Forsyth AM, Leyshock P, Leitenberger JJ, Kulesz-Martin M, Margolin AA, Werb Z, Coussens LM. Complement C5a Fosters Squamous Carcinogenesis and Limits T Cell Response to Chemotherapy. Cancer Cell. 2018 Oct 8;34(4):561-578.e6. doi: 10.1016/j.ccell.2018.09.003. PMID: 30300579; PMCID: PMC6246036.
Associated Figures: Extended Data Fig 3c, Fig. 4b

BV711 Rat anti-mouse CD11b (Cat.# 563168; Lot # 1116158; Concentration: 0.5 μg/mL; BD Biosciences (Franklin Lakes, NJ))
Website: https://www.bdbiosciences.com/en-us/products/reagents/flow-cytometry-reagents/research-reagents/single-color-antibodies-ruo/bv711-rat-anti-cd11b.563168
Manufacturer's verified use: Flow Cytometry
Relevant References: Ault KA, Springer TA. Cross-reaction of a rat-anti-mouse phagocyte-specific monoclonal antibody (anti-Mac-1) with human monocytes and natural killer cells. J Immunol. 1981 Jan;126(1):359-64. PMID: 7451976.
Driver DJ, McHeyzer-Williams LJ, Cool M, Stetson DB, McHeyzer-Williams MG. Development and maintenance of a B220- memory B cell compartment. J Immunol. 2001 Aug 1;167(3):1393-405. doi: 10.4049/jimmunol.167.3.1393. PMID: 11466358.
Associated Figures: Extended Data Fig 3c-e, Fig 4b

BV421 Rat anti-mouse CD44 (Cat.# 563970; Lot #0337154; Concentration: 0.5 μg/mL; BD Biosciences (Franklin Lakes, NJ))
Website: https://www.bdbiosciences.com/en-us/products/reagents/flow-cytometry-reagents/research-reagents/single-color-antibodies-ruo/bv421-rat-anti-mouse-cd44.563970
Manufacturer's verified use: Flow Cytometry
Relevant References: Ernst DN, Weigle WO, Noonan DJ, McQuitty DN, Hobbs MV. The age-associated increase in IFN-gamma synthesis by mouse CD8+ T cells correlates with shifts in the frequencies of cell subsets defined by membrane CD44, CD45RB, 3G11, and MEL-14 expression. J Immunol. 1993 Jul 15;151(2):575-87. PMID: 7687616.
Hathcock KS, Hirano H, Murakami S, Hodes RJ. CD44 expression on activated B cells. Differential capacity for CD44-dependent binding to hyaluronic acid. J Immunol. 1993 Dec 15;151(12):6712-22. PMID: 7505013.
Associated Figures: Extended Data Fig 3c, Fig 4b

BV650 Rat anti-mouse CD4 (Cat.# 563747; Lot #1013120; Concentration: 0.5 μg/mL; BD Biosciences (Franklin Lakes, NJ))
Website: https://www.bdbiosciences.com/en-us/products/reagents/flow-cytometry-reagents/research-reagents/single-color-antibodies-ruo/bv650-rat-anti-mouse-cd4.563747
Manufacturer's verified use: Flow Cytometry
Relevant References: Godfrey DI, Kennedy J, Mombaerts P, Tonegawa S, Zlotnik A. Onset of TCR-beta gene rearrangement and role of TCR-beta expression during CD3-CD4-CD8- thymocyte differentiation. J Immunol. 1994 May 15;152(10):4783-92. PMID: 7513723.
Tanaka T, Tsudo M, Karasuyama H, Kitamura F, Kono T, Hatakeyama M, Taniguchi T, Miyasaka M. A novel monoclonal antibody against murine IL-2 receptor beta-chain. Characterization of receptor expression in normal lymphoid cells and EL-4 cells. J Immunol. 1991 Oct 1;147(7):2222-8. PMID: 1918958.
Associated Figures: Extended Data Fig 3c-e, Fig 4b

BV786 Rat anti-mouse CD45 (Cat.# 564225; Lot #1175935; Concentration: 0.5 μg/mL; BD Biosciences (Franklin Lakes, NJ))
Website: https://www.bdbiosciences.com/en-us/products/reagents/flow-cytometry-reagents/research-reagents/single-color-antibodies-ruo/bv786-rat-anti-mouse-cd45.564225
Manufacturer's verified use: Flow Cytometry
Relevant References: Salazar GA, Peñaloza HF, Pardo-Roa C, Schultz BM, Muñoz-Durango N, Gómez RS, Salazar FJ, Pizarro DP, Riedel CA, González PA, Alvarez-Lobos M, Kalergis AM, Bueno SM. Interleukin-10 Production by T and B Cells Is a Key Factor to Promote Systemic Salmonella enterica Serovar Typhimurium Infection in Mice. Front Immunol. 2017 Aug 2;8:889. doi: 10.3389/fimmu.2017.00889. PMID: 28824622; PMCID: PMC5539121.
Gallizioli M, Miró-Mur F, Otxoa-de-Amezaga A, Cugota R, Salas-Perdomo A, Justicia C, Brait VH, Ruiz-Jaén F, Arbaizar-Rovirosa M, Pedragosa J, Bonfill-Teixidor E, Gelderblom M, Magnus T, Cano E, Del Fresno C, Sancho D, Planas AM. Dendritic Cells and Microglia Have Non-redundant Functions in the Inflamed Brain with Protective Effects of Type 1 cDCs. Cell Rep. 2020 Oct 20;33(3):108291. doi: 10.1016/j.celrep.2020.108291. PMID: 33086061; PMCID: PMC7578563.
Associated Figures: Extended Data Fig 3c-e, Fig 4b

APC anti-mouse F4/80 (Cat. #: 123116; Lot # B298926; Concentration: 0.5 μg/mL; Biolegend (San Diego, CA))
Website: https://www.biolegend.com/en-gb/products/apc-anti-mouse-f4-80-antibody-4071?GroupID=BLG5319
Manufacturer's verified use: Flow Cytometry
Relevant References: Qi F, Bai S, Wang D, Xu L, Hu H, Zeng S, Chai R, Liu B. Macrophages produce IL-33 by activating MAPK signaling pathway during RSV infection. Mol Immunol. 2017 Jul;87:284-292. doi: 10.1016/j.molimm.2017.05.008. Epub 2017 May 19. PMID: 28531812.
Gonçalves NP, Moreira J, Martins D, Vieira P, Obici L, Merlini G, Saraiva M, Saraiva MJ. Differential expression of Cathepsin E in transthyretin amyloidosis: from neuropathology to the immune system. J Neuroinflammation. 2017 Jun 6;14(1):115. doi: 10.1186/

s12974-017-0891-9. PMID: 28583160; PMCID: PMC5460450.
Associated Figures: Extended Data Fig 3c, Fig 4b

PeCy anti-mouse CD103 (Cat. #: 121426; Lot # B283947; Concentration: 0.5 µg/mL; Biolegend (San Diego, CA))
Website: https://www.biolegend.com/en-us/products/pe-cyanine7-anti-mouse-cd103-antibody-9899?GroupID=BLG7093
Manufacturer's verified use: Flow Cytometry
Relevant References: de Mingo Pulido Á, Gardner A, Hiebler S, Soliman H, Rugo HS, Krummel MF, Coussens LM, Ruffell B. TIM-3 Regulates CD103+Dendritic Cell Function and Response to Chemotherapy in Breast Cancer. Cancer Cell. 2018 Jan 8;33(1):60-74.e6. doi: 10.1016/j.ccell.2017.11.019. PMID: 29316433; PMCID: PMC5764109.
Hernández-Santos N, Wiesner DL, Fites JS, McDermott AJ, Warner T, Wüthrich M, Klein BS. Lung Epithelial Cells Coordinate Innate Lymphocytes and Immunity against Pulmonary Fungal Infection. Cell Host Microbe. 2018 Apr 11;23(4):511-522.e5. doi: 10.1016/j.chom.2018.02.011. Epub 2018 Mar 22. Erratum in: Cell Host Microbe. 2019 Apr 10;25(4):630. PMID: 29576482; PMCID: PMC5951304.
Associated Figures: Extended Data Fig 3c, Fig 4b

BUV Rat anti-mouse CD62L (Cat.# 612833; Lot #111748; Concentration: 0.5 µg/mL; BD Biosciences, (Franklin Lakes, NJ))
Website: https://www.bdbiosciences.com/en-us/products/reagents/flow-cytometry-reagents/research-reagents/single-color-antibodies/buv737-rat-anti-mouse-cd62l.612833
Manufacturer's verified use: Flow Cytometry
Relevant References: Jung TM, Gallatin WM, Weissman IL, Dailey MO. Down-regulation of homing receptors after T cell activation. J Immunol. 1988 Dec 15;141(12):4110-7. PMID: 3058798.
Yang G, Mizuno MT, Hellström KE, Chen L. B7-negative versus B7-positive P815 tumor: differential requirements for priming of an antitumor immune response in lymph nodes. J Immunol. 1997 Jan 15;158(2):851-8. PMID: 8993003.
Associated Figures: Extended Data Fig 3c, Fig 4b

PeCy7 anti-mouse CRTAM (Cat. #: 142013; Lot # B285616; Concentration: 0.5 µg/mL; Biolegend, (San Diego, CA))
Website: https://www.biolegend.com/en-us/products/pe-cyanine7-anti-mouse-cd355-crtam-antibody-15073?GroupID=BLG9494
Manufacturer's verified use: Flow Cytometry
Relevant References: Takeuchi A, Badr Mel S, Miyauchi K, Ishihara C, Onishi R, Guo Z, Sasaki Y, Ike H, Takumi A, Tsuji NM, Murakami Y, Katakai T, Kubo M, Saito T. CRTAM determines the CD4+ cytotoxic T lymphocyte lineage. J Exp Med. 2016 Jan 11;213(1):123-38. doi: 10.1084/jem.20150519. Epub 2015 Dec 22. PMID: 26694968; PMCID: PMC4710199.
Chang JT, Ciocca ML, Kinjyo I, Palanivel VR, McClurkin CE, Dejong CS, Mooney EC, Kim JS, Steinel NC, Oliaro J, Yin CC, Florea BI, Overkleeft HS, Berg LJ, Russell SM, Koretzky GA, Jordan MS, Reiner SL. Asymmetric proteasome segregation as a mechanism for unequal partitioning of the transcription factor T-bet during T lymphocyte division. Immunity. 2011 Apr 22;34(4):492-504. doi: 10.1016/j.immuni.2011.03.017. Epub 2011 Apr 14. PMID: 21497118; PMCID: PMC3088519.
Associated Figures: Extended Data Fig 3d-e, Fig 4b

APC-R700 hamster anti-mouse CD95 (Cat.# 565130; Lot #1057239; Concentration: 0.5 µg/mL; BD Biosciences (Franklin Lakes, NJ))
Website: https://www.bdbiosciences.com/en-us/products/reagents/flow-cytometry-reagents/research-reagents/single-color-antibodies-ruo/apc-r700-hamster-anti-mouse-cd95.565130
Manufacturer's verified use: Flow Cytometry
Relevant References: Yang Y, Merćep M, Ware CF, Ashwell JD. Fas and activation-induced Fas ligand mediate apoptosis of T cell hybridomas: inhibition of Fas ligand expression by retinoic acid and glucocorticoids. J Exp Med. 1995 May 1;181(5):1673-82. doi: 10.1084/jem.181.5.1673. PMID: 7536793; PMCID: PMC2192012.
Ogasawara J, Suda T, Nagata S. Selective apoptosis of CD4+CD8+ thymocytes by the anti-Fas antibody. J Exp Med. 1995 Feb 1;181(2):485-91. doi: 10.1084/jem.181.2.485. PMID: 7530758; PMCID: PMC2191897.
Associated Figures: Extended Data Fig 3c, Fig 4b

PE-Dazzle Hamster anti-mouse FC�R1 (Cat. #: 139319; Lot # B343474; Concentration: 0.5 µg/mL; Biolegend (San Diego, CA))
Website: https://www.biolegend.com/en-us/products/pe-dazzle-594-anti-mouse-fcepsilonrialpha-antibody-14527?GroupID=BLG6716
Manufacturer's verified use: Flow Cytometry
Relevant References: Obata K, Mukai K, Tsujimura Y, Ishiwata K, Kawano Y, Minegishi Y, Watanabe N, Karasuyama H. Basophils are essential initiators of a novel type of chronic allergic inflammation. Blood. 2007 Aug 1;110(3):913-20. doi: 10.1182/blood-2007-01-068718. Epub 2007 Apr 4. PMID: 17409268.
Arinobu Y, Iwasaki H, Gurish MF, Mizuno S, Shigematsu H, Ozawa H, Tenen DG, Austen KF, Akashi K. Developmental checkpoints of the basophil/mast cell lineages in adult murine hematopoiesis. Proc Natl Acad Sci U S A. 2005 Dec 13;102(50):18105-10. doi: 10.1073/pnas.0509148102. Epub 2005 Dec 5. PMID: 16330751; PMCID: PMC1312421.
Associated Figures: Extended Data Fig 3c, Fig 4b

Rabbit Calnexin (C5C9) R1 (Cat. #: 2679T; Lot # 4; Concentration: 1/1000; Cell Signaling Technology (Danvers, MA))
Website: https://www.cellsignal.com/products/primary-antibodies/calnexin-c5c9-rabbit-mab/2679
Manufacturer's verified use: Western Blotting, Immunohistochemistry, Immunofluorescence
Relevant References: Rogers MA, Buffolo F, Schlotter F, Atkins SK, Lee LH, Halu A, Blaser MC, Tsolaki E, Higashi H, Luther K, Daaboul G, Bouten CVC, Body SC, Singh SA, Bertazzo S, Libby P, Aikawa M, Aikawa E. Annexin A1-dependent tethering promotes extracellular vesicle aggregation revealed with single-extracellular vesicle analysis. Sci Adv. 2020 Sep 16;6(38):eabb1244. doi: 10.1126/sciadv.abb1244. PMID: 32938681; PMCID: PMC7494353.
Stefanius K, Servage K, de Souza Santos M, Gray HF, Toombs JE, Chimalapati S, Kim MS, Malladi VS, Brekken R, Orth K. Human pancreatic cancer cell exosomes, but not human normal cell exosomes, act as an initiator in cell transformation. Elife. 2019 May 28;8:e40226. doi: 10.7554/eLife.40226. PMID: 31134894; PMCID: PMC6538373.
Associated Figures: Extended Data Fig 6d

Goat anti-Integrin �5 (Cat#: ab31327; Lot# GR68472-8; Concentration: 0.5 µg/mL; Abcam (Cambridge, UK)))
Website: https://www.abcam.com/integrin-beta-5-antibody-ab31327.html
Manufacturer's verified use: Western Blot
Relevant References: Kim W, Choi J, Yoon H, Lee J, Jun JH. Detrimental effects of lipopolysaccharide on the attachment and

outgrowth of various trophoblastic spheroids on human endometrial epithelial cells. Clin Exp Reprod Med. 2021 Jun;48(2):132-141. doi: 10.5653/cerm.2021.04448. Epub 2021 May 31. PMID: 34078006; PMCID: PMC8176151.
Dong H, Liu H, Zhou W, Zhang F, Li C, Chen J, Tan C, Tang B, Yu P. GLI1 activation by non-classical pathway integrin αvβ3/ERK1/2 maintains stem cell-like phenotype of multicellular aggregates in gastric cancer peritoneal metastasis. Cell Death Dis. 2019 Jul 31;10(8):574. doi: 10.1038/s41419-019-1776-x. PMID: 31366904; PMCID: PMC6668446.
Associated Figures: Extended Data Fig 2e

Phospho-PKA Substrate (RRXS*/T*) (Cat. #: 9624S; Lot # 21; Concentration: 1/1000; Cell Signaling (Danvers, MA))
Website: https://www.cellsignal.com/products/primary-antibodies/phospho-pka-substrate-rrxs-t-100g7e-rabbit-mab/9624#:~:text=Phospho%2DPKA%20substrate%2Dspecific%20antibodies,high%20throughput%20kinase%20drug%20discovery.
Manufacturer's verified use: Western Blotting, Immunorecipitation, ELISA
Relevant References: Li GB, Fu RQ, Shen HM, Zhou J, Hu XY, Liu YX, Li YN, Zhang HW, Liu X, Zhang YH, Huang C, Zhang R, Gao N. Polyphyllin I induces mitophagic and apoptotic cell death in human breast cancer cells by increasing mitochondrial PINK1 levels. Oncotarget. 2017 Feb 7;8(6):10359-10374. doi: 10.18632/oncotarget.14413. PMID: 28060722; PMCID: PMC5354664.
Christensen B, Nellemann B, Jørgensen JO, Pedersen SB, Jessen N. Erythropoietin does not activate erythropoietin receptor signaling or lipolytic pathways in human subcutaneous white adipose tissue in vivo. Lipids Health Dis. 2016 Sep 17;15(1):160. doi: 10.1186/s12944-016-0327-z. PMID: 27640183; PMCID: PMC5027120.
Associated Figures: Extended Data Fig 2b

Eukaryotic cell lines

Policy information about cell lines and Sex and Gender in Research

Cell line source(s)

NHEM (normal adult epidermal melanocytes) were obtained from Lonza.
WM793 (original source: Wistar Institute), 1205Lu (original source: Wistar Institute), A375 (original source: ATCC), WM1366 (original source: Wistar Institute), WM164 (original source: Wistar Institute), and SW1 melanoma cells were obtained from the Ronai laboratory (Sanford-Burnham Prebys Medical Discovery Institute (La Jolla, CA)).
WM983A and WM983B cells were purchased from Rockland Immunochemicals (Limerick, PA).
WM115 and WM266-4 cells were purchased from ATCC (Manassas, VA).
SM1 cells were obtained from the Smalley laboratory at Moffitt Cancer Center (original source was the Ribas Laboratory (Koya et al. 2012; PMID: 22693252).

Authentication

The identities of all cell lines (human and mouse) in the Lau Laboratory were initially verified as of December 2019 by short tandem repeat (STR)-based authentication "CellCheck" services provided through IDEXX BioResearch (idexxbioresearch.com). Repeat authentication of cell identities were performed annually on routinely used and newly acquired cell lines. The most recent round of authentication was performed as of October 2020. All rounds of authentication have confirmed the proper identities of all cell lines in the Lau laboratory (used in this study).

Mycoplasma contamination

In general, testing for mycoplasma, is performed once every 2-3 months on all cells in culture in the Lau laboratory, including those implemented in this study. We test using the Invivogen PlasmoTest Mycoplasma Detection Kit (https://www.invivogen.com/plasmotest). This assay delineates myco-positive from myco-negative cells using a colorimetric readout: positive cell media turns blue color (compared with a positive control in the kit); negative cell media does not.

Commonly misidentified lines
(See ICLAC register)

We used 1205Lu cells, which are listed as a commonly misidentified cell line Our authentication showed that they are pure 1205Lu cultures without contaminant lines.

Animals and other research organisms

Policy information about studies involving animals; ARRIVE guidelines recommended for reporting animal research, and Sex and Gender in Research

Laboratory animals

Four-to-six-week-old female C3H/HeN and male C57BL6 mice were purchased from Charles Rivers Laboratories for the indicated experiments.
Four-to-six-week-old male NSG mice from the Lau laboratory breeding colony were used for the indicated experiments.

Wild animals

This study did not involve wild animals.

Reporting on sex

Our study and its experiments and analyses were not focused on sex-associated outcomes/phenotypes/mechanisms. Thus, our animal modeling experiments included mice of both sexes. For example, SW1 mouse melanoma tumors (which originated in a female mouse) were grafted into syngeneic female C3H/HeN mice, whereas SM1 mouse melanoma tumors (which originated in a male mouse), were greated into syngeneic male C57BL6 mice.

Field-collected samples

This study did not involved field-collected samples.

Ethics oversight

All animals were housed at the Vincent A. Stabile Research building animal facility at H. Lee Moffitt Cancer Center & Research Institute, which is fully accredited by the Association for Assessment and Accreditation of Laboratory Animal Care International (AAALAC, #434), and are managed in accordance with the Guide for the Care and Use of Laboratory Animals ("The Guide"), the Animal Welfare Regulations Title 9 Code of Federal Regulations Subchapter A, "Animal Welfare", Parts 1-3 (AWR), the Public Health Service Policy on Humane Care and Use of Laboratory Animals (PHS Policy), and by the USF Institutional Animal Care and Use Committee's Principles and Procedures of Animal Care and Use (IACUC Principles). The experiments and protocols detailed in this

study received institutional approval by the Moffitt IACUC (RIS00001625). The Lau laboratory also has a Moffitt IACUC-approved, active NSG breeding colony (protocol: #IS00008112) from which we sourced NSG mice for the experiments indicated in the manuscript.

Note that full information on the approval of the study protocol must also be provided in the manuscript.

Clinical data

Policy information about clinical studies

All manuscripts should comply with the ICMJE guidelines for publication of clinical research and a completed CONSORT checklist must be included with all submissions.

Clinical trial registration	This study did not involve a clinical trial.
Study protocol	*Note where the full trial protocol can be accessed OR if not available, explain why.*
Data collection	*Describe the settings and locales of data collection, noting the time periods of recruitment and data collection.*
Outcomes	

Flow Cytometry

Plots

Confirm that:

☒ The axis labels state the marker and fluorochrome used (e.g. CD4-FITC).

☒ The axis scales are clearly visible. Include numbers along axes only for bottom left plot of group (a 'group' is an analysis of identical markers).

☒ All plots are contour plots with outliers or pseudocolor plots.

☒ A numerical value for number of cells or percentage (with statistics) is provided.

Methodology

Sample preparation	Surgically resected patient tumors or mouse tumors/spleens were minced to less than 1-mm fragments. Minced tumor sample was enzymatically digested in enzyme media comprised of RPMI with collagenase type IV (1 mg/mL), DNase type IV (30 U/mL), and hyaluronidase type V (100 µg/mL) (Sigma). Single cell suspensions were strained through 40-micron nylon mesh and counted for viability via trypan blue exclusion. Tumor homogenates were thawed and stained as indicated in the Methods section of the manuscript.
Instrument	BD FACsCalibur and BD LSRII Analyzer
Software	Cellquest v6, FACsDIVA v9, FlowJo v9
Cell population abundance	For Figs. 1 and 3 and Extended Data Fig. 1:<br>itICs: total # ; 1x10^6 cells total were stained and analyzed per condition/group as in Supplementary Figure S1. % of subpopulation were based on that total.<br>For Figs. 2 and 5:<br>Dendritic and CD4+T cell subpopulations: 2 x 10^6 cells for tumors and 2.5 x 10^5 for lymph nodes were fixed and immunofluorescently stained and analyzed as in Supplementary Figures S2 and S3.<br>For Fig. 4 and Extended Data Fig. 4:<br>For cell surface AAL/HLA-DRB1/PKH26: a minimum of 1x10^4 cells were stained and analyzed as in Supplementary Figure S4.<br>For Extended Data 5h:<br>For cell surface pan-MHC-I and pan-MHC-II: 5x10^5 single cells were stained and analyzed as in Supplementary Figure S5.
Gating strategy	Detailed gating strategies are illustrated in the submitted Supplementary Information file.<br>Generally, they are as follows:<br>•For intratumoral immune cell (itIC) and splenic profiling:<br>1 x 10^6 itICs or splenocytes were fixed and immunofluorescently stained with the indicated itIC subpopulation markers. The cells were first gated for FSC/SSC and live/dead cell stain (Zombie NIR). Then the live stained single cells were subsequently gated for indicated stains per cell type as indicated (APC anti-mouse CD3, Pacific Blue anti-mouse CD4, BV785 anti-mouse CD8, FITC anti-mouse F4/80, PeCy7 anti-mouse CD11c, PE anti-mouse NK1.1 or PE anti-mouse DX5, and PerCP-Cy5.5 anti-mouse CD11b).<br><br>•Assessment of dendritic subpopulations: 2 x 10^6 cells for tumors and 2.5 x 10^5 for lymph nodes were fixed and immunofluorescently stained and analyzed as in Supplementary Figures S2 and S3. Cells were gated for FSC/SSC, followed by live/dead stain (live/dead aqua). After gating for CD45+ cells, dendritic cells were gated for CD11c, using F4/80 to gate out macrophages/monocyte populations. For specific dendritic cell populations, MHCII+ cells were gated by CD103 and CD11b to identify cDC1s and FC[illegible]R1 was used to identify cDC2s and monocyte-derived dendritic cells (moDCs).

•Assessment of CD4+T cell subpopulations: 2 x 10^6 cells for tumors and 2.5 x 10^5 for lymph nodes were fixed and immunofluorescently stained and analyzed as in Supplementary Figures S2 and S3. Cells were gated for FSC/SSC, followed by live/dead stain (live/dead aqua). After gating for CD45+ cells, CD11b was used to exclude myeloid cells from the analysis. CD4 + and CD8+ subpopulations were gated from this, followed by CRTAM and Granzyme B as cytotoxic markers. For memory phenotypes, CD4+ and CD8+ cells were gated by CD62L and CD44, followed by CD95.

•Assessment of cell surface fucosylation, HLA-DRB1, and PD-L1:
Cells were first gated for FSC and SSC followed by gating for indicated stains. A minimum of 1x10^4 cells were analyzed within the singlet SSC-FSC gate for analysis of mean fluorescence intensity (MFI) of AAL-FITC, HLA-DRB1-AF488, or PKH26. AAL and HLA-DRB1 values were normalized to the respective PKH26 values (which reflect plasma membrane surface area) in order to discern AAL or HLA-DRB1 intensity per plasma membrane area.

•Assessment of cell surface pan-MHC-I and pan-MHC-II:
5x10^5 single cells were first gated for FSC and SSC followed by gating for live cells (based on live/dead cell staining (Zombie NIR) followed by gating for CD45- and CD90-/EPCAM-negative cells. Cells were then profiled for pan MHC-I (HLA-A/B/C) or pan MHC-II (HLA-DR/DP/DQ) (as shown in Supplementary Information).

**Note, flow cytometric plots that were analyzed in FlowJo were subsequently transformed into column charts, where signals measured by flow were normalized as indicated in the Methods and Figure Legends.

☒ Tick this box to confirm that a figure exemplifying the gating strategy is provided in the Supplementary Information.
