# Supplementary figures and images for: Fucosylation of HLA-DRB1 regulates CD4+ T cell-mediated anti-melanoma immunity and enhances immunotherapy efficacy
Source: Nat Cancer. 2023 Jan 23;4(2):222–39. doi: 10.1038/s43018-022-00506-7 (PMC9970875; doi:10.1038/s43018-022-00506-7)

### Figure 3A

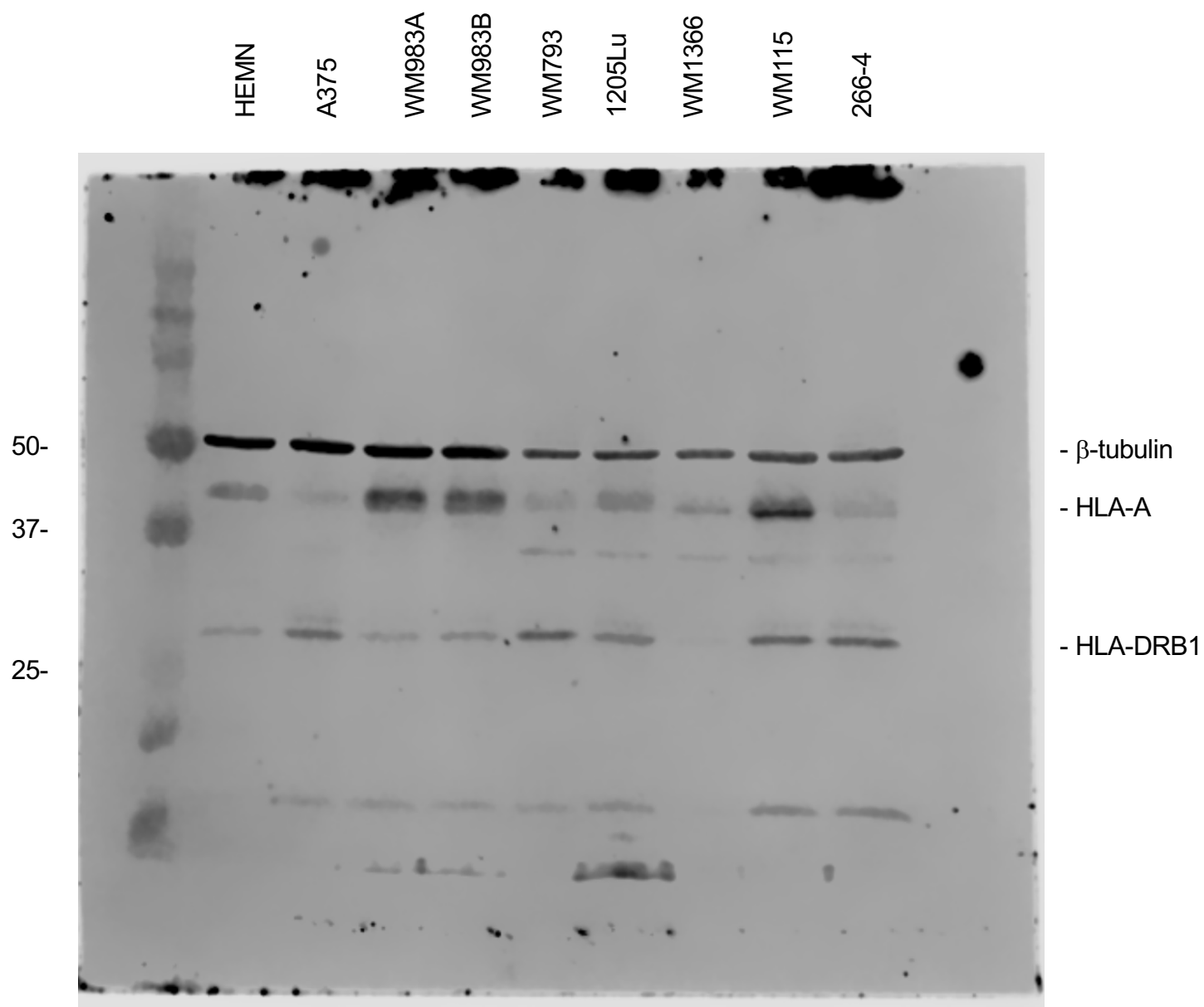

Figure 3B, left

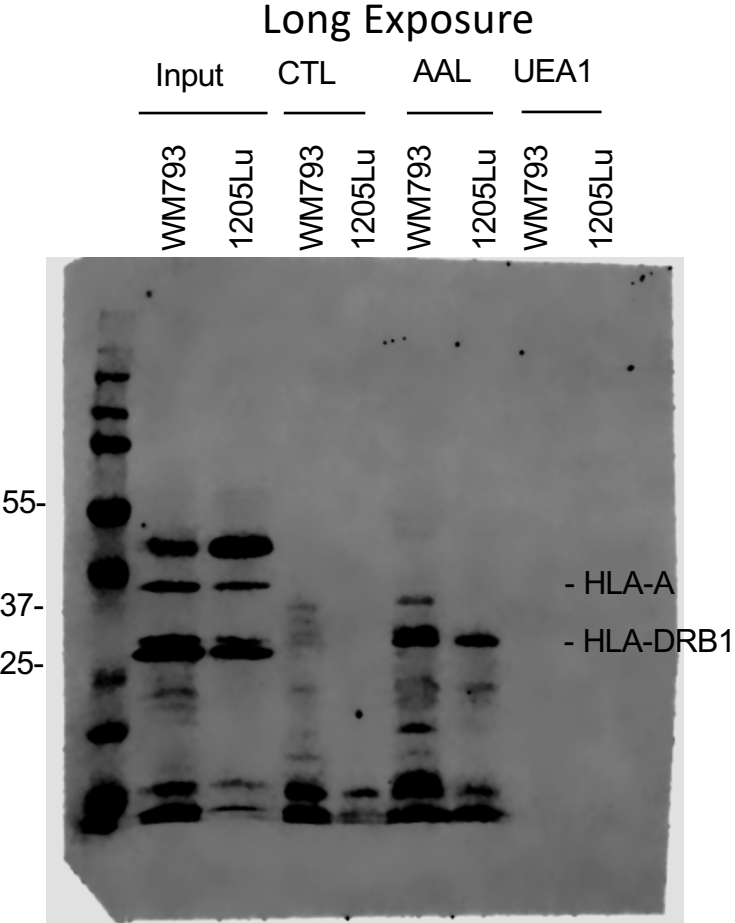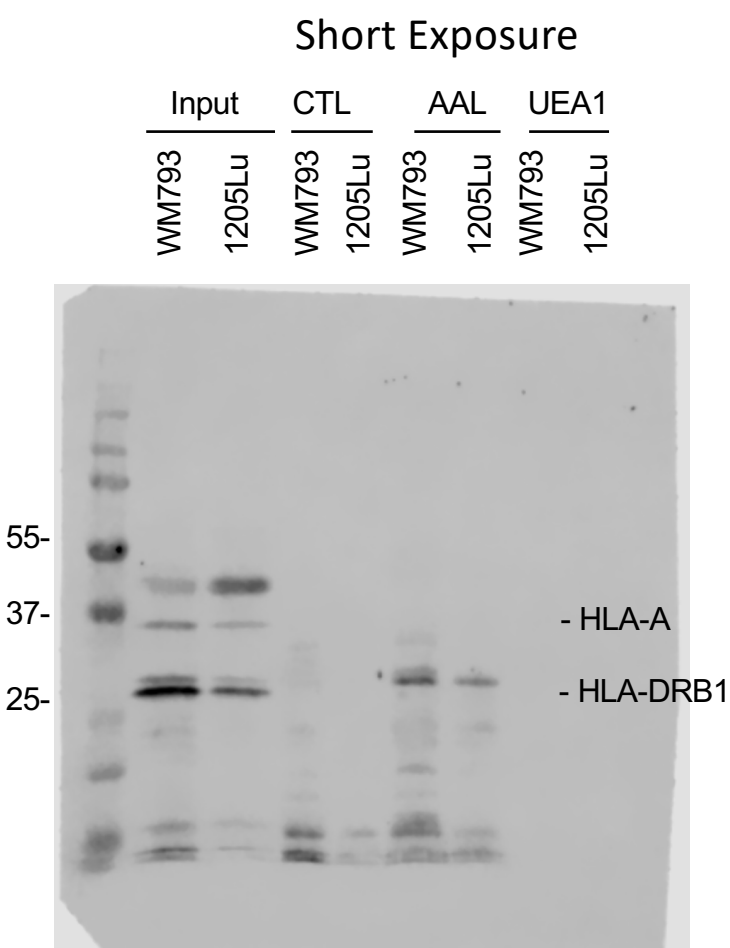

Figure 3B, right

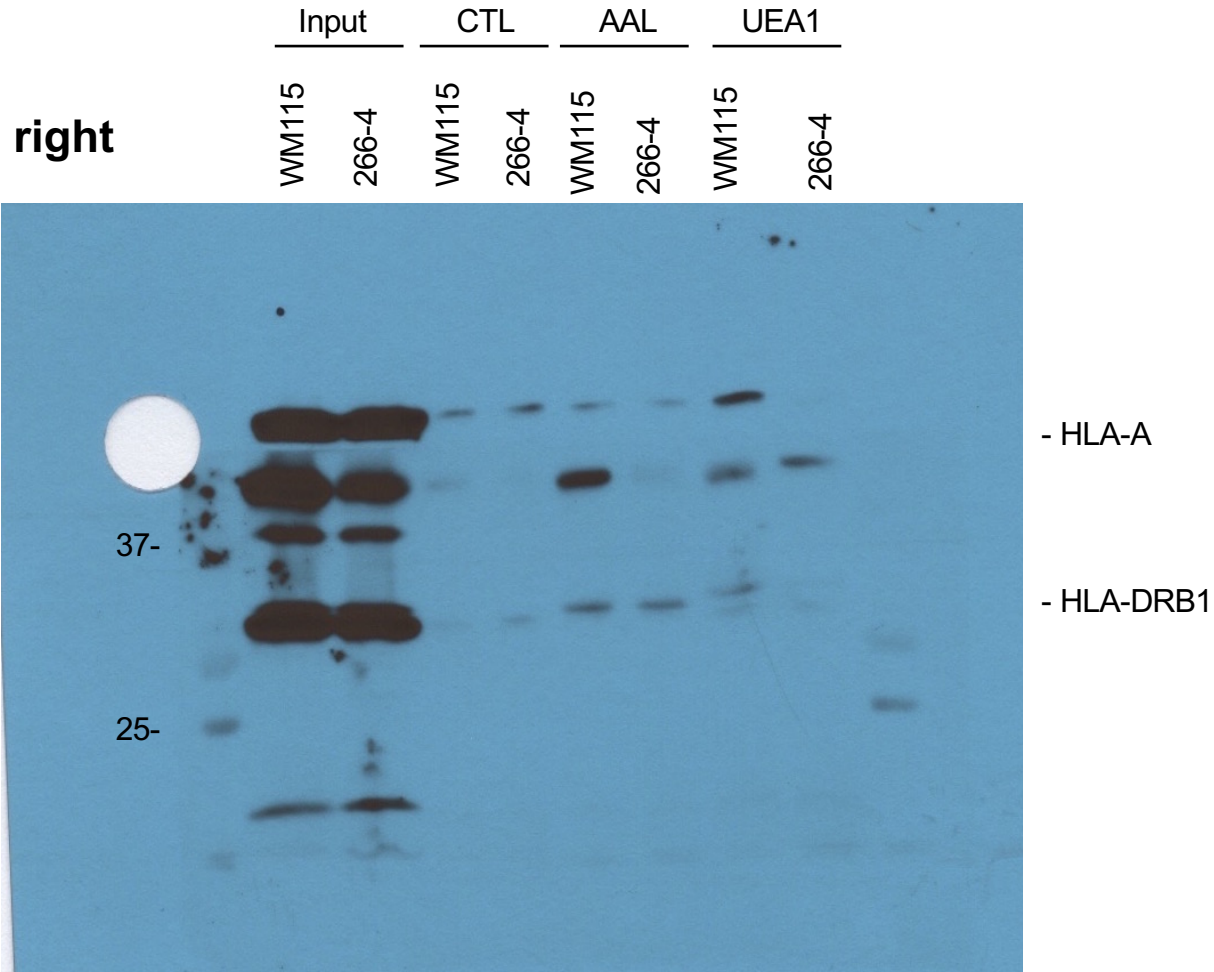

Figure 3C, left

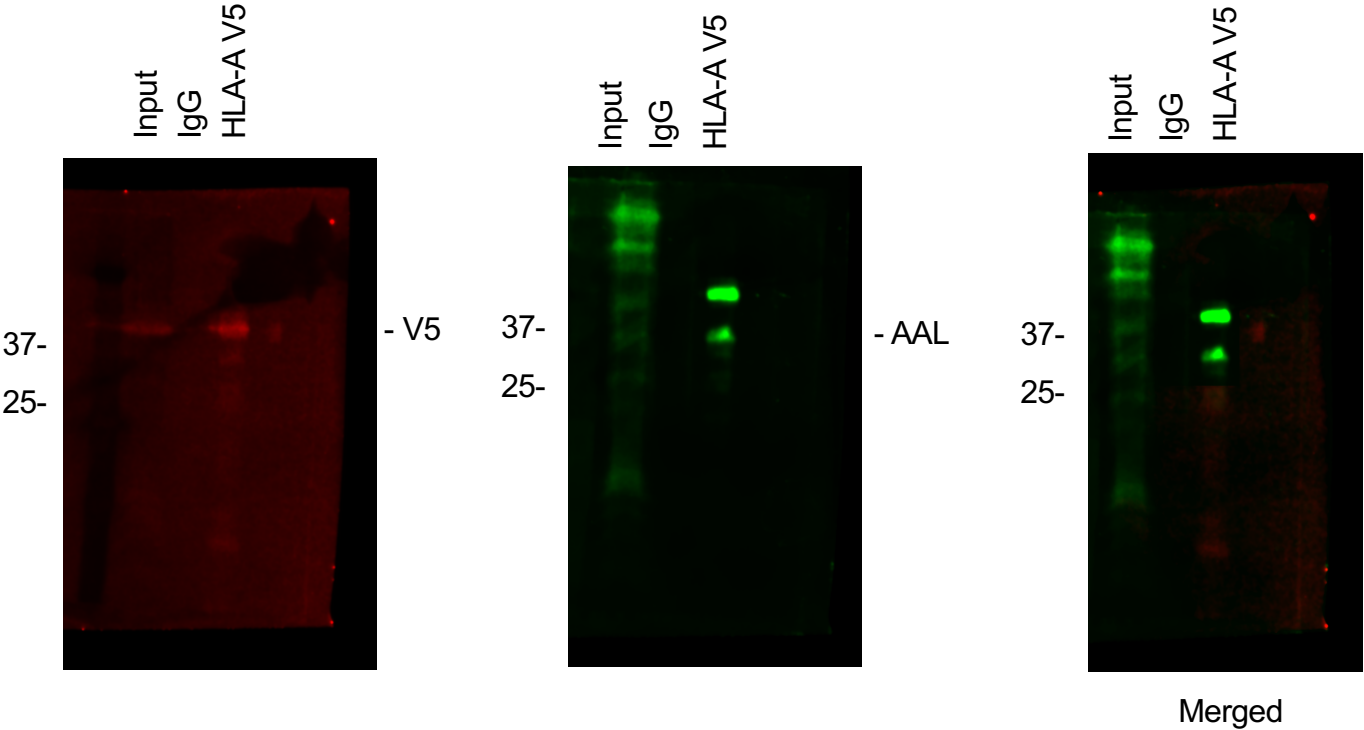

Figure 3C, right

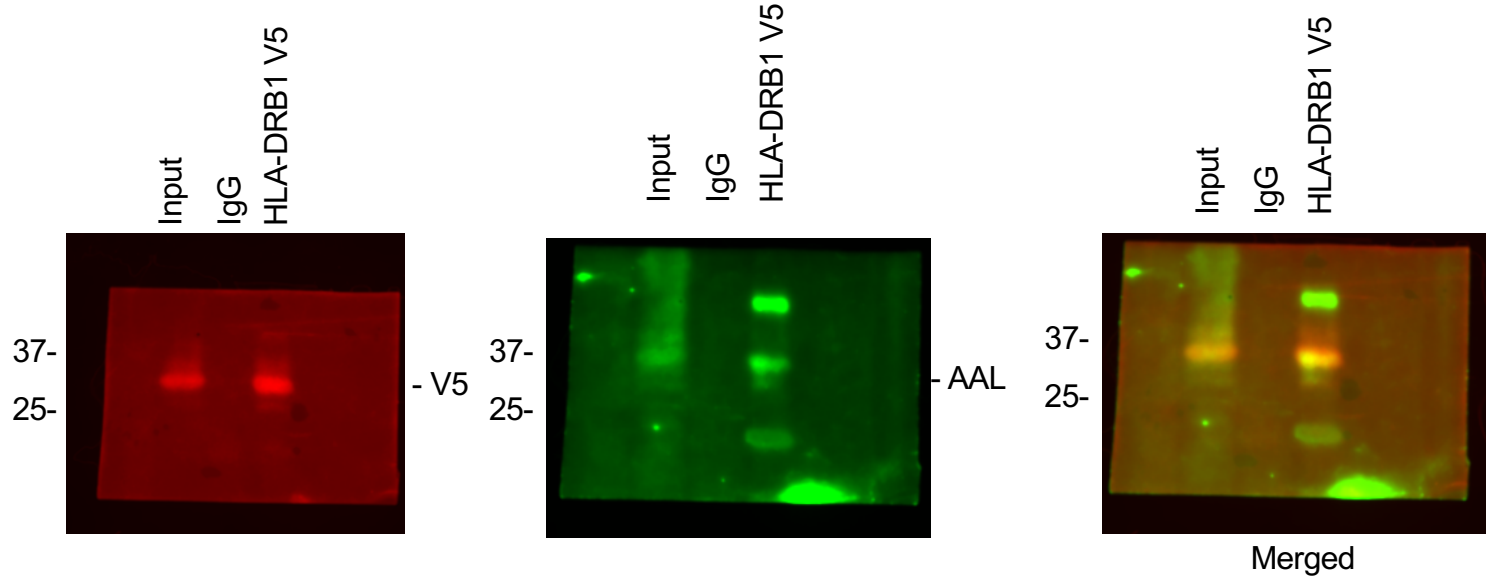

Supplement: Source Data Fig. 3 — Unprocessed blots for Fig. 3. [file 43018_2022_506_MOESM5_ESM.pdf]

Figure 4C

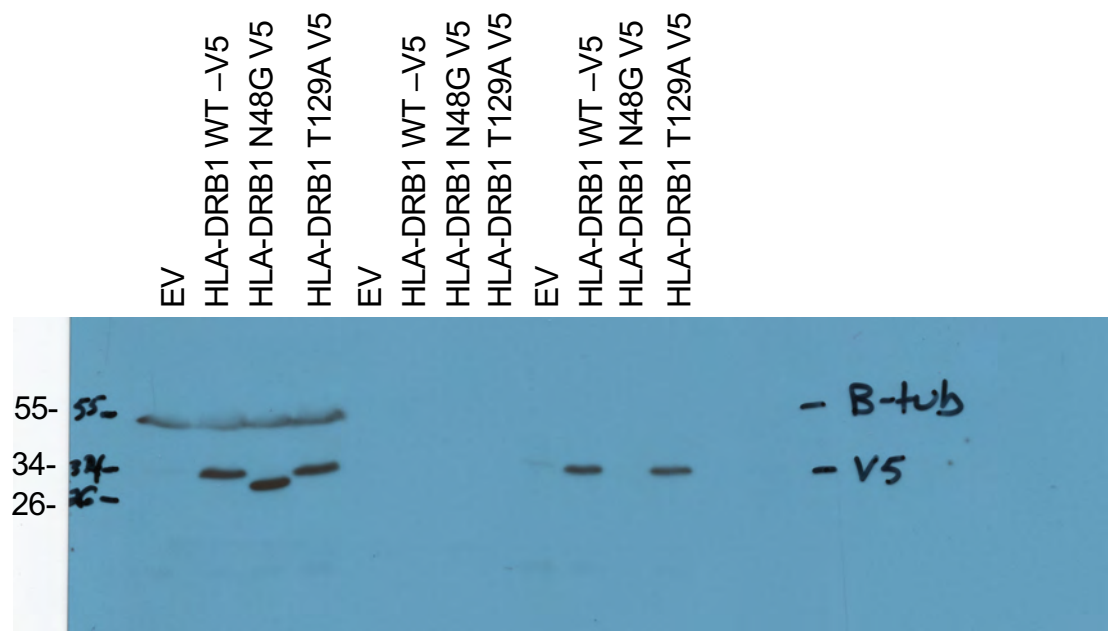

Figure 4E (WM793)

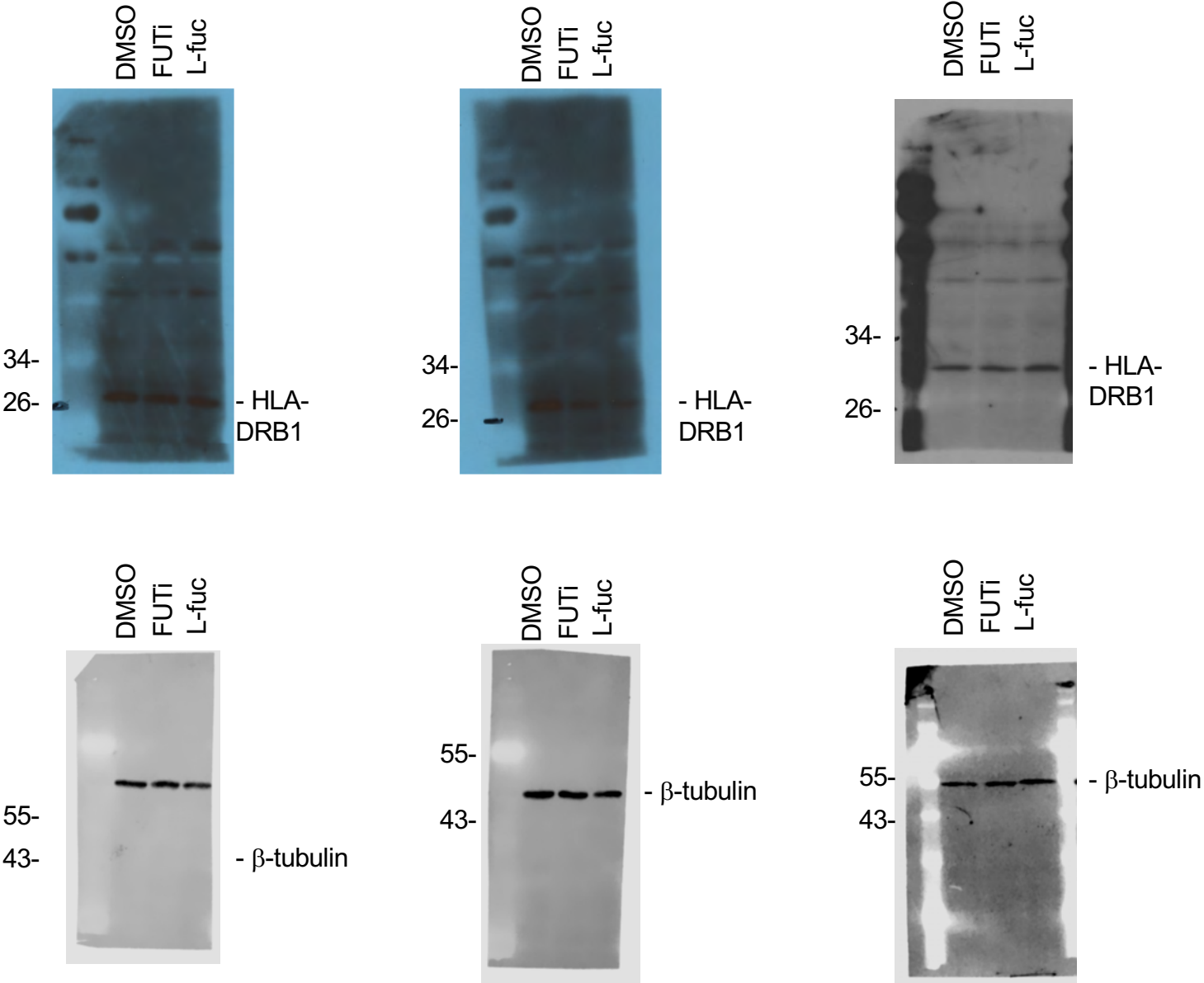

Figure 4E (1205Lu)

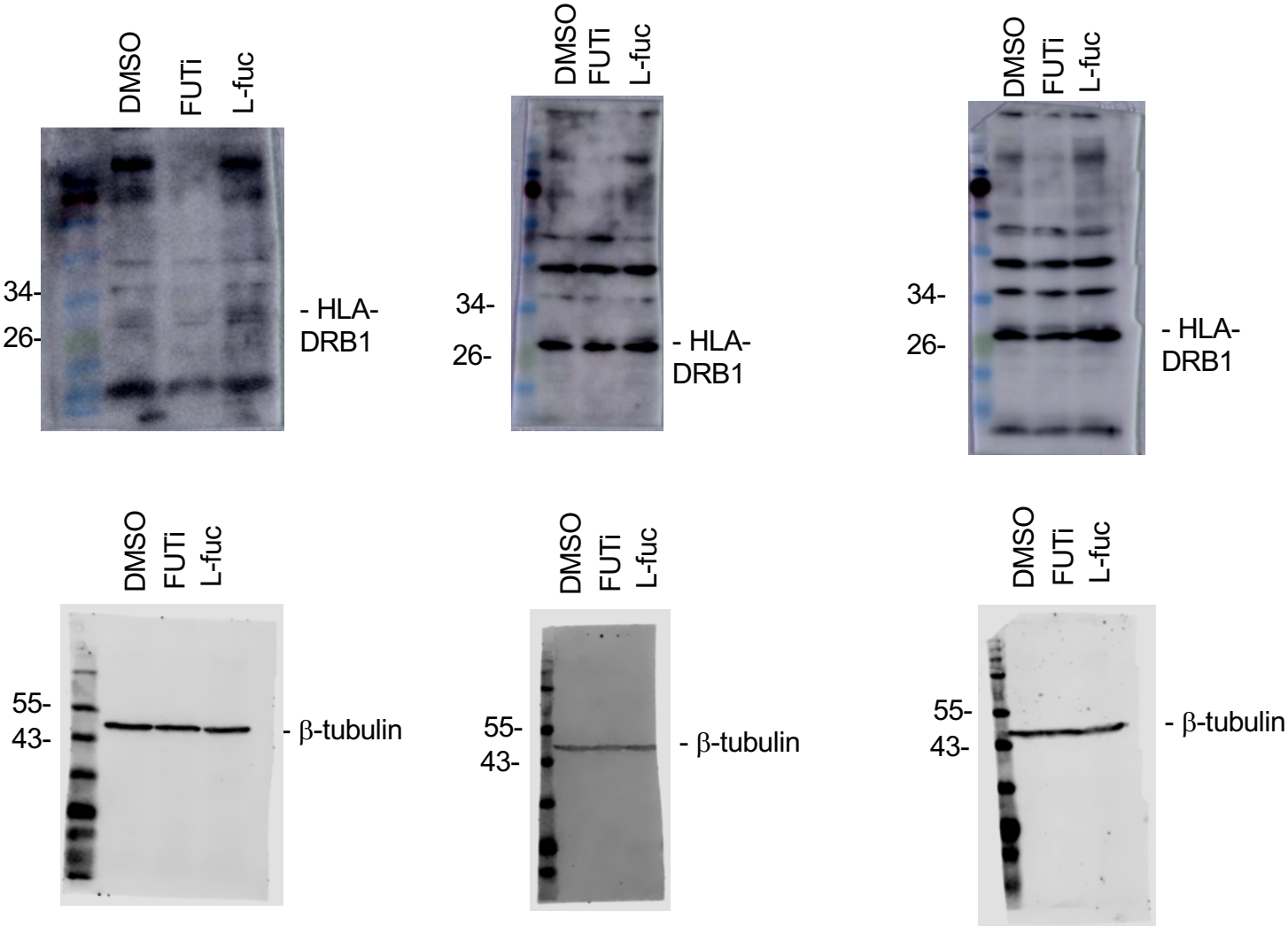

Supplement: Source Data Fig. 4 — Unprocessed blots for Fig. 4. [file 43018_2022_506_MOESM6_ESM.pdf]

Extended Data Figure 1L

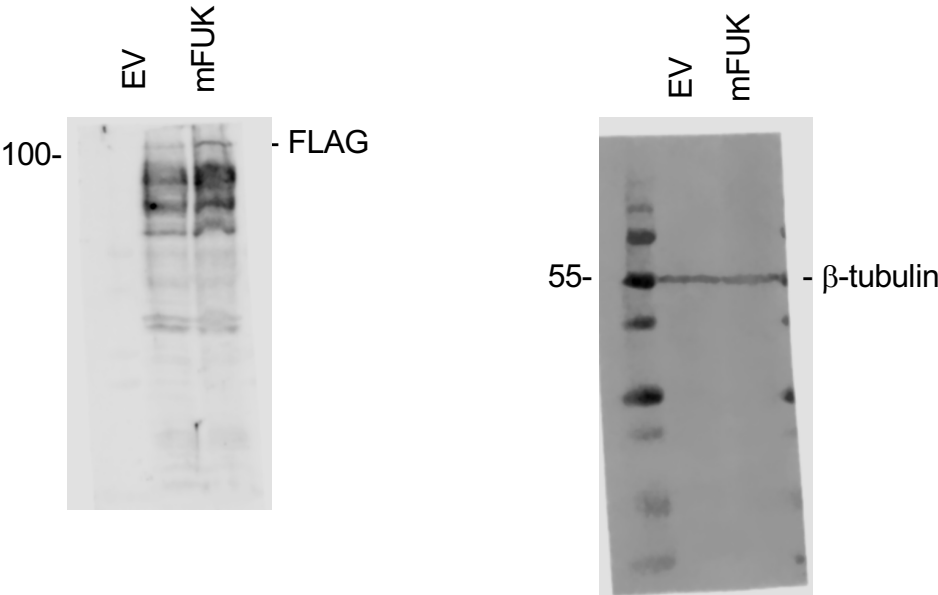

Supplement: Source Data Extended Data Fig. 1 — Unprocessed blots for Extended Data Fig. 1. [file 43018_2022_506_MOESM7_ESM.pdf]

Extended Data Figure 5A

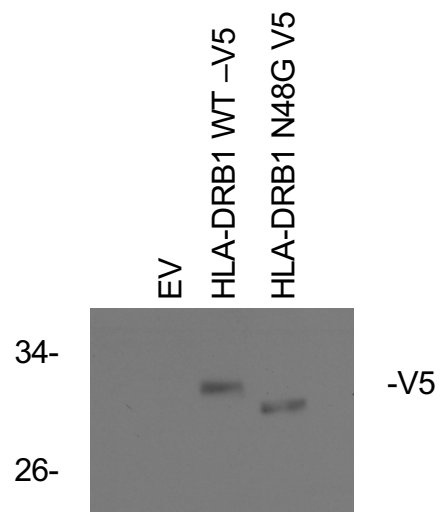

Extended Data Figure 5D

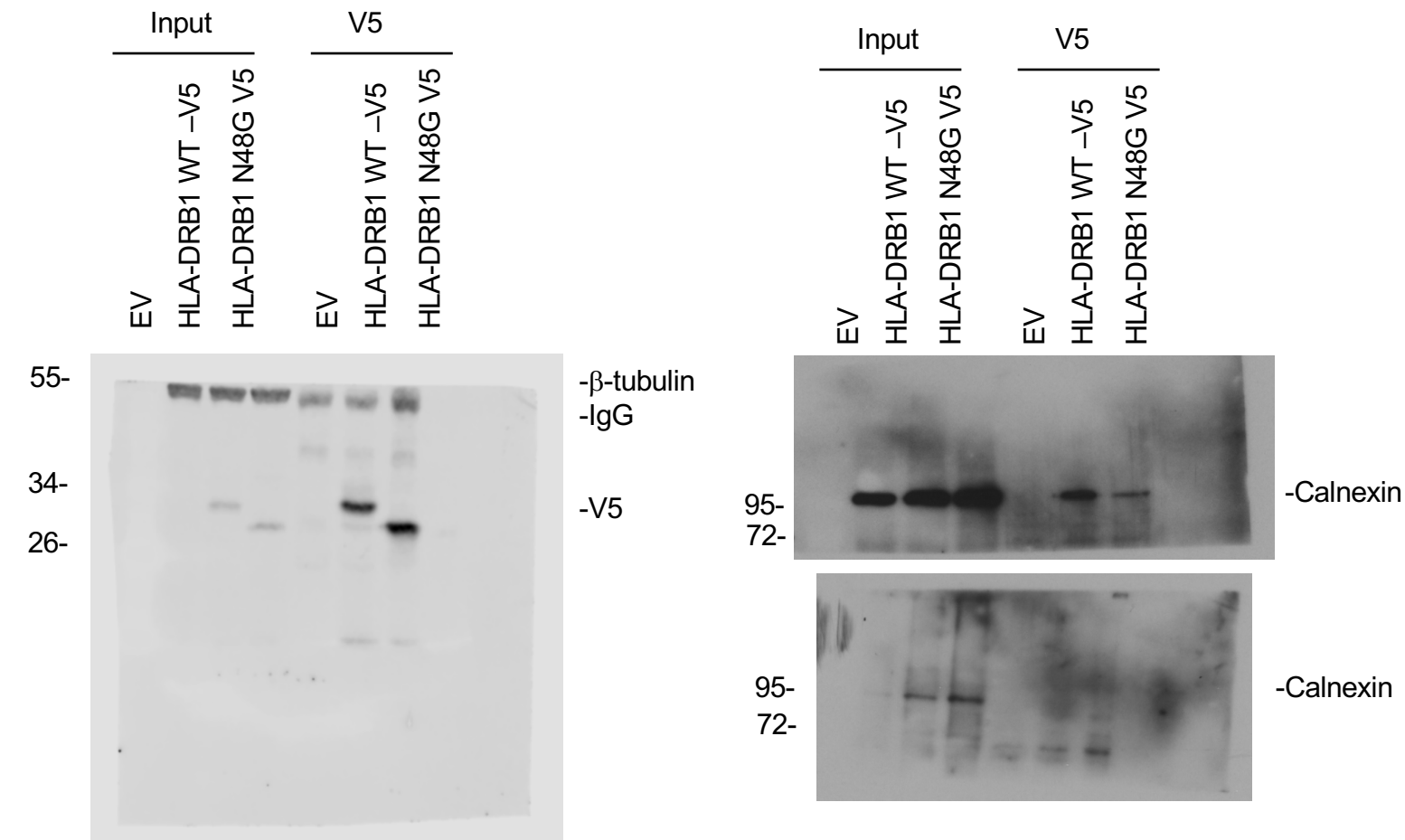

Extended Data Figure 5E

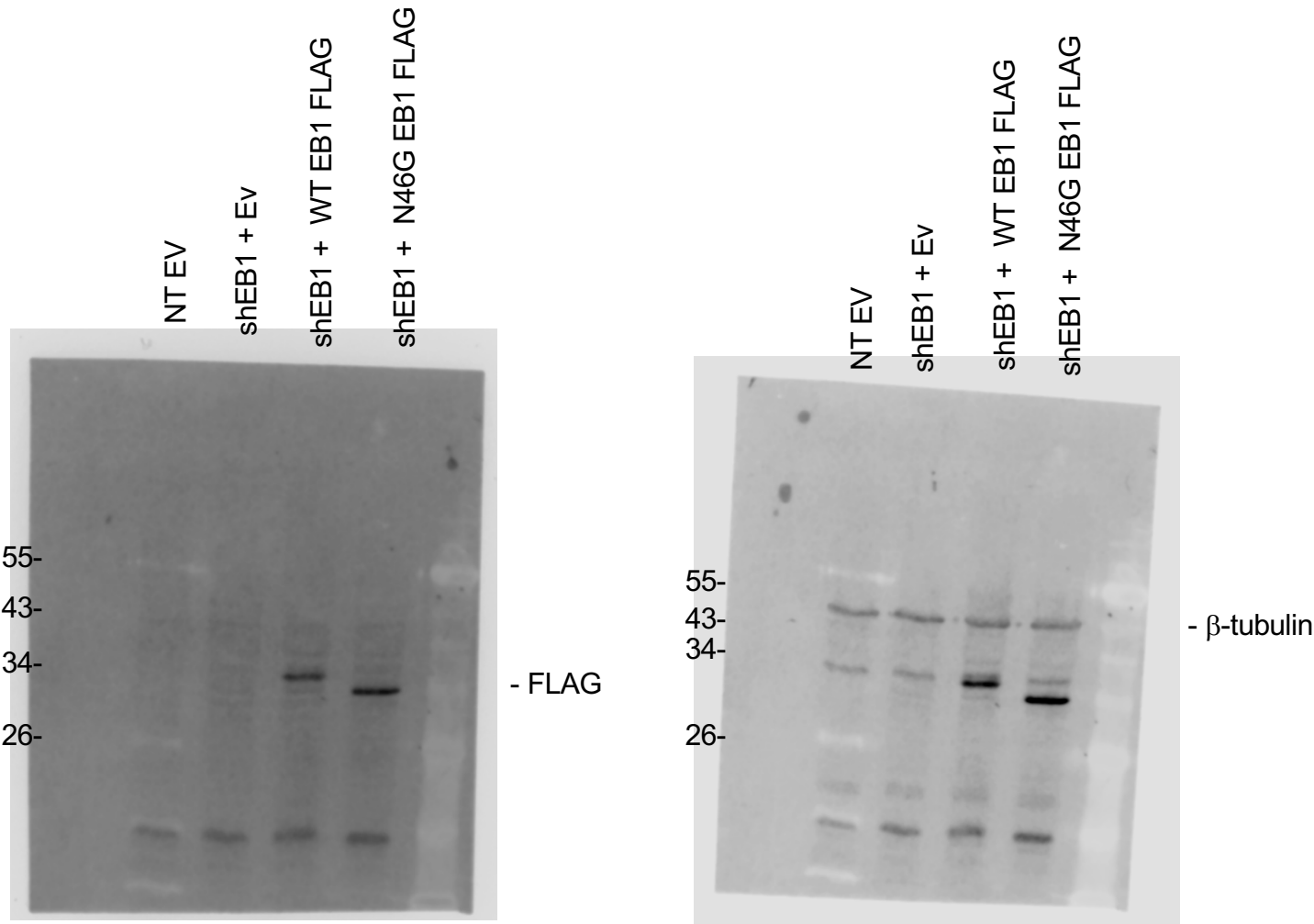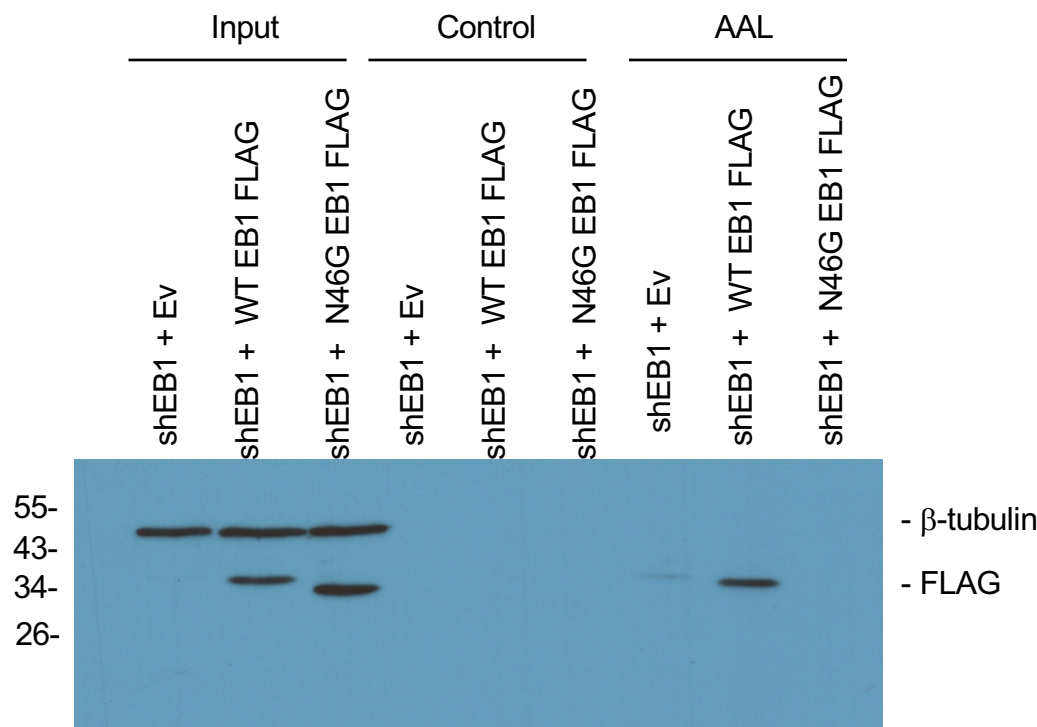

Supplement: Source Data Extended Data Fig. 5 — Unprocessed blots for Extended Data Fig. 5. [file 43018_2022_506_MOESM10_ESM.pdf]
