# Supplementary material for: Fucosylation of HLA-DRB1 regulates CD4+ T cell-mediated anti-melanoma immunity and enhances immunotherapy efficacy
Source: Nat Cancer. 2023 Jan 23;4(2):222–39. doi: 10.1038/s43018-022-00506-7 (PMC9970875; doi:10.1038/s43018-022-00506-7)

Extended Data Figure 2B

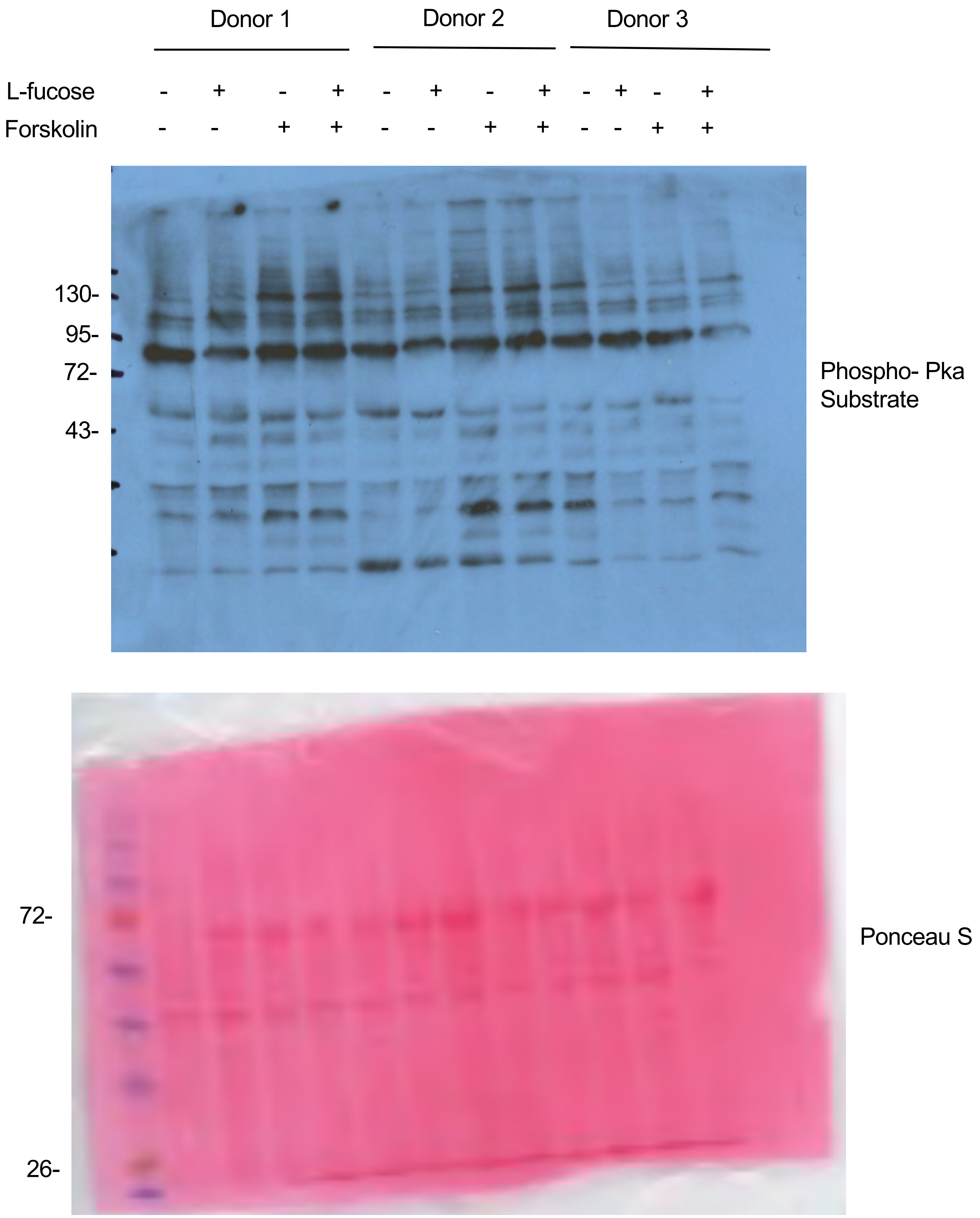

Extended Data Figure 2C

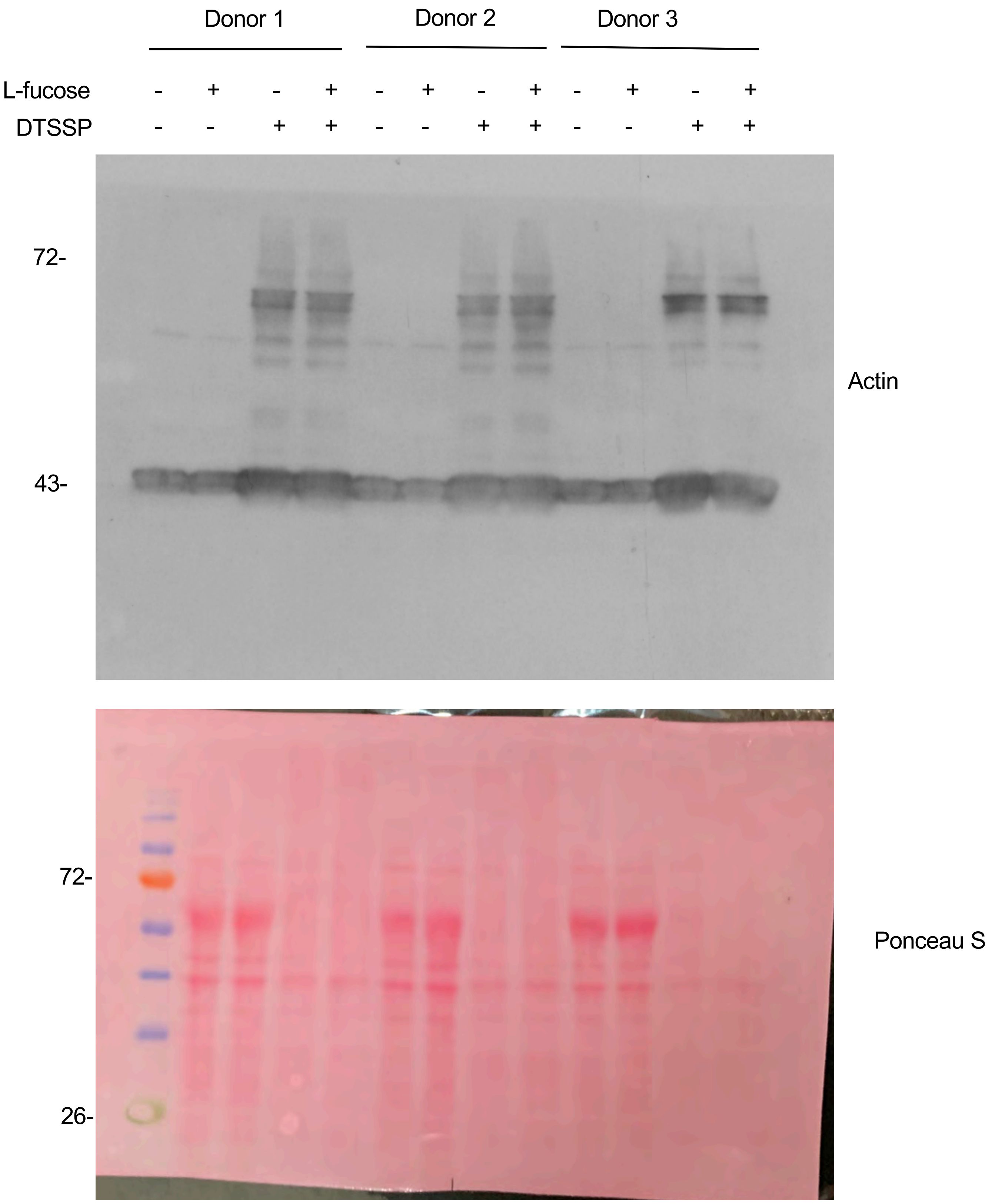

Extended Data Figure 2F

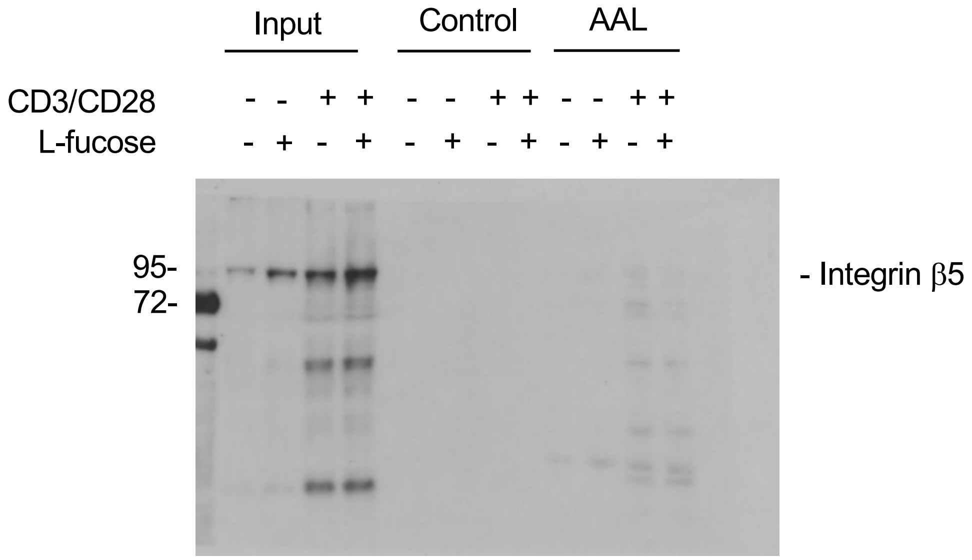

Supplement: Source Data Extended Data Fig. 2 — Unprocessed blots for Extended Data Fig. 2. [file 43018_2022_506_MOESM8_ESM.pdf]
